# Hybkit: a Python API and command-line toolkit for hybrid sequence data from chimeric RNA methods

**DOI:** 10.1093/bioinformatics/btad721

**Published:** 2023-11-25

**Authors:** Daniel Stribling, Lauren A Gay, Rolf Renne

**Affiliations:** Department of Molecular Genetics and Microbiology, University of Florida, Gainesville, FL 32610, United States; UF Genetics Institute, University of Florida, Gainesville, FL 32610, United States; UF Health Cancer Center, University of Florida, Gainesville, FL 32610, United States; Department of Molecular Genetics and Microbiology, University of Florida, Gainesville, FL 32610, United States; Department of Molecular Genetics and Microbiology, University of Florida, Gainesville, FL 32610, United States; UF Genetics Institute, University of Florida, Gainesville, FL 32610, United States; UF Health Cancer Center, University of Florida, Gainesville, FL 32610, United States

## Abstract

**Summary:**

Experimental methods using microRNA/target ligation have recently provided significant insights into microRNA functioning through generation of chimeric (hybrid) RNA sequences. Here, we introduce Hybkit, a Python3 API, and command-line toolkit for analysis of hybrid sequence data in the “hyb” file format to enable customizable evaluation and annotation of hybrid characteristics. The Hybkit API includes a suite of python objects for developing custom analyses of hybrid data as well as miRNA-specific analysis methods, built-in plotting of analysis results, and incorporation of predicted miRNA/target interactions in Vienna format.

**Availability and implementation:**

Hybkit is provided free and open source under the GNU GPL license at github.com/RenneLab/hybkit and archived on Zenodo (doi.org/10.5281/zenodo.7834299). Hybkit distributions are also provided via PyPI (pypi.org/project/hybkit), Conda (bioconda.github.io/recipes/hybkit/README.html), and Docker (quay.io/repository/biocontainers/hybkit).

## Graphical Abstract

**Figure:**
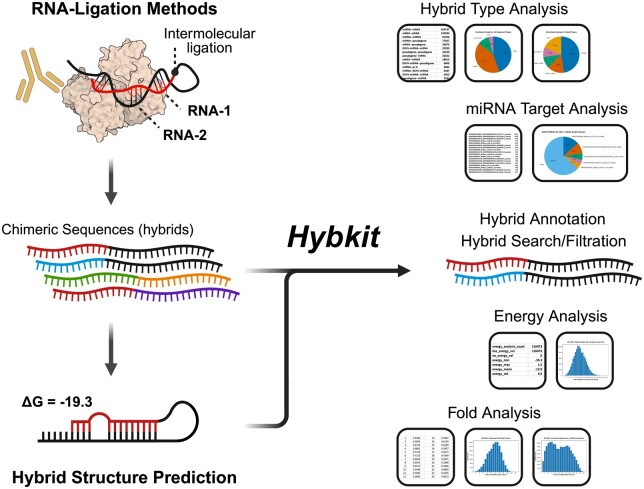
The Hybkit toolkit provides a suite of command-line utilities and Python3 API for analysis of chimeric (hybrid) sequence data from RNA ligation methods.

## 1 Introduction

MicroRNAs (miRNAs) are short, ∼18 to 22 nucleotide sequences that play a major role in post-transcriptional gene regulation by binding and inhibiting or degrading target RNAs (Bartel 2004, 2009). Over the past decade, several experimental approaches have been developed for characterization of miRNA targets including Cross-Linking, Ligation, and Sequencing of Hybrids (CLASH) (Kudla *et al.* 2011, Helwak *et al.* 2013, Helwak and Tollervey 2014), its derivative method Quick CLASH (qCLASH) (Gay *et al.* 2018a), CLEAR-CLIP (Moore *et al.* 2015), and chimeric e-CLIP (Manakov *et al.* 2022). Building on earlier RNA/protein-crosslinking approaches (Chi *et al.* 2009, Hafner *et al.* 2010), these methods capture intermolecular interactions by in-situ ligation of miRNAs to targets followed by high-throughput sequencing of resulting chimeric RNAs (hybrids). Since their development, the CLASH and qCLASH methods have provided significant insights into miRNA function in multiple viral infections and cancers (Gay *et al.* 2018a,b, Sethuraman *et al.* 2018, Bullard *et al.* 2019, Gay *et al.* 2021, Kozar *et al.* 2021, Ungerleider *et al.* 2021, Gureghian *et al.* 2023), investigated the role of miRNAs with noncanonical biogenesis (Fields *et al.* 2021), significantly expanded the set of known miRNAs involved in target RNA-directed miRNA degradation (TDMD) (Li *et al.* 2021, Sheng *et al.* 2023), and enabled discovery of a novel human miRNA involved in carcinogenesis (Stribling *et al.* 2021). Hybrid read identification for CLASH experiments has primarily been performed via the Hyb pipeline, with output of identified hybrids in the GFF-related “hyb” file format (Travis *et al.* 2014). However, a lack of analysis methodology for these hybrid sequences has impeded broad use of CLASH data by requiring de novo development of methods for most published studies.

Here, we introduce Hybkit, a toolkit, and Python3 API for analysis of hyb-format chimeric sequence data from RNA-ligation experiments. Hybkit enables the flexible classification and annotation of identified hybrid segments, identification of miRNA-containing hybrids, and filtration of records based on sequence identifiers and other annotation information.

Built-in plotting features allow visualization of analysis results, including plotting the distributions of segment types and miRNA targets. In addition to sequence data, Hybkit also includes methods for merging information from hyb files with corresponding predicted molecular secondary structure (“fold”) files in the Vienna format produced by ViennaRNA or UNAFold (Markham and Zuker 2008, Lorenz *et al.* 2011). This allows analysis and per-nucleotide visualization of predicted miRNA binding patterns, providing insight into potential miRNA/target affinity and functionality of miRNA/target interactions. We additionally provide a file-format specification for “hyb” files for standardized file parsing and annotation.

## 2 Analysis capabilities

**Figure 1.**
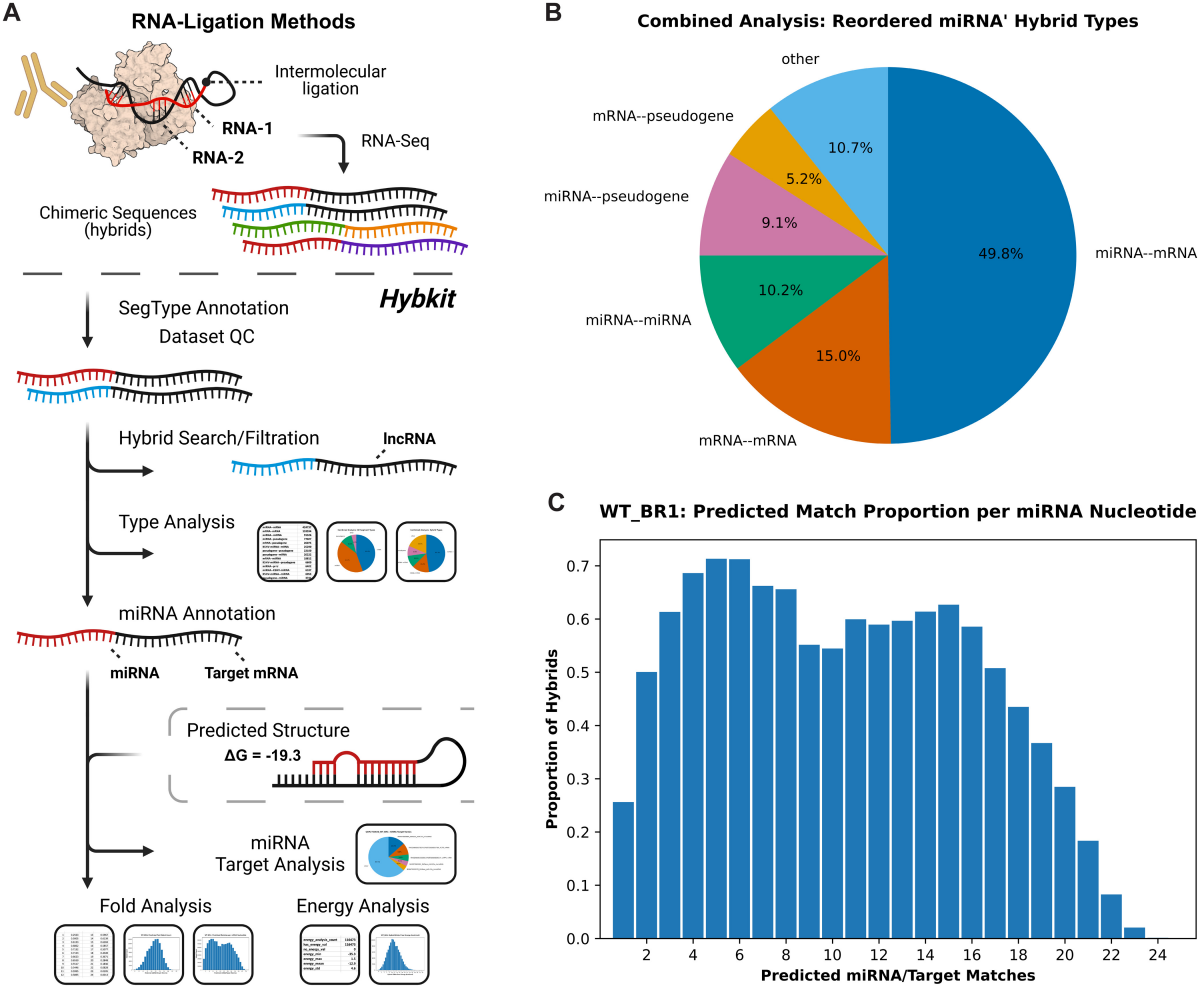
Hybkit analysis workflow for an example hybrid sequence dataset. RNA-ligation ribonomics methods such as CLASH, qCLASH, and chimeric e-CLIP generate chimeric (hybrid) RNA sequences identified by a hybrid caller. Hybkit provides a suite of tools for filtration, annotation, and analysis of hybrid sequence datasets following hybrid identification. (A) Flow-chart depicting the workflow for an example Hybkit analysis. Segment-type (SegType) annotation classifies individual hybrid segments, allowing dataset filtration and hybrid searching based on segment type. For microRNA- (miRNA-) focused ligation methods, miRNA annotation allows hybrid-type and miRNA target analyses of hybrids. Hybkit also incorporates predicted hybrid secondary structure data in Vienna format, allowing analysis of predicted segment interaction stability and nucleotide binding patterns. (B) The segment-type distribution of grouped replicates of a qCLASH experiment from *Example Analysis 1*. (C) the per-nucleotide proportion of hybrids predicted to be bound to their respective targets from *Example Analysis 4.*

Hybkit includes several built-in methods for evaluation of hybrid sequence records and assigned segment types, depicted in an example analysis workflow in Fig. 1A. Hybrid segment classification is performed via a user-selectable method based on segment identifiers, with resulting type annotations appended to the record “flags” string. Predefined methods include Hyb-format identifier splitting for the Hyb pipeline hOH7 reference library (Travis *et al.* 2014), as well as custom segment ID pattern matching, direct mapping of identifiers to sequence types via a csv file, and custom user-defined methods for identifying segment type. Following segment classification, several evaluation capabilities are included for miRNA-containing hybrids, including annotation of miRNA 5′ or 3′ position within the hybrid, miRNA/target subsequence extraction, and miRNA-specific analysis of secondary structure characteristics.

Following segment-type and miRNA evaluation, Hybkit includes multiple descriptive analyses for hybrid datasets. These include predicted intramolecular energy of folding, distribution of observed sequence-pair types, distribution of miRNA/target types, analysis of individual miRNA targets, and characterization of predicted binding patterns between miRNAs and targets. Each analysis allows optional plotting of results with Matplotlib (Hunter 2007) as shown with plots from included example analyses in Fig. 1B and C. Color-containing plots are generated using the Bang Wong color palette for increased accessibility by colorblind individuals (Wong 2011).

## 3 Python API

The Hybkit API provides a documented suite of Python3 classes and methods for interacting with hyb-format data inspired by the BioPython project (Cock *et al.* 2009). Primary API functionality is provided by the HybRecord and HybFile classes and their associated methods. Each HybRecord stores information about an individual hybrid, including source read identifier and nucleotide sequence as well as associated annotations and analysis results. The HybRecord class provides built-in methods for evaluation and recall of segment-type designations, miRNA annotations, and other details stored in the flags string. The HybFile class allows parsing of a hyb-format file as a python file object, allowing iteration over hybrids as HybRecords and writing HybRecord objects directly to hyb files.

Hyb-format files are often paired with corresponding information on predicted molecular secondary structure stored in the Vienna dot-bracket file format (Lorenz *et al.* 2011), including predicted intramolecular folding pattern and Gibbs Free Energy. In parallel with the hyb-classes above, Hybkit provides FoldRecord and ViennaFile classes for reading and analyzing secondary structure information in Vienna format. An additional HybFoldIter class is provided for concurrent iteration over hyb and Vienna files and includes methods to merge FoldRecord data into corresponding HybRecords and check for potential errors and mismatches between respective sequences.

Hybkit analysis and plotting capabilities are directly accessible through the API using the Analysis class, which can apply multiple analysis types simultaneously. After instantiation, individual records are added to the class object which allows all analyses to be conducted in single iteration over input data concurrent with other processing and filtration steps. Examples for use of the API for data analysis are provided in the Section 5.

## 4 Command-line toolkit

In addition to the Python API, Hybkit includes several command-line tools for parsing, annotation, filtration, and analysis of hyb data within command-line workflows. These include *hyb_check* for error checking of hyb (and Vienna) files; *hyb_eval* for annotation of hybrid segment type, miRNA presence, and other details; *hyb_filter* for selection of hybrids with specific properties; and *hyb_analyze* for running descriptive analyses on annotated hyb files. Flags and settings are standardized across tools and correspond to associated settings in the Hybkit API, allowing for flexibility in implementing analyses using either method.

## 5 Example analyses

To demonstrate usage of Hybkit functions, several example analysis workflows are included. Each analysis demonstrates parsing, annotation, and filtration of one or more hyb files and/or corresponding Vienna files using Hybkit. Analyses are conducted using a publicly available dataset (see “Data availability” section) and include production of plots for each example. Each analysis is implemented as both a Python script using the Hybkit API and as a shell script using Hybkit command-line tools to demonstrate usage of both approaches. Example analyses include:

1. Analysis of type and miRNA characteristics across three qCLASH replicates.
2. Analysis of targets of Kaposi’s Sarcoma-Associated Herpesvirus (KSHV) miRNA kshv-miR-k12-5 in hybrids.
3. Grouped target analysis for all KSHV miRNAs for 6 qCLASH replicates.
4. Analysis of predicted energy of binding and patterns of intermolecular folding between miRNAs and targets in a qCLASH dataset.

## 6 Installation and usage

The Hybkit package is released free and open-source under the GNU GPL v3.0 license (or greater version) via GitHub at (github.com/RenneLab/hybkit) and archived on Zenodo (doi.org/10.5281/zenodo.7834299). Hybkit distributions are also available via the python package index (PyPI; pypi.org/project/hybkit), an Anaconda module hosted by the Bioconda project (bioconda.github.io/recipes/hybkit/README.html) (Anaconda 2016, Grüning *et al.* 2018), and as a Docker image through the BioContainers project (quay.io/repository/biocontainers/hybkit) (da Veiga Leprevost *et al.* 2017). An installation-test script is provided for testing of command-line utilities against expected results, with additional testing of Python3 API classes and methods via a suite of Python unit tests. Both command-line and unit tests are executed regularly via continuous integration testing on the project GitHub repository to ensure stability over successive release versions. Full documentation for the current version of the Python API and command line utilities and installation instructions for all distribution types are provided in the Hybkit documentation on ReadTheDocs (hybkit.readthedocs.io) with a copy of the v0.3.4 documentation provided in the Supplementary Data.

## 7 Conclusion

Hybkit provides a flexible suite of tools for analysis of hybrid sequence data from RNA-ligation experiments. In addition to predefined analyses, the toolkit supports custom command-line and programmatic workflows via a parallel API interface and suite of command-line utilities, providing both textual and graphical outputs to meet a variety of use cases. To enhance reproducibility of results, the package GitHub repository is maintained using CI testing and provides containerized package distributions. Hybkit also includes a full documentation for Python API classes and methods to allow extension of the project codebase and incorporation into future projects for hybrid sequence analysis. As such, Hybkit provides new analysis capability and increased reproducibility for hybrid sequence analysis, expanding the potential utility of RNA-ligation methods in RNA biology.

## Supplementary Material

btad721_Supplementary_Data

## Data Availability

The dataset used for example analyses (Gay *et al.* 2018a) is available via NCBI Gene Expression Omnibus accession GSE101978: identifiers.org/geo:GSE101978.
