# Supplementary material for: Hybkit: a Python API and command-line toolkit for hybrid sequence data from chimeric RNA methods
Source: Bioinformatics. 2023 Nov 25;39(12):btad721. doi: 10.1093/bioinformatics/btad721 (PMC10701094; doi:10.1093/bioinformatics/btad721)
Supplement: btad721_Supplementary_Data [file btad721_supplementary_data.pdf]

hybkit

Release v0.3.4

Daniel Stribling

Nov 20, 2023

DOCUMENTATION CONTENTS:

1 hybkit Hyb File Specification **5**
- 1.1 Columns . . . 6
- 1.2 Flags . . . 6
- 1.3 Other Details . . . 7
- 1.4 Example . . . 7

2 hybkit API **8**
- 2.1 hybkit (module) . . . 8
- 2.2 hybkit.type_finder . . . 34
- 2.3 hybkit.analysis . . . 40
- 2.4 hybkit.plot . . . 47
- 2.5 hybkit.settings . . . 51
- 2.6 hybkit.util . . . 56
- 2.7 hybkit.errors . . . 60

3 hybkit Toolkit **61**
- 3.1 hyb_check . . . 61
- 3.2 hyb_filter . . . 65
- 3.3 hyb_eval . . . 71
- 3.4 hyb_analyze . . . 77

4 Example Analyses **82**
- 4.1 Example Type-miRNA Analysis . . . 82
- 4.2 Example Target Analysis . . . 85
- 4.3 Example Grouped Target Analysis . . . 86
- 4.4 Example Fold Analysis . . . 87

5 About **91**
- 5.1 Renne Lab . . . 91
- 5.2 Lead Developer . . . 91
- 5.3 Changelog . . . 91

6 References **94**

Bibliography **95**

Python Module Index **96**

Index **97**

[1] [2] [3] [4] [5] [6] [7] [8] [9] [10]

Welcome to *hybkit*, a toolkit for analysis of hyb-format chimeric (hybrid) RNA sequence data defined with the Hyb software package by *Travis et al. (Methods 2014)*[11]. This genomic data-type is generated from RNA proximity-ligation and ribonomics techniques such as Crosslinking, Ligation, and Sequencing of Hybrids (CLASH; *Helwak et al. (Cell 2013)*[12]) and quick CLASH (qCLASH; *Gay et al. (J. Virol. 2018)*[13]).

This software is available via Github, at http://www.github.com/RenneLab/hybkit .

Full project documentation is available at *this documentation* (page **??**).

Project components:

1. hybkit toolkit of command-line utilities for manipulating, analyzing, and plotting hyb-format data.
2. The hybkit python API, an extendable documented codebase for creation of custom analyses of hyb-format data.
3. Integrated analysis of predicted secondary structure (fold) information for the API and command-line utilities.
4. Example analyses for publicly available qCLASH hybrid sequence data implemented in each of the command-line scripts and hybkit Python API.

Hybkit Toolkit:

The hybkit toolkit includes several command-line utilities for manipulation of hyb-format data:

Utility	Description
hyb_check	Parse hyb (and fold) files and check for errors
hyb_eval	Evaluate hyb (and fold) records to identify / assign segment types and miRNAs using custom criteria
hyb_filter	Filter hyb (and fold) records to a specific custom subset
hyb_analyze	Perform an energy, type, miRNA, target, or fold analysis on hyb (and fold) files and plot results

These scripts are used on the command line with hyb (and associated "vienna" or "CT") files. For example, to filter a hyb and corresponding vienna file to contain only hybrids with a sequence identifier containing the string "kshv":

Example:

```
$ hyb_filter -i my_hyb_file.hyb -f my_hyb_file.vienna --filter any_seg_
↪contains kshv
```

Further detail on the usage of each script is provided in the *hybkit Toolkit* (page 61) section of *this documentation* (page **??**).

[1] https://github.com/RenneLab/hybkit/releases
[2] https://pypi.org/project/hybkit/
[3] http://bioconda.github.io/recipes/hybkit/README.html
[4] http://bioconda.github.io/recipes/hybkit/README.html
[5] https://quay.io/repository/biocontainers/hybkit?tab=tags
[6] https://app.circleci.com/pipelines/github/RenneLab/hybkit
[7] https://hybkit.readthedocs.io/en/latest/?badge=latest
[8] https://coveralls.io/github/RenneLab/hybkit
[9] https://pypi.org/project/hybkit/
[10] https://www.gnu.org/licenses/gpl-3.0.en.html
[11] https://doi.org/10.1016/j.ymeth.2013.10.015
[12] https://doi.org/10.1016/j.cell.2013.03.043
[13] https://doi.org/10.1128/JVI.02138-17

Hybkit API:

Hybkit provides a Python3 module with a documented API for interacting with records in hyb files and associated vienna or CT files. This capability was inspired by the BioPython Project[14]. The primary utility is provided by a class for hyb records (HybRecord), a class for fold records (FoldRecord), and file-iterator classes (HybFile, ViennaFile, CTFile, HybFoldIter). Record attributes can be analyzed, set, and evaluated using included class methods.

For example, a workflow to print the identifiers of only sequences within a ".hyb" file that contain a miRNA can be performed as such:

```
#!/usr/bin/env python3
import hybkit
in_file = '/path/to/my_hyb_file.hyb'

# Open a hyb file as a HybFile Object:
with hybkit.HybFile.open(in_file, 'r') as hyb_file:

    # Return each line in a hyb file as a HybRecord object
    for hyb_record in hyb_file:

        # Analyze each record to assign segment types
        hyb_record.eval_types()

        # If the record contains a long noncoding RNA type, print the record␣
↪identifier.
        if hyb_record.has_prop('any_seg_type_contains', 'lncRNA')
            print(hyb_record.id)
```

Further documentation on the hybkit API can be found in the *hybkit API* (page 8) section of *this documentation* (page **??**).

Example Analyses:

Hybkit provides several example analyses for hyb data using the utilities provided in the toolkit. These include:

Analysis	Description
Type/miRNA Analysis	Quantify sequence types and miRNA types in a hyb file
Target Analysis	Analyze targets of a set of miRNAs from a single experimental replicate
Grouped Target Analysis	Analyze and plot targets of a set of miRNAs from pooled experimental replicates
Fold Analysis	Analyze and plot predicted miRNA folding patterns in miRNA-containing hybrids

These analyses provide analysis results in both tabular and graph form. As an illustration, the example summary analysis includes the return of the contained hybrid sequence types as both a csv table and as a pie chart:

CSV Output[15]

[14] https://biopython.org/

[15] https://raw.githubusercontent.com/RenneLab/hybkit/master/example_01_type_mirna_analysis/example_output/combined_analysis_type_hybrid_types.csv

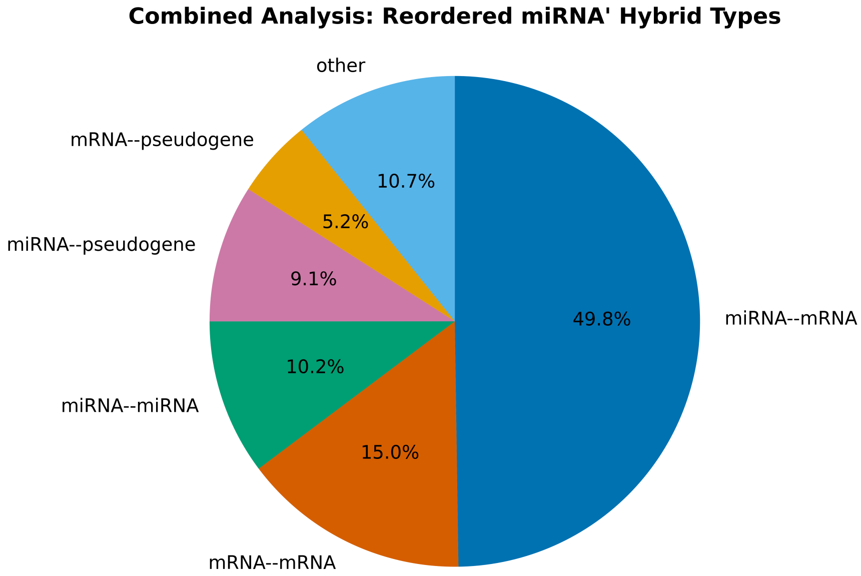

Further detail on each provided analysis can be found in the *Example Analyses* (page 82) section of *this documentation* (page **??**).

Installation:

Dependencies:

- Python3.8+
- matplotlib[16] >= 3.7.1 (*Hunter JD. (Computing in Science & Engineering 2007)*[17])
- BioPython[18] >= 1.79 (*Cock et al. (Bioinformatics 2009)*[19])
- *typing_extensions <https://pypi.org/project/typing-extensions/>* >= 4.8.0

Via PyPI / Python PIP:
[20]

The recommended installation method is via hybkit's PyPI Package Index[21] using python3 pip[22], which will automatically handle version control and dependency installation:

```
$ python3 -m pip install hybkit
```

Via Conda:
[23] [24]

For users of conda, the hybkit package and dependencies are hosted on the the Bioconda[25] channel, and can be installed using conda:

[16] https://matplotlib.org/
[17] https://doi.org/10.1109/MCSE.2007.55
[18] https://biopython.org/
[19] https://doi.org/10.1093/bioinformatics/btp163
[20] https://pypi.org/project/hybkit/
[21] https://pypi.org/project/hybkit/
[22] https://pip.pypa.io/en/stable/
[23] http://bioconda.github.io/recipes/hybkit/README.html
[24] http://bioconda.github.io/recipes/hybkit/README.html
[25] https://bioconda.github.io/

```
$ conda install -c bioconda hybkit
```

Via Docker/Singularity:
[26]

The hybkit package is also available as a Docker[27] image and Singularity[28] container, hosted via the BioContainers[29] project on quay.io[30]. To pull the image via docker:

```
$ docker pull quay.io/biocontainers/hybkit:0.3.3--pyhdfd78af_0
```

To pull the image via singularity:

```
$ singularity pull docker://quay.io/biocontainers/hybkit:0.3.3--pyhdfd78af_0
```

Manually Download and Install:
[31]

Use git to clone the project's Github repository:

```
$ git clone git://github.com/RenneLab/hybkit
```

OR download the zipped package:

```
$ curl -OL https://github.com/RenneLab/hybkit/archive/master.zip
$ unzip master.zip
```

Then install using python setuptools:

```
$ python setup.py install
```

Further documentation on hybkit usage can be found in *this documentation* (page **??**).

Setup Testing:

Hybkit provides a suite of unit tests to maintain stability of the API and script functionalities. To run the API test suite, install pytest and run the tests from the root directory of the hybkit package:

```
$ pip install pytest
$ pytest
```

Command-line scripts can be tested by running the auto_test.sh script in the auto_tests directory:

```
$ ./auto_tests/auto_test.sh
```

Copyright:

hybkit is a free, sharable, open-source project.

All source code and executable scripts contained within this package are considered part of the "hybkit" project and are distributed without any warranty or implied warranty under the GNU General Public License v3.0 or any later version, described in the "LICENSE" file.

[26] https://quay.io/repository/biocontainers/hybkit?tab=tags
[27] https://www.docker.com/
[28] https://sylabs.io/singularity/
[29] https://biocontainers.pro/
[30] https://quay.io/repository/biocontainers/hybkit?tab=tags
[31] https://github.com/RenneLab/hybkit/releases

CHAPTER

ONE

HYBKIT HYB FILE SPECIFICATION

Version: v0.3.4

The ".hyb" (hyb file) format is described by [Travis2014] along with the Hyb software package as a "gff-related format that contains sequence identifiers, read sequences, 1-based mapping coordinates, and annotation information for each chimera".

Each line in a hyb file (a hyb "record") contains information about an RNA sequence read identified as a chimera by an RNA hybridization analysis. Each line contains 15 or 16 columns separated by tab characters ("\t") and provides information on each of the two aligned segments identified within the sequence read. The columns are described as follows by [Travis2014]:

> Column 1, unique sequence identifier.
> Column 2, read sequence [...].
> Column 3, predicted binding energy in kcal/mol.
> Columns 4–9, mapping information for first fragment of read: name of matched transcript, coordinates in read, coordinates in transcript, mapping score.
> Columns 10–15, mapping information for second fragment of read.
> Column 16 (optional, [...]), list of annotations in the format: “feature1=value1; feature2=value2;..."

The hybkit project uses an extended version of this description, including assigning columns reference names, and defining allowed flags.

1.1 Columns

#	Name	Description
1	id	Hybrid Read Identifier
2	seq	Read Nucleotide Sequence
3	energy	Predicted Gibbs Free-Energy of Intra-Hybrid Folding
4	seg1_ref_name	Segment 1 Mapping Reference Identity
5	seg1_read_start	Segment 1 Mapping Start on Read
6	seg1_read_end	Segment 1 Mapping End on Read
7	seg1_ref_start	Segment 1 Mapping Start on Reference
8	seg1_ref_end	Segment 1 Mapping End on Reference
9	seg1_score	Segment 1 Mapping Score
10	seg2_ref_name	Segment 2 Mapping Reference Identity
11	seg2_read_start	Segment 2 Mapping Start on Read
12	seg2_read_end	Segment 2 Mapping End on Read
13	seg2_ref_start	Segment 2 Mapping Start on Reference
14	seg2_ref_end	Segment 2 Mapping End on Reference
15	seg2_score	Segment 2 Mapping Score
16	flags	Hybrid Read Analysis Details

1.2 Flags

Hyb Flags:

The following four flags are used by the Hyb software package ([Travis2014]). The definitions provided describe how these flags are used in the hybkit package.

`count_total` - Integer: Total represented hybrid records, if records have been combined.

`count_last_clustering` - Integer: Total represented hybrid records at last clustering.

`two_way_merged` - {"0" or "1"} Boolean representation of whether entries with mirrored 5' and 3' hybrids were merged if the record is a combined record.

`seq_IDs_in_cluster` - String: Comma-separated list of all record IDs of hybrids merged into this hybrid entry.

hybkit Flags:

The following flags are used by hybkit.

`read_count` - Integer: Number of sequence reads represented by this record. If the record is combined, this represents the total read count for all merged entries.

`orient` - String: Orientation of strand. Options: "F" (Forward), "IF" (Inferred Forward), "R" (Reverse), "IR" (Inferred Reverse), "U" (Unknown), or "IC" (Inferred Conflicting).

`seg1_type` - String: Assigned segment type of segment 1, ex: "miRNA" or "mRNA".

`seg2_type` - String: Assigned segment type of segment 2, ex: "miRNA" or "mRNA".

`seg1_det` - String: Arbitrary detail about segment 1.

`seg2_det` - String: Arbitrary detail about segment 2.

`miRNA_seg` - String: Indicates which (if any) segment mapping is a miRNA. Options are "N" (none), "5p" (seg1), "3p" (seg2), "B" (both), or "U" (unknown).

`target_reg` - String: Assigned region of the miRNA target. Options are "5pUTR", "C" ([C]oding), "3pUTR", "NON" ([NON]coding), "N" ([N]one), or "U" ([U]nknown).

`ext` - Integer: "0" or "1", Boolean representation of whether record sequences were bioinformatically extended as is performed by the Hyb software package.

`dataset` - String: Label for sequence dataset id (eg. source file), when combining records from different datasets.

1.3 Other Details

Item	Role
"\t" (tab)	Column Delimiter
"."	Missing Data Placeholder (equivalent to None)
".hyb"	File Suffix
".hyb.gz"	gzipped File Suffix
Disallowed	Header Lines
Disallowed	In-file Comments

1.4 Example

An example .hyb format line (courtesy of [Gay2018]):

```
2407_718    ATCACATTGCCAGGGATTTCCAATCCCCAACAATGTGAAAACGGCTGTC       .       MIMAT0000078_
↪MirBase_miR-23a_microRNA   1       21      1       21      0.0027  ENSG00000188229_
↪ENST00000340384_TUBB2C_mRNA     23      49      1181    1207    1.2e-06
```

CHAPTER TWO

HYBKIT API

The hybkit API provides a Python3 module with classes allowing parsing and manipulation of hyb-format data as python objects, including built-in analysis and plotting functionality for common tasks in hybrid sequence analysis.

hybkit (page 8)	Data classes for storing, evaluating, and iterating over records
settings (page 51)	Constants and settings information for hybkit classes and toolkit scripts
type_finder (page 34)	Class for customizable identification of segment type from reference identifiers
analysis (page 40)	Classes for predefined analyses of hyb records
plot (page 47)	Plotting methods for analysis results
util (page 56)	Support methods for executable scripts
errors (page 60)	Error classes for the hybkit package

2.1 hybkit (module)

Module storing primary hybkit classes and hybkit API.

This module contains classes and methods for reading, writing, and manipulating data in the hyb genomic sequence format ([Travis2014]). For more information, see the *hybkit Hyb File Specification* (page 5).

An example string of a hyb-format line from [Gay2018] is:

```
2407_718\tATCACATTGCCAGGGATTTCCAATCCCCAACAATGTGAAAACGGCTGTC\t.\tMIMAT0000078_MirBase_miR-
↪23a_microRNA\t1\t21\t1\t21\t0.0027\tENSG00000188229_ENST00000340384_TUBB2C_mRNA\t23\
↪t49\t1181\t1207\t1.2e-06
```

Hybkit functionality is primarily based on classes for storage and evaluation of chimeric genomic sequences and associated fold-information:

HybRecord (page 9)	Class to store a single hyb (hybrid) sequence record
FoldRecord (page 23)	Class to store predicted RNA secondary structure information for hybrid reads

Also included are classes for reading, writing, and iterating over files containing hybrid information:

HybFile (page 21)	Class for reading and writing hyb-format files [Travis2014] containing chimeric RNA sequence information as *HybRecord* (page 9) objects
ViennaFile (page 29)	Class for reading and writing Vienna-format files [ViennaFormat] containing RNA secondary structure information in dot-bracket format as *FoldRecord* (page 23) objects
CtFile (page 31)	-BETA- Class for reading Connectivity Table (.ct)-format files [CTFormat] containing predicted RNA secondary-structure information as used by UNAFold[32] as *FoldRecord* (page 23) objects
HybFoldIter (page 33)	Class for concurrent iteration over a *HybFile* (page 21) and a *ViennaFile* (page 29) or *CtFile* (page 31)

2.1.1 HybRecord Class

class hybkit.**HybRecord**(*id: str[33], seq: str[34], energy: Optional[35][Union[36][float[37], int[38], str[39]]] = None, seg1_props: Optional[40][Dict[41][str[42], Union[43][float[44], int[45], str[46]]]] = None, seg2_props: Optional[47][Dict[48][str[49], Union[50][float[51], int[52], str[53]]]] = None, flags: Optional[54][Dict[55][str[56], Any[57]]] = None, read_count: Optional[58][int[59]] = None, allow_undefined_flags: Optional[60][bool[61]] = None*)

Class for storing and analyzing chimeric (hybrid) RNA-seq reads in hyb format.

Hyb file (hyb) format entries are a GFF-related file format described by [Travis2014] that contain information about a genomic sequence read identified to be a hybrid by a chimeric read caller. Each line contains 15 or 16 columns separated by tabs ("\t") and provides annotations on each component. An example hyb-format line from [Gay2018]:

```
2407_718\tATCACATTGCCAGGGATTTCCAATCCCCAACAATGTGAAAACGGCTGTC\t.\tMIMAT0000078_
↪MirBase_miR-23a_microRNA\t1\t21\t1\t21\t0.0027\tENSG00000188229_ENST00000340384_
↪TUBB2C_mRNA\t23\t49\t1181\t1207\t1.2e-06
```

The columns are respectively described in hybkit as:

> id, seq, energy, seg1_ref_name, seg1_read_start, seg1_read_end, seg1_ref_start, seg1_ref_end, seg1_score, seg2_ref_name, seg2_read_start, seg2_read_end, seg2_ref_start, seg2_ref_end, seg2_score, flags

(For more information, see the *hybkit Hyb File Specification* (page 5))

The preferred method for reading hyb records from lines is with the *HybRecord.from_line()* (page 17) constructor:

```
# line = "2407_718\tATC..."
hyb_record = hybkit.HybRecord.from_line(line)
```

This is the constructor used by the *HybFile* (page 21) class to parse hyb files. For example, to print all hybrid identifiers in a hyb file:

```
with hybkit.HybFile('path/to/file.hyb', 'r') as hyb_file:
    # performs "hyb_record = hybkit.HybRecord.from_line(line)" for each line in file
    for hyb_record in hyb_file:
        print(hyb_record.id)
```

HybRecord objects can also be constructed directly. A minimum amount of data necessary for a HybRecord object is the genomic sequence and its corresponding identifier.

[32] http://www.unafold.org/

Examples

```
hyb_record_1 = hybkit.HybRecord('1_100', 'ACTG')
hyb_record_2 = hybkit.HybRecord('2_107', 'CTAG', '-7.3')
hyb_record_3 = hybkit.HybRecord('3_295', 'CTTG', energy='-10.3')
```

Details about segments are provided via python dictionaries with *keys* (page 11) specific to each segment. Data can be provided either as strings or as floats/integers (where appropriate). For example, to create a HybRecord object representing the example line given above:

```
seg1_props = {'ref_name': 'MIMAT0000078_MirBase_miR-23a_microRNA',
              'read_start': '1',
              'read_end': '21',
              'ref_start': '1',
              'ref_end': '21',
              'score': '0.0027'}
seg2_props = {'ref_name': 'ENSG00000188229_ENST00000340384_TUBB2C_mRNA',
              'read_start': 23,
              'read_end': 49,
              'ref_start': 1181,
              'ref_end': 1207,
              'score': 1.2e-06}
seq_id = '2407_718'
seq = 'ATCACATTGCCAGGGATTTCCAATCCCCAACAATGTGAAAACGGCTGTC'
energy = None

hyb_record = hybkit.HybRecord(seq_id, seq, energy, seg1_props, seg2_props)
# OR
hyb_record = hybkit.HybRecord(seq_id, seq, seg1_props=seg1_props, seg2_props=seg2_
↪props)
```

Parameters

- **id** (*str*[62]) -- Identifier for the hyb record
- **seq** (*str*[63]) -- Nucleotide sequence of the hyb record
- **energy** (str[64] or float[65], optional) -- Predicted energy of sequence folding in kcal/mol
- **seg1_props** (dict[66], optional) -- Properties of segment 1 of the record, containing possible *segment column* (page 11) keys: (ref_name, read_start, read_end, ref_start, ref_end, score)
- **seg2_props** (dict[67], optional) -- Properties of segment 2 of the record, containing possible: *segment column* (page 11) keys: (ref_name, read_start, read_end, ref_start, ref_end, score)
- **flags** (dict[68], optional) -- Dict with keys of flags for the record and their associated values. By default flags must be defined in *ALL_FLAGS* (page 11) but custom flags can be supplied by changing *HybRecord.settings['custom_flags']* (page 11). This setting can also be disabled by setting 'allow_undefined_flags' to True[69] in *HybRecord.settings* (page 11).
- **allow_undefined_flags** (bool[70], optional) -- If True[71], allows flags not defined in *ALL_FLAGS* (page 11) or *HybRecord.settings['custom_flags']* (page 11) to be added to the record. If not provided, defaults to the value in *HybRecord.settings['allow_undefined_flags']* (page 11).

Variables

- **id** (*str*[72]) -- Identifier for the hyb record (Hyb format: `<read-num>_<read-count>`)
- **seq** (*str*[73]) -- Nucleotide sequence of the hyb record
- **energy** (*str*[74]) -- Predicted energy of folding
- **seg1_props** (*dict*[75]) -- Information on chimeric segment 1, contains *segment column* (page 11) keys: `ref_name` (`str`[76]), `read_start` (`int`[77]), `read_end` (`int`[78]), `ref_start` (`int`[79]), `ref_end` (`int`[80]), and `score` (`float`[81]).
- **seg2_props** (*dict*[82]) -- Information on segment 2, contains *segment column* (page 11) keys: `ref_name` (`str`[83]), `read_start` (`int`[84]), `read_end` (`int`[85]), `ref_start` (`int`[86]), `ref_end` (`int`[87]), and `score` (`float`[88]).
- **flags** (*dict*[89]) -- Dict of flags with possible *flag keys* (page 11) and values as defined in the *Flags* (page 6) section of the *hybkit Hyb File Specification* (page 5).
- **fold_record** (`FoldRecord` (page 23)) -- Information on the predicted secondary structure of the sequence set by `set_fold_record()` (page 14).
- **allow_undefined_flags** (*bool*[90]) -- Whether to allow undefined flags to be set.

HYBRID_COLUMNS = ('id', 'seq', 'energy')

Record columns 1-3 defining parameters of the overall hybrid, defined by the Hyb format

SEGMENT_COLUMNS = ('ref_name', 'read_start', 'read_end', 'ref_start', 'ref_end', 'score')

Record columns 4-9 and 10-15, respectively, defining annotated parameters of seg1 and seg2 respectively, defined by the Hyb format

ALL_FLAGS = ('count_total', 'count_last_clustering', 'two_way_merged', 'seq_IDs_in_cluster', 'read_count', 'orient', 'det', 'seg1_type', 'seg2_type', 'seg1_det', 'seg2_det', 'miRNA_seg', 'target_reg', 'ext', 'dataset')

Flags defined by the hybkit package. Flags 1-4 are utilized by the Hyb software package. For information on flags, see the *Flags* (page 6) portion of the *hybkit Hyb File Specification* (page 5).

settings = {'allow_undefined_flags': False, 'allow_unknown_seg_types': False, 'custom_flags': [], 'hyb_placeholder': '.', 'mirna_types': ['miRNA', 'microRNA'], 'reorder_flags': True}

Class-level settings. See `settings.HybRecord_settings_info` (page 52) for descriptions.

TypeFinder

Link to `type_finder.TypeFinder` (page 34) class for parsing sequence identifiers in assigning segment types by `eval_types()` (page 14).

SET_PROPS = ('energy', 'full_seg_props', 'fold_record', 'eval_types', 'eval_mirna', 'eval_target')

Properties for the `is_set()` (page 16) method.

- `energy` : energy is not None
- `full_seg_props` : Each seg key is in *segN_props* (page 10) dict and is not None
- `fold_record` : *fold_record* (page 10) has been set
- `eval_types` : *seg1_type* (page 10) and *seg2_type* (page 10) flags have been set
- `eval_mirna` : *miRNA_seg* (page 10) flag has been set

GEN_PROPS = ('has_indels',)

General record properties for the *prop()* (page 16) method.

- has_indels : either seg1 or seg2 alignments has insertions/deletions, shown by differing read/reference length for the same alignment

STR_PROPS = ('id_is', 'id_prefix', 'id_suffix', 'id_contains', 'seq_is', 'seq_prefix', 'seq_suffix', 'seq_contains', 'seg1_is', 'seg1_prefix', 'seg1_suffix', 'seg1_contains', 'seg2_is', 'seg2_prefix', 'seg2_suffix', 'seg2_contains', 'any_seg_is', 'any_seg_prefix', 'any_seg_suffix', 'any_seg_contains', 'seg1_type_is', 'seg1_type_prefix', 'seg1_type_suffix', 'seg1_type_contains', 'seg2_type_is', 'seg2_type_prefix', 'seg2_type_suffix', 'seg2_type_contains', 'any_seg_type_is', 'any_seg_type_prefix', 'any_seg_type_suffix', 'any_seg_type_contains')

String-comparison properties for the *prop()* (page 16) method.

- **Field Types:**
  - id : *record.id* (page 10)
  - seq : *record.seq* (page 10)
  - seg1 : *seg1_props['ref_name']* (page 10)
  - seg2 : *seg2_props['ref_name']* (page 10)
  - any_seg : *seg1_props['ref_name']* (page 10) OR *seg1_props['ref_name']* (page 10)
  - seg1_type : *seg1_type* (page 6) flag
  - seg2_type : *seg2_type* (page 6) flag
  - any_seg_type : *seg1_type* (page 6) OR *seg2_type* (page 6) flags
- **Comparisons:**
  - is : Comparison string matches field exactly
  - prefix : Comparison string matches beginning of field
  - suffix : Comparison string matches end of field
  - contains : Comparison string is contained within field

MIRNA_PROPS = ('has_mirna', 'no_mirna', 'mirna_dimer', 'mirna_not_dimer', '5p_mirna', '3p_mirna')

miRNA-evaluation-related properties for the *prop()* (page 16) method. Requires *miRNA_seg* (page 6) flag to be set by *eval_mirna()* (page 15) method.

- has_mirna : **Either or Both** Seg1 or seg2 hve been **identified as** a miRNA
- no_mirna : **Both** Seg1 and seg2 have been identified as **Not** a miRNA
- mirna_dimer : **Both** seg1 and seg2 have been **identified as** a miRNA
- mirna_not_dimer : **One and Only One** of seg1 or seg2 has been **identified as** a miRNA
- 5p_mirna : Seg1 (5p) has been identified as a miRNA
- 3p_mirna : Seg2 (3p) has been identified as a miRNA

MIRNA_STR_PROPS = ('mirna_is', 'mirna_prefix', 'mirna_suffix', 'mirna_contains', 'target_is', 'target_prefix', 'target_suffix', 'target_contains', 'mirna_seg_type_is', 'mirna_seg_type_prefix', 'mirna_seg_type_suffix', 'mirna_seg_type_contains', 'target_seg_type_is', 'target_seg_type_prefix', 'target_seg_type_suffix', 'target_seg_type_contains')

- Comparisons:
- `is` : Comparison string matches field exactly
- `prefix` : Comparison string matches beginning of field
- `suffix` : Comparison string matches end of field
- `contains` : Comparison string is contained within field

HAS_PROPS = ('has_indels', 'id_is', 'id_prefix', 'id_suffix', 'id_contains', 'seq_is', 'seq_prefix', 'seq_suffix', 'seq_contains', 'seg1_is', 'seg1_prefix', 'seg1_suffix', 'seg1_contains', 'seg2_is', 'seg2_prefix', 'seg2_suffix', 'seg2_contains', 'any_seg_is', 'any_seg_prefix', 'any_seg_suffix', 'any_seg_contains', 'seg1_type_is', 'seg1_type_prefix', 'seg1_type_suffix', 'seg1_type_contains', 'seg2_type_is', 'seg2_type_prefix', 'seg2_type_suffix', 'seg2_type_contains', 'any_seg_type_is', 'any_seg_type_prefix', 'any_seg_type_suffix', 'any_seg_type_contains', 'has_mirna', 'no_mirna', 'mirna_dimer', 'mirna_not_dimer', '5p_mirna', '3p_mirna', 'mirna_is', 'mirna_prefix', 'mirna_suffix', 'mirna_contains', 'target_is', 'target_prefix', 'target_suffix', 'target_contains', 'mirna_seg_type_is', 'mirna_seg_type_prefix', 'mirna_seg_type_suffix', 'mirna_seg_type_contains', 'target_seg_type_is', 'target_seg_type_prefix', 'target_seg_type_suffix', 'target_seg_type_contains')

All allowed properties for the *prop()* (page 16) method. See *GEN_PROPS* (page 12), *STR_PROPS* (page 12), *MIRNA_PROPS* (page 12), and *MIRNA_STR_PROPS* (page 12)

set_flag(*flag_key: str*[91]*, flag_val: Optional*[92]*[Union*[93]*[float*[94]*, int*[95]*, str*[96]*, bool*[97]*]], allow_undefined_flags: Optional*[98]*[bool*[99]*] = None*) → None[100]

Set the value of record `flag_key` to `flag_val`.

Parameters

- **flag_key** (*str*[101]) -- Key for flag to set.
- **flag_val** -- Value for flag to set.
- **allow_undefined_flags** (bool[102], optional) -- Allow inclusion of flags not defined in *ALL_FLAGS* (page 11) or in *settings['custom_flags']* (page 11). If not provided, uses setting in `'HybRecord.allow_undefined_flags'` (Defaults to value in: *settings['allow_undefined_flags']* (page 11)).

get_seg1_type(*require: bool*[103] *= False*) → Optional[104][str[105]]

Return the *seg1_type* (page 6) flag if defined, or return None.

Parameters

require -- If `True`, raise an error if seg1_type is not defined.

get_seg2_type(*require: bool*[106] *= False*) → Optional[107][str[108]]

Return the *seg2_type* (page 6) flag if defined, or return None.

Parameters

require (bool[109], optional) -- If `True`, raise an error if seg2_type is not defined.

get_seg_types(*require: bool*[110] *= False*) → Tuple[111][Optional[112][str[113]], Optional[114][str[115]]]

Return "seg1_type" (or None), "seg2_type" (or None) flags.

Return a tuple of the *seg1_type* (page 6) and *seg2_type* (page 6) flags for each respective flag that is defined, or None for each flag that is not.

Parameters
require (bool[116], optional) -- If True, raise an error if either flag is not defined.

get_read_count(*require: bool*[117] *= False*) → Optional[118][int[119]]

Return the *read_count* (page 6) flag if defined, otherwise return None.

Parameters
require (bool[120], optional) -- If True, raise an error if the "read_count" flag is not defined.

get_record_count(*require: bool*[121] *= False*) → int[122]

Return *count_total* (page 6) flag if defined, or return 1 (this record).

Parameters
require (bool[123], optional) -- If True, raise an error if the "count_total" flag is not defined.

get_mirna_props(*allow_mirna_dimers: bool*[124] *= False*, *require: bool*[125] *= True*) → Optional[126][Dict[127]]

Return the seg_props dict corresponding to the miRNA segment, if set.

If *eval_mirna()* (page 15) has been run, return the seg_props dict corresponding to the miRNA segment type as determined by checking the *miRNA_seg* (page 6) flag, or None[128] if the record does not contain a miRNA.

Parameters

- **allow_mirna_dimers** (bool[129], optional) -- If True, consider miRNA dimers as a miRNA/target pair and return the 5p miRNA segment properties.
- **require** (bool[130], optional) -- If True, raise an error if the read does not contain a miRNA-annotated segment (Default: True).

get_target_props(*allow_mirna_dimers: bool*[131] *= False*, *require: bool*[132] *= True*) → Optional[133][Dict[134]]

Return the seg_props dict corresponding to the target segment, if set.

If *eval_mirna()* (page 15) has been run, return the seg_props dict corresponding to the target segment type as determined by checking the *miRNA_seg* (page 6) flag, (and returning the other segment), or None[135] if the record does not contain a miRNA or contains two miRNAs.

Parameters

- **allow_mirna_dimers** (bool[136], optional) -- If True, consider miRNA dimers as a miRNA/target pair and return the 3p miRNA segment properties as the arbitrarily-selected "target" of the dimer pair.
- **require** (bool[137], optional) -- If True, raise an error if the read does not contain a single target-annotated segment (Default: True).

eval_types(*allow_unknown: Optional*[138]*[bool*[139]*] = None*) → None[140]

Find the types of each segment using the the *TypeFinder* (page 11) class.

This method provides HybRecord.seg1_props and HybRecord.seg2_props to the *TypeFinder* (page 11) class, linked as attribute *HybRecord.TypeFinder* (page 11). This uses the method: *TypeFinder.find* (page 35) set by *TypeFinder.set_method* (page 35) or *TypeFinder.set_custom_method* (page 36) to set the *seg1_type* (page 6), *seg2_type* (page 6) flags if not already set.

To use a type-finding method other than the default, prepare the *TypeFinder* (page 34) class by preparing and setting *TypeFinder.params* (page 34) and using *TypeFinder.set_method* (page 35).

Parameters
allow_unknown (bool[141], optional) -- If True, allow segment types that cannot be identified and set them as "unknown". Otherwise raise an error. If not provided uses setting in *settings['allow_unknown_seg_types']* (page 11).

set_fold_record(*fold_record: Union*[142]*[*FoldRecord *(page 23), Tuple*[143]*[*FoldRecord *(page 23), Any*[144]*]]*, *allow_energy_mismatch: bool*[145] *= False*) → None[146]

Check and set provided fold_record (*FoldRecord* (page 23)) as attribute fold_record.

Ensures that fold_record argument is an instance of FoldRecord and has a matching sequence to this HybRecord, then set as *HybRecord.fold_record* (page 10).

Parameters

- **fold_record** (`FoldRecord` (page 23)) -- *FoldRecord* (page 23) instance to set as *HybRecord.fold_record* (page 10).
- **allow_energy_mismatch** (`bool`[147], optional) -- If `True`, allow mismatched fold_record and HybRecord energy. Otherwise, raise an error.

eval_mirna(*override: bool*[148] *= False*, *mirna_types: Optional*[149]*[bool*[150]*] = None*) → None[151]

Analyze and set miRNA properties from type properties in the hyb record.

If not already done, determine whether a miRNA exists within this record and set the *miRNA_seg* (page 6) flag. This evaluation requires the *seg1_type* (page 6) and *seg2_type* (page 6) flags to be populated, which can be performed by the *eval_types()* (page 14) method.

Parameters

- **override** (`bool`[152], optional) -- If `True`, override existing *miRNA_seg* (page 6) flag if present.
- **mirna_types** (`list`[153], `tuple`[154], or `set`[155], optional) -- Iterable of string representing sequence types considered as miRNA. Otherwise, the types are used from *settings['mirna_types']* (page 11) (it is suggested that this be provided as a `set`[156] for fastest checking).

mirna_details(*detail: Literal*[157]*['all', 'mirna_ref', 'target_ref', 'mirna_seg_type', 'target_seg_type', 'mirna_seq', 'target_seq', 'mirna_fold', 'target_fold'] = 'all'*, *allow_mirna_dimers: bool*[158] *= False*) → Optional[159][Union[160][Dict[161], str[162]]]

Provide a detail about the miRNA or target following *eval_mirna()* (page 15).

Analyze miRNA properties within the sequence record and provide a detail as output. Unless `allow_mirna_dimers` is `True`, this method requires record to contain a non-dimer miRNA, otherwise an error will be raised.

Parameters

- **detail** (*str*[163]) --

  Type of detail to return. Options include:
  `all` : Dict of all properties (default)
  `mirna_ref` : Identifier for Assigned miRNA
  `target_ref` : Identifier for Assigned Target
  `mirna_seg_type` : Assigned seg_type of miRNA
  `target_seg_type` : Assigned seg_type of target
  `mirna_seq` : Annotated subsequence of miRNA
  `target_seq` : Annotated subsequence of target
  `mirna_fold` : Annotated fold substring of miRNA (requires fold_record set)
  `target_fold` : Annotated fold substring of target (requires fold_record set)

- **allow_mirna_dimers** (`bool`[164], optional) -- Allow miRNA/miRNA dimers. The 5p-position will be assigned as the "miRNA", and the 3p-position will be assigned as the "target".

mirna_detail(**args*, ***kwargs*)

Deprecate, alias for `mirna_details()` (page 15).

Deprecated since version v0.3.0.

is_set(*prop: str*[165]) → bool[166]

Return `True` if HybRecord property "prop" is set (if relevant) and is not `None`.

Options described in `SET_PROPS` (page 11).

Parameters
prop (`str`[167]) -- Property / Analysis to check

not_set(*prop: str*[168]) → bool[169]

Return `False` if HybRecord property "prop" is set (if relevant) and is not `None`.

(returns `not is_set(prop)`)

Parameters
prop (`str`[170]) -- Property / Analysis to check

prop(*prop: str*[171], *prop_compare: Optional*[172]*[str*[173]*] = None*) → bool[174]

Return `True` if HybRecord has property: `prop`.

Check property against list of allowed properties in `HAS_PROPS` (page 13). If query property has a string comparator, provide this in prop_compare. Raises an error if a prerequisite field is not set (use `is_set()` (page 16) to check whether properties are set).

Specific properties available to check are described in attributes:

`GEN_PROPS` (page 12)	General Record Properties
`STR_PROPS` (page 12)	Field String Comparison Properties
`MIRNA_PROPS` (page 12)	miRNA-Associated Record Properties
`MIRNA_STR_PROPS` (page 12)	miRNA-Associated String Comparison Properties

Parameters

- **prop** (`str`[175]) -- Property to check
- **prop_compare** (`str`[176], optional) -- Comparator to check.

has_prop(**args*, ***kwargs*)

Return `True` if HybRecord has property: `prop`.

Deprecated since version v0.3.0: Use `prop()` (page 16) instead.

to_line(*newline: bool*[177] *= True*, *sep: str*[178] *= '\t'*) → str[179]

Return a hyb-format string representation of the record.

Parameters

- **newline** (`bool`[180], optional) -- Terminate returned string with a newline (default: `True`)
- **sep** (`str`[181], optional) -- Separator between fields (Default: "\t")

to_csv(*newline: bool*[182] *= False*) → str[183]

Return a comma-separated hyb-format string representation of the record.

Parameters
newline (`bool`[184], optional) -- If `True`, end the returned string with a newline.

to_fields(*missing_obj: Optional[185][Union[186][float[187], int[188], str[189], bool[190]]] = None*) → dict[191]

Return a python dictionary representation of the record.

Returns a dictionary with keys corresponding to the fields in the hyb-format file, and values corresponding to the values in the record. Output can be used with the pandas DataFrame constructor or csv.DictWriter.

Parameters

missing_obj (*optional*) -- Object to use for missing values. Default = None[192].

to_fasta_record(*mode: Literal[193]['hybrid', 'seg1', 'seg2', 'mirna', 'target'] = 'hybrid', annotate: bool[194] = True, allow_mirna_dimers: bool[195] = False*) → None[196]

Return nucleotide sequence as BioPython SeqRecord object.

Parameters

- **mode** (str[197], optional) --

  Determines which sequence component to return. Options:
  `hybrid`: Entire hybrid sequence (default)
  `seg1`: Sequence 1 (if defined)
  `seg2`: Sequence 2 (if defined)
  `miRNA`: miRNA sequence of miRNA/target pair (if defined, else None)
  `target`: Target sequence of miRNA/target pair (if defined, else None)

- **annotate** (bool[198], optional) -- Add name of components to fasta sequence identifier if present.

- **allow_mirna_dimers** (bool[199], optional) --

  If `True`, allow miRNA dimers to be
  returned as miRNA sequence (the 5p segment
  will be selected as the "miRNA").

to_fasta_str(*mode: Literal[200]['hybrid', 'seg1', 'seg2', 'mirna', 'target'] = 'hybrid', annotate: bool[201] = True*) → str[202]

Return nucleotide sequence as a fasta string.

Parameters

- **mode** (str[203], optional) --

  as with *to_fasta_record()* (page 17) method.

- **annotate** (bool[204], optional) -- Add name of components to fasta sequence identifier if present.

classmethod **from_line**(*line: str[205], hybformat_id: bool[206] = False, hybformat_ref: bool[207] = False*) → Self

Construct a HybRecord instance from a single-line hyb-format string.

The Hyb software package ([Travis2014]) records read-count information in the "id" field of the record, which can be read by setting `hybformat_id=True`. Additionally, the Hyb hOH7 database contains the segment type in the identifier of each reference in the 4th field, which can be read by setting `hybformat_ref=True`.

Parameters

- **line** (*str[208]*) -- hyb-format string containing record information.

- **hybformat_id** (bool[209], optional) -- If `True`, read count information from identifier in <read_number>_<read_count> format.

- **hybformat_ref** (bool[210], optional) -- If True, read additional record information from identifier in <gene_id>_<transcript_id>_<gene_name>_<seg_type> format.

Returns
HybRecord (page 9) instance containing record information.

classmethod from_fasta_records(*seg1_record: None[211], seg2_record: None[212], hyb_id: Optional[213][str[214]] = None, energy: Optional[215][Union[216][float[217], int[218], str[219]]] = None, flags: Optional[220][Dict[221][str[222], Any[223]]] = None*) → Self

Construct a HybRecord instance from two BioPython SeqRecord Objects.

Create artificial HybRecord from two SeqRecord Objects For the hybrid:

id: [seg1_record.id]--[seg2_record.id] (overwritten by "id" parameter if provided)
seq: seg1_record.seq + seg2_record

For each segment:

FASTA_Sequence_ID -> segN_ref_name
FASTA_Description -> Flags: segN_det (Overwritten if segN_det flag is provided directly)

Optional fields to add via function arguments:

hyb_id
energy
flags

Parameters

- **seg1_record** (*SeqRecord*) -- Biopython SeqRecord object containing information on the left/first/5p hybrid segment (seg1)
- **seg2_record** (*SeqRecord*) -- Biopython SeqRecord object containing information on the right/second/3p hybrid segment (seg2)
- **hyb_id** (str[224], optional) -- Identifier for the hyb record (overwrites generated id if provided)
- **energy** (str[225] or float[226], optional) -- Predicted energy of sequence folding in kcal/mol
- **flags** (dict[227], optional) -- Dict with keys of flags for the record and their associated values. Any flags provided overwrite default-generated flags.

Returns
HybRecord (page 9) instance containing record information.

classmethod to_fields_header() → Literal[228]['id', 'seq', 'energy', 'seg1_ref_name', 'seg1_read_start', 'seg1_read_end', 'seg1_ref_start', 'seg1_ref_end', 'seg1_score', 'seg2_ref_name', 'seg2_read_start', 'seg2_read_end', 'seg2_ref_start', 'seg2_ref_end', 'seg2_score', 'flags']

Return a list of the fields in a *HybRecord* (page 9) object.

For use with the *to_fields()* (page 16) method.

classmethod to_csv_header(*newline: bool[229] = False*) → Literal[230]['id,seq,energy,seg1_ref_name,seg1_read_start,seg1_read_end,seg1_ref_start,seg1_ref_e

Return a comma-separated string representation of the fields in the record.

For use with the *to_csv()* (page 16) method.

Parameters

newline (bool[231], optional) -- If True, end the returned string with a newline.

[33] https://docs.python.org/3/library/stdtypes.html#str
[34] https://docs.python.org/3/library/stdtypes.html#str
[35] https://docs.python.org/3/library/typing.html#typing.Optional
[36] https://docs.python.org/3/library/typing.html#typing.Union
[37] https://docs.python.org/3/library/functions.html#float
[38] https://docs.python.org/3/library/functions.html#int
[39] https://docs.python.org/3/library/stdtypes.html#str
[40] https://docs.python.org/3/library/typing.html#typing.Optional
[41] https://docs.python.org/3/library/typing.html#typing.Dict
[42] https://docs.python.org/3/library/stdtypes.html#str
[43] https://docs.python.org/3/library/typing.html#typing.Union
[44] https://docs.python.org/3/library/functions.html#float
[45] https://docs.python.org/3/library/functions.html#int
[46] https://docs.python.org/3/library/stdtypes.html#str
[47] https://docs.python.org/3/library/typing.html#typing.Optional
[48] https://docs.python.org/3/library/typing.html#typing.Dict
[49] https://docs.python.org/3/library/stdtypes.html#str
[50] https://docs.python.org/3/library/typing.html#typing.Union
[51] https://docs.python.org/3/library/functions.html#float
[52] https://docs.python.org/3/library/functions.html#int
[53] https://docs.python.org/3/library/stdtypes.html#str
[54] https://docs.python.org/3/library/typing.html#typing.Optional
[55] https://docs.python.org/3/library/typing.html#typing.Dict
[56] https://docs.python.org/3/library/stdtypes.html#str
[57] https://docs.python.org/3/library/typing.html#typing.Any
[58] https://docs.python.org/3/library/typing.html#typing.Optional
[59] https://docs.python.org/3/library/functions.html#int
[60] https://docs.python.org/3/library/typing.html#typing.Optional
[61] https://docs.python.org/3/library/functions.html#bool
[62] https://docs.python.org/3/library/stdtypes.html#str
[63] https://docs.python.org/3/library/stdtypes.html#str
[64] https://docs.python.org/3/library/stdtypes.html#str
[65] https://docs.python.org/3/library/functions.html#float
[66] https://docs.python.org/3/library/stdtypes.html#dict
[67] https://docs.python.org/3/library/stdtypes.html#dict
[68] https://docs.python.org/3/library/stdtypes.html#dict
[69] https://docs.python.org/3/library/constants.html#True
[70] https://docs.python.org/3/library/functions.html#bool
[71] https://docs.python.org/3/library/constants.html#True
[72] https://docs.python.org/3/library/stdtypes.html#str
[73] https://docs.python.org/3/library/stdtypes.html#str
[74] https://docs.python.org/3/library/stdtypes.html#str
[75] https://docs.python.org/3/library/stdtypes.html#dict
[76] https://docs.python.org/3/library/stdtypes.html#str
[77] https://docs.python.org/3/library/functions.html#int
[78] https://docs.python.org/3/library/functions.html#int
[79] https://docs.python.org/3/library/functions.html#int
[80] https://docs.python.org/3/library/functions.html#int
[81] https://docs.python.org/3/library/functions.html#float
[82] https://docs.python.org/3/library/stdtypes.html#dict
[83] https://docs.python.org/3/library/stdtypes.html#str
[84] https://docs.python.org/3/library/functions.html#int
[85] https://docs.python.org/3/library/functions.html#int
[86] https://docs.python.org/3/library/functions.html#int
[87] https://docs.python.org/3/library/functions.html#int
[88] https://docs.python.org/3/library/functions.html#float
[89] https://docs.python.org/3/library/stdtypes.html#dict
[90] https://docs.python.org/3/library/functions.html#bool
[91] https://docs.python.org/3/library/stdtypes.html#str
[92] https://docs.python.org/3/library/typing.html#typing.Optional
[93] https://docs.python.org/3/library/typing.html#typing.Union
[94] https://docs.python.org/3/library/functions.html#float
[95] https://docs.python.org/3/library/functions.html#int
[96] https://docs.python.org/3/library/stdtypes.html#str
[97] https://docs.python.org/3/library/functions.html#bool
[98] https://docs.python.org/3/library/typing.html#typing.Optional
[99] https://docs.python.org/3/library/functions.html#bool
[100] https://docs.python.org/3/library/constants.html#None
[101] https://docs.python.org/3/library/stdtypes.html#str
[102] https://docs.python.org/3/library/functions.html#bool
[103] https://docs.python.org/3/library/functions.html#bool
[104] https://docs.python.org/3/library/typing.html#typing.Optional
[105] https://docs.python.org/3/library/stdtypes.html#str
[106] https://docs.python.org/3/library/functions.html#bool

2.1.2 HybFile Class

class hybkit.**HybFile**(*path: str[232], *args: Any[233], hybformat_id: Optional[234][bool[235]] = None, hybformat_ref: Optional[236][bool[237]] = None, from_file_like: bool[238] = False, **kwargs: Any[239]*)

Wrapper for a hyb-format text file which returns entries (lines) as HybRecord objects.

Parameters

- **path** (*str*[240]) -- Path to text file to open as hyb-format file.
- ***args** -- Arguments passed to *open()* (page 22) function to open a text file for reading/writing.
- **hybformat_id** (bool[241], optional) -- If True, during parsing of lines read count information from identifier in <read_number>_<read_count> format. Defaults to value in *settings['hybformat_id']* (page 21).
- **hybformat_ref** (bool[242], optional) -- If True, during parsing of lines read additional record information from identifier in <gene_id>_<transcript_id>_<gene_name>_<seg_type> format. Defaults to value in *settings['hybformat_ref']* (page 21).
- **from_file_like** (bool[243], optional) -- If True, the first argument is treated as a file-like object (such as io.StringIO or gzip.GzipFile) and the remaining positional arguments are ignored. (Default False``)
- ****kwargs** -- Keyword arguments passed to *open()* (page 22) function to open a text file for reading/writing.

Variables

- **hybformat_id** (*bool*[244]) -- Read count information from identifier during line parsing
- **hybformat_ref** (*bool*[245]) -- Read type information from reference name during line parsing
- **fh** (*file*) -- Underlying file handle for the HybFile object.

settings = {'hybformat_id': False, 'hybformat_ref': False}

Class-level settings. See *hybkit.settings.HybFile_settings_info* (page 52) for descriptions.

close() → None[246]

Close the file.

read_record() → str[247]

Return next line of hyb file as HybRecord object.

read_records() → List[248][str[249]]

Return list of all (remaining) records in hyb file as HybRecord objects.

write_record(*write_record: HybRecord (page 9)*) → None[250]

Write a HybRecord object to file as a Hyb-format string.

Unlike the file.write() method, this method will add a newline to the end of each written record line.

Parameters

write_record (HybRecord (page 9)) -- Record to write.

write_records(*write_records: Iterable[251][HybRecord (page 9)]*) → None[252]

Write a sequence of HybRecord objects as hyb-format lines to the Hyb file.

Unlike the file.writelines() method, this method will add a newline to the end of each written record line.

Parameters
write_records (*list*[253]) -- List of *HybRecord* (page 9) objects to write.

write_fh(**args*, ***kwargs*) → None[254]

Write directly to the underlying file handle.

write(**_args*, ***_kwargs*) → None[255]

Implement no-op / error for "write" method to catch errors.

Use *write_record()* (page 21) or *write_fh()* (page 22) instead.

classmethod open(*path: str*[256], **args: Any*[257], *hybformat_id: Optional*[258]*[bool*[259]*] = None*, *hybformat_ref: Optional*[260]*[bool*[261]*] = None*, ***kwargs: Any*[262]) → Self

Open a path to a text file using *open()* (page 22) and return a HybFile object.

Arguments match those of the Python3 built-in *open()* (page 22) function and are passed directly to it.

This method is provided as a convenience function for drop-in replacement of the built-in *open()* (page 22) function.

Specific keyword arguments are provided for HybFile-specific settings:

Parameters

- **path** (*str*[263]) -- Path to file to open.
- **hybformat_id** (bool[264], optional) -- If True, during parsing of lines read count information from identifier in <read_number>_<read_count> format. Defaults to value in *settings['hybformat_id']* (page 21).
- **hybformat_ref** (bool[265], optional) -- If True, during parsing of lines read additional record information from identifier in <gene_id>_<transcript_id>_<gene_name>_<seg_type> format. Defaults to value in *settings['hybformat_ref']* (page 21).

Example usage:

```
with HybFile.open('path/to/file.hyb', 'r') as hyb_file:
    for record in hyb_file:
        print(record)
```

Parameters

- ***args** -- Passed directly to *open()* (page 22).
- ****kwargs** -- Passed directly to *open()* (page 22).

Returns
HybFile (page 21) object.

2.1.3 FoldRecord Class

class hybkit.FoldRecord(*id: str*[266], *seq: str*[267], *fold: str*[268], *energy: Optional*[269]*[Union*[270]*[float*[271], *int*[272], *str*[273]*]] = None*, *seq_type: Optional*[274]*[Literal*[275]*['static', 'dynamic']] = None*)

Class for storing secondary structure (folding) information for a nucleotide sequence.

This class supports the following file types: (Data courtesy of [Gay2018])

- The ".vienna" file format used by the ViennaRNA[276] package ([ViennaFormat]; [Lorenz2011]):

  Example:

```
34_151138_MIMAT0000076_MirBase_miR-21_microRNA_1_19-...
TAGCTTATCAGACTGATGTTAGCTTATCAGACTGATG
.....((((((.((((((......)))))).))))))   (-11.1)
```

- The ".ct" file format used by UNAFold[277] and other packages ([CTFormat], [Zuker2003]):

  Example:

```
41          dG = -8 dH = -93.9        seq1_name-seq2_name
1 A         0        2        0        1        0        0
2 G         1        3        0        2        0        0
...
```

(continues on next page)

232 https://docs.python.org/3/library/stdtypes.html#str
233 https://docs.python.org/3/library/typing.html#typing.Any
234 https://docs.python.org/3/library/typing.html#typing.Optional
235 https://docs.python.org/3/library/functions.html#bool
236 https://docs.python.org/3/library/typing.html#typing.Optional
237 https://docs.python.org/3/library/functions.html#bool
238 https://docs.python.org/3/library/functions.html#bool
239 https://docs.python.org/3/library/typing.html#typing.Any
240 https://docs.python.org/3/library/stdtypes.html#str
241 https://docs.python.org/3/library/functions.html#bool
242 https://docs.python.org/3/library/functions.html#bool
243 https://docs.python.org/3/library/functions.html#bool
244 https://docs.python.org/3/library/functions.html#bool
245 https://docs.python.org/3/library/functions.html#bool
246 https://docs.python.org/3/library/constants.html#None
247 https://docs.python.org/3/library/stdtypes.html#str
248 https://docs.python.org/3/library/typing.html#typing.List
249 https://docs.python.org/3/library/stdtypes.html#str
250 https://docs.python.org/3/library/constants.html#None
251 https://docs.python.org/3/library/typing.html#typing.Iterable
252 https://docs.python.org/3/library/constants.html#None
253 https://docs.python.org/3/library/stdtypes.html#list
254 https://docs.python.org/3/library/constants.html#None
255 https://docs.python.org/3/library/constants.html#None
256 https://docs.python.org/3/library/stdtypes.html#str
257 https://docs.python.org/3/library/typing.html#typing.Any
258 https://docs.python.org/3/library/typing.html#typing.Optional
259 https://docs.python.org/3/library/functions.html#bool
260 https://docs.python.org/3/library/typing.html#typing.Optional
261 https://docs.python.org/3/library/functions.html#bool
262 https://docs.python.org/3/library/typing.html#typing.Any
263 https://docs.python.org/3/library/stdtypes.html#str
264 https://docs.python.org/3/library/functions.html#bool
265 https://docs.python.org/3/library/functions.html#bool

(continued from previous page)

```
...
...
40        G       39      41      11      17      39      41
41        T       40      0       10      18      40      0
```

A minimum amount of data necessary for a FoldRecord object is a sequence identifier, a genomic sequence, and its fold representation.

Two types of FoldRecord objects are supported, 'static' and 'dynamic'. Static FoldRecord objects are those where the 'seq' attribute matches exactly to the corresponding `HybRecord.seq` attribute (where applicable). Dynamic FoldRecord objects are those where `FoldRecord.seq` is reconstructed from aligned regions of a `HybRecord.seq` chimeric read: Longer for chimeras with overlapping alignments, shorter for chimeras with gapped alignments.

Overlapping Alignment Example:

```
Static:
seg1: 1111111111111111111111
seg2:                   222222222222222222222
seq:  TAGCTTATCAGACTGATGTTTTAGCTTATCAGACTGATG

Dynamic:
seg1: 1111111111111111111111
seg2:                       222222222222222222222
seq:  TAGCTTATCAGACTGATGTTTTTTTTAGCTTATCAGACTGATG
```

Gapped Alignment Example:

```
Static:
seg1:   1111111111111111
seg2:                     222222222222222222
seq:  TTAGCTTATCAGACTGATGTTAGCTTATCAGACTGATG

Dynamic:
seg1: 1111111111111111
seg2:                 222222222222222222
seq:  AGCTTATCAGACTGATTAGCTTATCAGACTGATG
```

Dynamic sequences are found in the Hyb program *_hybrids_ua.hyb file type. This is primarily relevant in error-checking when setting the *HybRecord.set_fold_record()* (page 14) method.

When the 'static' FoldRecord type is used, the following methods are used for `HybRecord.fold_record` error-checking:

- *static_count_hyb_record_mismatches()* (page 25)

When the 'dynamic' FoldRecord type is used, the following methods are used for `HybRecord.fold_record` error-checking:

- *dynamic_count_hyb_record_mismatches()* (page 25)

Parameters

- **id** (*str*[278]) -- Identifier for record
- **seq** (*str*[279]) -- Nucleotide sequence of record.

- **fold** (*str*[280]) -- Fold representation of record.
- **energy** (str[281] or float[282], optional) -- Energy of folding for record.
- **seq_type** (str[283], optional) -- Expect sequence to be 'static' (match exactly to corresponding *HybRecord.seq* (page 10)), or 'dynamic' (construct from pieces of *HybRecord.seq* (page 10)). if not provided, defaults to `~settings['seq_type']` (page 25) setting. See `hybkit.settings.FoldRecord_settings_info` (page 53) for descriptions.

Variables

- **id** (*str*[284]) -- Sequence Identifier (often seg1name-seg2name)
- **seq** (*str*[285]) -- Genomic Sequence
- **fold** (*str*[286]) -- Dot-bracket Fold Representation, '(', '.', and ')' characters
- **energy** (*str*[287]) -- Predicted energy of folding
- **seq_type** (*str*[288]) -- Whether sequence is 'static' or 'dynamic' (Default: 'static'; see Args for details)

settings = {'allowed_mismatches': 0, 'error_mode': 'raise', 'fold_placeholder': '.', 'seq_type': 'static'}

Class-level settings. See `hybkit.settings.FoldRecord_settings_info` (page 53) for descriptions.

to_vienna_lines(*newline: bool*[289] *= True*) → List[290][str[291]]

Return a list of lines for the record in vienna format.

See (*Vienna File Format* (page 23)).

Parameters
newline (bool[292], optional) -- Add newline character to the end of each returned line. (Default: True)

to_vienna_string(*newline: bool*[293] *= True*) → str[294]

Return a 3-line string for the record in vienna format.

See (*Vienna File Format* (page 23)).

Parameters
newline (bool[295], optional) -- Terminate the returned string with a newline character. (Default: True)

count_hyb_record_mismatches(*hyb_record:* HybRecord *(page 9)*) → int[296]

Count mismatches between `hyb_record.seq` and `fold_record.seq`.

Uses `static_count_hyb_record_mismatches()` (page 25) if `seq_type` is `static`, or `dynamic_count_hyb_record_mismatches()` (page 25) if `seq_type` is `dynamic`.

Parameters
hyb_record (HybRecord (page 9)) -- hyb_record for comparison.

static_count_hyb_record_mismatches(*hyb_record:* HybRecord *(page 9)*) → int[297]

Count mismatches between `hyb_record.seq` and `fold_record.seq`.

Parameters
hyb_record (HybRecord (page 9)) -- hyb_record for comparison.

dynamic_count_hyb_record_mismatches(*hyb_record: HybRecord (page 9)*) → int[298]

Count mismatches between hyb_record.seq and dynamic fold_record.seq.

Parameters

hyb_record (HybRecord (page 9)) -- hyb_record for comparison

matches_hyb_record(*hyb_record: HybRecord (page 9), allowed_mismatches: Optional[299][int[300]] = None*) → bool[301]

Return `True` if self.seq and hyb_record.seq mismatches are <= allowed_mismatches.

Parameters

- **hyb_record** (HybRecord (page 9)) -- hyb_record to compare.
- **allowed_mismatches** (int[302], optional) -- Number of mismatches allowed for a match. If not provided, defaults to the option in *settings['allowed_mismatches']* (page 25).

ensure_matches_hyb_record(*hyb_record: HybRecord (page 9), allowed_mismatches: Optional[303][int[304]] = None*) → None[305]

Ensure self.seq matches hyb_record.seq, else raise an error.

Parameters

- **hyb_record** (HybRecord (page 9)) -- hyb_record to compare.
- **allowed_mismatches** (int[306], optional) -- Number of mismatches allowed for a match. If not provided, defaults to the option in *settings['allowed_mismatches']* (page 25).

classmethod from_vienna_lines(*record_lines: List[307][str[308]], error_mode: Optional[309][Literal[310]['raise', 'warn_return', 'return']] = None, seq_type: Optional[311][Literal[312]['static', 'dynamic']] = None*) → Union[313][Tuple[314][None[315], str[316]], Tuple[317][Literal[318]['NOFOLD'], str[319]], Tuple[320][Literal[321]['NOENERGY'], str[322]], Self]

Construct instance from a list of 3 strings of vienna-format ([ViennaFormat]) lines.

See *Vienna File Format* (page 23) for more details.

Parameters

record_lines (list[323] or tuple[324]) -- Iterable of 3 strings corresponding to lines of a vienna-format record.

classmethod from_vienna_string(*record_string: str[325], error_mode: Optional[326][Literal[327]['raise', 'warn_return', 'return']] = None, seq_type: Optional[328][Literal[329]['static', 'dynamic']] = None*) → Union[330][Tuple[331][None[332], str[333]], Tuple[334][Literal[335]['NOFOLD'], str[336]], Tuple[337][Literal[338]['NOENERGY'], str[339]], Self]

Construct instance from a string representing 3 vienna-format ([ViennaFormat]) lines.

See *Vienna File Format* (page 23) for more details.

Parameters

record_string (*str[340] or tuple[341]*) -- 3-line string containing a vienna-format record

classmethod from_ct_lines(*record_lines: List[342][str[343]], error_mode: Optional[344][Literal[345]['raise', 'warn_return', 'return']] = None, seq_type: Optional[346][Literal[347]['static', 'dynamic']] = None*) → Union[348][Tuple[349][None[350], str[351]], Tuple[352][Literal[353]['NOFOLD'], str[354]], Tuple[355][Literal[356]['NOENERGY'], str[357]], Self]

Create a FoldRecord from a list of record lines in ".ct" format ([CTFormat]).

See *CT File Format* (page 23) for more details.

> **Warning:** This method is in beta stage, and is not well-tested.

Parameters
`record_lines` (*list*[358] *or* *tuple*[359]) -- list containing lines of ct record

`classmethod from_ct_string`(*record_string: str*[360], *error_mode: Optional*[361]*[Literal*[362]*['raise', 'warn_return', 'return']] = None*, *seq_type: Optional*[363]*[Literal*[364]*['static', 'dynamic']] = None*) → Union[365][Tuple[366][None[367], str[368]], Tuple[369][Literal[370]['NOFOLD'], str[371]], Tuple[372][Literal[373]['NOENERGY'], str[374]], Self]

Create a FoldRecord entry from a multi-line string from ".ct" format ([CTFormat]).

See *CT File Format* (page 23) for more details.

> **Warning:** This method is in beta stage, and is not well-tested.

Parameters
`record_string` (*str*[375]) -- String containing lines of ct record

[266] https://docs.python.org/3/library/stdtypes.html#str
[267] https://docs.python.org/3/library/stdtypes.html#str
[268] https://docs.python.org/3/library/stdtypes.html#str
[269] https://docs.python.org/3/library/typing.html#typing.Optional
[270] https://docs.python.org/3/library/typing.html#typing.Union
[271] https://docs.python.org/3/library/functions.html#float
[272] https://docs.python.org/3/library/functions.html#int
[273] https://docs.python.org/3/library/stdtypes.html#str
[274] https://docs.python.org/3/library/typing.html#typing.Optional
[275] https://docs.python.org/3/library/typing.html#typing.Literal
[276] https://www.tbi.univie.ac.at/RNA/
[277] http://www.unafold.org/
[278] https://docs.python.org/3/library/stdtypes.html#str
[279] https://docs.python.org/3/library/stdtypes.html#str
[280] https://docs.python.org/3/library/stdtypes.html#str
[281] https://docs.python.org/3/library/stdtypes.html#str
[282] https://docs.python.org/3/library/functions.html#float
[283] https://docs.python.org/3/library/stdtypes.html#str
[284] https://docs.python.org/3/library/stdtypes.html#str
[285] https://docs.python.org/3/library/stdtypes.html#str
[286] https://docs.python.org/3/library/stdtypes.html#str
[287] https://docs.python.org/3/library/stdtypes.html#str
[288] https://docs.python.org/3/library/stdtypes.html#str
[289] https://docs.python.org/3/library/functions.html#bool
[290] https://docs.python.org/3/library/typing.html#typing.List
[291] https://docs.python.org/3/library/stdtypes.html#str
[292] https://docs.python.org/3/library/functions.html#bool
[293] https://docs.python.org/3/library/functions.html#bool
[294] https://docs.python.org/3/library/stdtypes.html#str
[295] https://docs.python.org/3/library/functions.html#bool
[296] https://docs.python.org/3/library/functions.html#int
[297] https://docs.python.org/3/library/functions.html#int
[298] https://docs.python.org/3/library/functions.html#int
[299] https://docs.python.org/3/library/typing.html#typing.Optional
[300] https://docs.python.org/3/library/functions.html#int
[301] https://docs.python.org/3/library/functions.html#bool
[302] https://docs.python.org/3/library/functions.html#int
[303] https://docs.python.org/3/library/typing.html#typing.Optional
[304] https://docs.python.org/3/library/functions.html#int
[305] https://docs.python.org/3/library/constants.html#None
[306] https://docs.python.org/3/library/functions.html#int
[307] https://docs.python.org/3/library/typing.html#typing.List
[308] https://docs.python.org/3/library/stdtypes.html#str
[309] https://docs.python.org/3/library/typing.html#typing.Optional
[310] https://docs.python.org/3/library/typing.html#typing.Literal
[311] https://docs.python.org/3/library/typing.html#typing.Optional
[312] https://docs.python.org/3/library/typing.html#typing.Literal
[313] https://docs.python.org/3/library/typing.html#typing.Union
[314] https://docs.python.org/3/library/typing.html#typing.Tuple
[315] https://docs.python.org/3/library/constants.html#None
[316] https://docs.python.org/3/library/stdtypes.html#str
[317] https://docs.python.org/3/library/typing.html#typing.Tuple
[318] https://docs.python.org/3/library/typing.html#typing.Literal
[319] https://docs.python.org/3/library/stdtypes.html#str
[320] https://docs.python.org/3/library/typing.html#typing.Tuple
[321] https://docs.python.org/3/library/typing.html#typing.Literal
[322] https://docs.python.org/3/library/stdtypes.html#str
[323] https://docs.python.org/3/library/stdtypes.html#list
[324] https://docs.python.org/3/library/stdtypes.html#tuple
[325] https://docs.python.org/3/library/stdtypes.html#str
[326] https://docs.python.org/3/library/typing.html#typing.Optional
[327] https://docs.python.org/3/library/typing.html#typing.Literal
[328] https://docs.python.org/3/library/typing.html#typing.Optional
[329] https://docs.python.org/3/library/typing.html#typing.Literal
[330] https://docs.python.org/3/library/typing.html#typing.Union
[331] https://docs.python.org/3/library/typing.html#typing.Tuple
[332] https://docs.python.org/3/library/constants.html#None
[333] https://docs.python.org/3/library/stdtypes.html#str
[334] https://docs.python.org/3/library/typing.html#typing.Tuple
[335] https://docs.python.org/3/library/typing.html#typing.Literal
[336] https://docs.python.org/3/library/stdtypes.html#str
[337] https://docs.python.org/3/library/typing.html#typing.Tuple
[338] https://docs.python.org/3/library/typing.html#typing.Literal
[339] https://docs.python.org/3/library/stdtypes.html#str

2.1.4 ViennaFile Class

class hybkit.**ViennaFile**(**args: Any[376], seq_type: Optional[377][Literal[378]['static', 'dynamic']] = None, error_mode: Optional[379][Literal[380]['raise', 'warn_return', 'return']] = None, from_file_like: bool[381] = False, **kwargs: Any[382]*)

Vienna file wrapper that returns vienna-format file lines as FoldRecord objects.

See *Vienna File Format* (page 23) for more information.

Parameters

- **seq_type** (str[383], optional) -- Type of FoldRecord to return: `static`, or `dynamic` (if not provided, uses `FoldRecord.settings['seq_type']` (page 25)).
- **error_mode** (str[384], optional) -- String representing the error mode. If None, defaults to the value set in `settings['error_mode']` (page 25). Options: "raise": Raise an error when encountered and exit program; "warn_return": Print a warning and return the error_value; "return": Return the error value with no warnings.
- **from_file_like** (bool[385], optional) -- If True, treat the first argument as a file-like object (such as io.StringIO or gzip.GzipFile) and the remaining positional arguments are ignored (Default `False`).
- ***args** -- Passed to `open()` (page 29).
- ****kwargs** -- Passed to `open()` (page 29).

Variables

- **fh** (`file`) -- File handle for the file being wrapped.
- **foldrecord_seq_type** (*str*[386]) -- Type of FoldRecord to return (see Args)
- **error_mode** (*str*[387]) -- Mode for error catching (see Args)

> **Warning:** Occasionally fold files can be poorly-formatted. In that case, this iterator attempts error-catching but this is not always successful so verbose error modes are encouraged.

read_record(*override_error_mode: Optional[388][Literal[389]['raise', 'warn_return', 'return']] = None*) → Union[390][Tuple[391][None[392], str[393]], Tuple[394][Literal[395]['NOFOLD'], str[396]], Tuple[397][Literal[398]['NOENERGY'], str[399]], Self]

Read next three lines and return output as FoldRecord object.

Parameters
override_error_mode (*str*[400]) -- Override the error_mode set in the `ViennaFile` (page 29) object. See the *ViennaFile Constructor* (page 29) for more information on allowed error modes.

close() → None[401]

Close the file handle.

classmethod open(*path: str[402], *args: Any[403], **kwargs: Any[404]*) → Self

Open a path to a text file using `open()` (page 29) and return relevant file object.

Arguments match those of the Python3 built-in `open()` (page 29) function and are passed directly to it.

This method is provided as a convenience function for drop-in replacement of the built-in `open()` (page 29) function.

Specific keyword arguments are provided for fold-file-specific settings:

Parameters

- **path** (*str*[405]) -- Path to file to open.
- **seq_type** (str[406], optional) -- Type of FoldRecord to return: `static`, or `dynamic` (if not provided, uses *FoldRecord.settings['seq_type']* (page 25)).
- **error_mode** (str[407], optional) -- String representing the error mode. If None, defaults to the value set in *settings['error_mode']* (page 25). Options: "raise": Raise an error when encountered and exit program; "warn_return": Print a warning and return the error_value; "return": Return the error value with no warnings.
- ***args** -- Passed directly to *open()* (page 29).
- ****kwargs** -- Passed directly to *open()* (page 29).

Returns

HybFile (page 21) object.

read_records() → List[408][*FoldRecord* (page 23)]

Return list of all *FoldRecord* (page 23) objects for this file type.

settings = {}

Class-level settings. See *hybkit.settings.FoldFile_settings_info* (page 54) for descriptions.

write_fh(**args: Any*[409], ***kwargs: Any*[410]) → None[411]

Write directly to the underlying file handle.

write_record(*write_record:* FoldRecord *(page 23)*) → None[412]

Write a FoldRecord object for this file type.

Unlike the file.write() method, this method will add a newline to the end of each written record line.

Parameters

write_record (*FoldRecord* (page 23)) -- *FoldRecord* (page 23) objects to write.

write_records(*write_records: Iterable*[413]*[*FoldRecord *(page 23)]*) → None[414]

Write a sequence of FoldRecord objects for this file type.

Unlike the file.writelines() method, this method will add a newline to the end of each written record line.

Parameters

write_records (*list*[415]) -- List of *FoldRecord* (page 23) objects to write.

2.1.5 CtFile Class

class hybkit.**CtFile**(**args: Any[416], seq_type: Optional[417][Literal[418]['static', 'dynamic']] = None, error_mode: Optional[419][Literal[420]['raise', 'warn_return', 'return']] = None, from_file_like: bool[421] = False, **kwargs: Any[422]*)

Ct file wrapper that returns ".ct" file lines as FoldRecord objects.

See *CT File Format* (page 23) for more information.

> **Warning:** This class is in beta stage, and is not well-tested.

Parameters

- **seq_type** (str[423], optional) -- Type of FoldRecord to return: `static`, or `dynamic` (if not provided, uses `FoldRecord.settings['seq_type']` (page 25)).
- **error_mode** (str[424], optional) -- String representing the error mode. If None, defaults to the value set in `settings['error_mode']` (page 25). Options: "raise": Raise an error when encountered and exit program; "warn_return": Print a warning and return the error_value; "return": Return the error value with no warnings.

376 https://docs.python.org/3/library/typing.html#typing.Any
377 https://docs.python.org/3/library/typing.html#typing.Optional
378 https://docs.python.org/3/library/typing.html#typing.Literal
379 https://docs.python.org/3/library/typing.html#typing.Optional
380 https://docs.python.org/3/library/typing.html#typing.Literal
381 https://docs.python.org/3/library/functions.html#bool
382 https://docs.python.org/3/library/typing.html#typing.Any
383 https://docs.python.org/3/library/stdtypes.html#str
384 https://docs.python.org/3/library/stdtypes.html#str
385 https://docs.python.org/3/library/functions.html#bool
386 https://docs.python.org/3/library/stdtypes.html#str
387 https://docs.python.org/3/library/stdtypes.html#str
388 https://docs.python.org/3/library/typing.html#typing.Optional
389 https://docs.python.org/3/library/typing.html#typing.Literal
390 https://docs.python.org/3/library/typing.html#typing.Union
391 https://docs.python.org/3/library/typing.html#typing.Tuple
392 https://docs.python.org/3/library/constants.html#None
393 https://docs.python.org/3/library/stdtypes.html#str
394 https://docs.python.org/3/library/typing.html#typing.Tuple
395 https://docs.python.org/3/library/typing.html#typing.Literal
396 https://docs.python.org/3/library/stdtypes.html#str
397 https://docs.python.org/3/library/typing.html#typing.Tuple
398 https://docs.python.org/3/library/typing.html#typing.Literal
399 https://docs.python.org/3/library/stdtypes.html#str
400 https://docs.python.org/3/library/stdtypes.html#str
401 https://docs.python.org/3/library/constants.html#None
402 https://docs.python.org/3/library/stdtypes.html#str
403 https://docs.python.org/3/library/typing.html#typing.Any
404 https://docs.python.org/3/library/typing.html#typing.Any
405 https://docs.python.org/3/library/stdtypes.html#str
406 https://docs.python.org/3/library/stdtypes.html#str
407 https://docs.python.org/3/library/stdtypes.html#str
408 https://docs.python.org/3/library/typing.html#typing.List
409 https://docs.python.org/3/library/typing.html#typing.Any
410 https://docs.python.org/3/library/typing.html#typing.Any
411 https://docs.python.org/3/library/constants.html#None
412 https://docs.python.org/3/library/constants.html#None
413 https://docs.python.org/3/library/typing.html#typing.Iterable
414 https://docs.python.org/3/library/constants.html#None
415 https://docs.python.org/3/library/stdtypes.html#list

- **from_file_like** (bool[425], optional) -- If True, treat the first argument as a file-like object (such as io.StringIO or gzip.GzipFile) and the remaining positional arguments are ignored (Default `False`).
- ***args** -- Passed to *open()* (page 32).
- ****kwargs** -- Passed to *open()* (page 32).

Variables

- **fh** (`file`) -- File handle for the file being wrapped.
- **foldrecord_seq_type** (*str*[426]) -- Type of FoldRecord to return (see Args)
- **error_mode** (*str*[427]) -- Mode for error catching (see Args)

> **Warning:** Occasionally fold files can be poorly-formatted. In that case, this iterator attempts error-catching but this is not always successful so verbose error modes are encouraged.

read_record() → Union[428][Tuple[429][None[430], str[431]], Tuple[432][Literal[433]['NOFOLD'], str[434]], Tuple[435][Literal[436]['NOENERGY'], str[437]], Self]

Return the next CT record as a *FoldRecord* (page 23) object.

Call next(self.fh) to return the first line of the next entry. Determine the expected number of following lines in the entry, and read that number of lines further. Return lines as a FoldRecord object.

write_record = None

CtFile Record Writing Not Implemented

write_records = None

CtFile Record Writing Not Implemented

close() → None[438]

Close the file handle.

classmethod open(*path: str*[439], **args: Any*[440], ***kwargs: Any*[441]) → Self

Open a path to a text file using *open()* (page 32) and return relevant file object.

Arguments match those of the Python3 built-in *open()* (page 32) function and are passed directly to it.

This method is provided as a convenience function for drop-in replacement of the built-in *open()* (page 32) function.

Specific keyword arguments are provided for fold-file-specific settings:

Parameters

- **path** (*str*[442]) -- Path to file to open.
- **seq_type** (str[443], optional) -- Type of FoldRecord to return: `static`, or `dynamic` (if not provided, uses *FoldRecord.settings['seq_type']* (page 25)).
- **error_mode** (str[444], optional) -- String representing the error mode. If None, defaults to the value set in *settings['error_mode']* (page 25). Options: "raise": Raise an error when encountered and exit program; "warn_return": Print a warning and return the error_value; "return": Return the error value with no warnings.
- ***args** -- Passed directly to *open()* (page 32).
- ****kwargs** -- Passed directly to *open()* (page 32).

Returns

HybFile (page 21) object.

read_records() → List[445][*FoldRecord* (page 23)]

Return list of all `FoldRecord` (page 23) objects for this file type.

settings = {}

Class-level settings. See *hybkit.settings.FoldFile_settings_info* (page 54) for descriptions.

write_fh(**args: Any*[446], ***kwargs: Any*[447]) → None[448]

Write directly to the underlying file handle.

2.1.6 HybFoldIter Class

class hybkit.HybFoldIter(*hybfile_handle: HybFile (page 21), foldfile_handle: FoldFile, combine: bool*[449] *= False, iter_error_mode: Optional*[450]*[Literal*[451]*['raise', 'warn_return', 'warn_skip', 'skip', 'return']] = None*)

Iterator for simultaneous iteration over a `HybFile` (page 21) and `FoldFile` object.

This class provides an iterator to iterate through a `HybFile` (page 21) and one of a `ViennaFile` (page 29), or `CtFile` (page 31) simultaneously to return a `HybRecord` (page 9) and `FoldRecord` (page 23).

Basic error checking / catching is performed based on the value of the `~settings['error_mode']` (page 34) setting.

Parameters

- **hybfile_handle** (HybFile (page 21)) -- HybFile object for iteration

[416] https://docs.python.org/3/library/typing.html#typing.Any
[417] https://docs.python.org/3/library/typing.html#typing.Optional
[418] https://docs.python.org/3/library/typing.html#typing.Literal
[419] https://docs.python.org/3/library/typing.html#typing.Optional
[420] https://docs.python.org/3/library/typing.html#typing.Literal
[421] https://docs.python.org/3/library/functions.html#bool
[422] https://docs.python.org/3/library/typing.html#typing.Any
[423] https://docs.python.org/3/library/stdtypes.html#str
[424] https://docs.python.org/3/library/stdtypes.html#str
[425] https://docs.python.org/3/library/functions.html#bool
[426] https://docs.python.org/3/library/stdtypes.html#str
[427] https://docs.python.org/3/library/stdtypes.html#str
[428] https://docs.python.org/3/library/typing.html#typing.Union
[429] https://docs.python.org/3/library/typing.html#typing.Tuple
[430] https://docs.python.org/3/library/constants.html#None
[431] https://docs.python.org/3/library/stdtypes.html#str
[432] https://docs.python.org/3/library/typing.html#typing.Tuple
[433] https://docs.python.org/3/library/typing.html#typing.Literal
[434] https://docs.python.org/3/library/stdtypes.html#str
[435] https://docs.python.org/3/library/typing.html#typing.Tuple
[436] https://docs.python.org/3/library/typing.html#typing.Literal
[437] https://docs.python.org/3/library/stdtypes.html#str
[438] https://docs.python.org/3/library/constants.html#None
[439] https://docs.python.org/3/library/stdtypes.html#str
[440] https://docs.python.org/3/library/typing.html#typing.Any
[441] https://docs.python.org/3/library/typing.html#typing.Any
[442] https://docs.python.org/3/library/stdtypes.html#str
[443] https://docs.python.org/3/library/stdtypes.html#str
[444] https://docs.python.org/3/library/stdtypes.html#str
[445] https://docs.python.org/3/library/typing.html#typing.List
[446] https://docs.python.org/3/library/typing.html#typing.Any
[447] https://docs.python.org/3/library/typing.html#typing.Any
[448] https://docs.python.org/3/library/constants.html#None

- **foldfile_handle** (*ViennaFile* (page 29) or *CtFile* (page 31)) -- *ViennaFile* (page 29) or *CtFile* (page 31) object for iteration
- **combine** (bool[452], optional) -- Use HybRecord.set_fold_record(FoldRecord) and return only the HybRecord.
- **iter_error_mode** (*str*[453], *optional*) -- Error mode to use for reading *FoldRecord* (page 23) objects. If not set, defaults to the value in *settings['iter_error_mode']* (page 34).

Returns
(*HybRecord* (page 9), *FoldRecord* (page 23))

settings = {'error_checks': ['hybrecord_indel', 'foldrecord_nofold', 'max_mismatch', 'energy_mismatch'], 'iter_error_mode': 'warn_skip', 'max_sequential_skips': 100}

Class-level settings. See *settings.HybFoldIter_settings_info* (page 54) for descriptions.

report() → List[454][str[455]]

Return a report of information from iteration.

print_report() → None[456]

Print a report of information from iteration.

2.2 hybkit.type_finder

hybkit TypeFinder Class.

This module contains the TypeFinder class to work with HybRecord to parse sequence identifiers to identify sequence type.

class hybkit.type_finder.TypeFinder

Class for parsing identifiers to identify sequence 'type'.

Designed to be used by the *hybkit.HybRecord* (page 9)

Variables
params (*dict*[457]) -- Stored parameters for string parsing, where applicable.

find_with_params = None

Placeholder for storing active method, set with *set_method()* (page 35) (see *set_method()* (page 35) for details).

params = None

Placeholder for parameters for active method, set with *set_method()* (page 35) (see *set_method()* (page 35) for details).

---
449 https://docs.python.org/3/library/functions.html#bool
450 https://docs.python.org/3/library/typing.html#typing.Optional
451 https://docs.python.org/3/library/typing.html#typing.Literal
452 https://docs.python.org/3/library/functions.html#bool
453 https://docs.python.org/3/library/stdtypes.html#str
454 https://docs.python.org/3/library/typing.html#typing.List
455 https://docs.python.org/3/library/stdtypes.html#str
456 https://docs.python.org/3/library/constants.html#None

default_method = 'hybformat'

Default method assigned using *check_set_method()* (page 35)

methods = {'hybformat': 'method_hybformat', 'id_map': 'method_id_map', 'string_match': 'method_string_match'}

Dict of provided methods available to assign segment types

'hybformat'	*method_hybformat()* (page 36)
'string_match'	*method_string_match()* (page 37)
'id_map'	*method_id_map()* (page 38)

param_methods = {'hybformat': None, 'id_map': 'make_id_map_params', 'string_match': 'make_string_match_params'}

Dict of param generation methods for type finding methods

'hybformat'	'N/A'
'string_match'	*make_string_match_params()* (page 37)
'id_map'	*make_id_map_params()* (page 38)

param_methods_needs_file = {'hybformat': False, 'id_map': True, 'string_match': True}

Dict of whether parameter generation methods need an input file

'hybformat'	False[458]
'string_match'	True[459]
'id_map'	True[460]

classmethod set_method(*method: str*[461], *params: Optional*[462]*[Dict*[463]*[str*[464], *Any*[465]*]] = None*) → None[466]

Select method to use when finding types.

Available methods are listed in *methods* (page 35).

Parameters

- **method** (*str*[467]) -- Method option from *methods* (page 35) to set for use as *find()* (page 35).
- **params** (*dict*[468], *optional*) -- Dict object of parameters to use by set method.

classmethod method_is_set() → bool[469]

Return whether a TypeFinder method has been set.

Methods should be set with *set_method()* (page 35).

Returns

True if a method has been set, False otherwise.

Return type

bool[470]

classmethod check_set_method() → None[471]

If no TypeFinder method set, set as *default_method* (page 34).

classmethod find(*seg_props: Dict[472][str[473], Union[474][float[475], int[476], str[477]]]*) → Optional[478][str[479]]

Find type of segment using `TypeFinder.find_custom_method()`.

If a TypeFinder method has been set with *set_method()* (page 35). use the current parameters set in *params* (page 34) to find the type of the provided segment by calling:

```
seg_type = :meth:`TypeFinder.find_custom_method`(seg_props, :attr`TypeFinder.
↪params`)
```

Parameters
seg_props (*dict*[480]) -- seg_props from *hybkit.HybRecord* (page 9)

Returns
Type of the provided segment, or None if a type cannot be identified.

Return type
str[481]

classmethod set_custom_method(*method: Callable[482], params: Optional[483][dict[484]] = None*) → None[485]

Set the method for use to find seg types.

This method is for providing a custom function. To use the included functions, use *set_method()* (page 35). Custom functions provided must have the signature:

```
seg_type = custom_method(self, seg_props, params)
```

This function should return the string of the assigned segment type if found, or a None object if the type cannot be found. It can also take a dictionary in the "params" argument that specifies additional or dynamic search properties, as desired.

Parameters

- **method** (*method*) -- Method to set for use.
- **params** (*dict*[486]*, optional*) -- dict of custom parameters to set for use.

static method_hybformat(*seg_props: Dict[487][str[488], Union[489][float[490], int[491], str[492]]], params: Optional[493][dict[494]] = None*) → Optional[495][str[496]]

Return the type of the provided segment, or None if segment cannot be identified.

This method works with sequence / alignment mapping identifiers in the format of the reference database provided by the Hyb Software Package, specifically identifiers of the format:

```
<gene_id>_<transcript_id>_<gene_name>_<seg_type>
```

This method returns the last component of the identifier, split by "_", as the identified sequence type. (returns None[497] if the segment identifier does not contain "_").

Example

```
"MIMAT0000076_MirBase_miR-21_microRNA"  --->  "microRNA".
```

Parameters

- **seg_props** (*dict*[498]) -- seg_props from *hybkit.HybRecord* (page 9)
- **params** (*dict*[499], *optional*) -- Unused in this method.

static method_string_match(*seg_props: Dict*[500]*[str*[501]*, Union*[502]*[float*[503]*, int*[504]*, str*[505]*]], params: Optional*[506]*[dict*[507]*] = None*) → Optional[508][str[509]]

Return the type of the provided segment, or None if unidentified.

This method attempts to find a string matching a specific pattern within the identifier of the aligned segment. Search options include "startswith", "contains", "endswith", and "matches", and returns the first type matching the criteria. The required params dict should contain a key for each desired search type, with a list of 2-tuples for each search-string with assigned-type.

Example

```
params = {'endswith': [('_miR', 'microRNA'),
                       ('_trans', 'mRNA')   ]}
```

This dict can be generated with the associated *make_string_match_params()* (page 37) method and an associated csv legend file with format:

```
#comment line
#search_type,search_string,seg_type
endswith,_miR,microRNA
endswith,_trans,mRNA
```

Parameters

- **seg_props** (*dict*[510]) -- *HybRecord* (page 9) segment properties dict to evaluate.
- **params** (*dict*[511], *optional*) -- Dict with search parameters as described above.

static make_string_match_params(*legend_file: str*[512]) → dict[513]

Read csv and return a dict of search parameters for *method_string_match()* (page 37).

The my_legend.csv file should have the format:

```
#comment line
#search_type,search_string,seg_type
endswith,_miR,microRNA
endswith,_trans,mRNA
```

Search_type options include "startswith", "contains", "endswith", and "matches" The produced dict object contains a key for each search type, with a list of 2-tuples for each search-string and associated segment-type.

For example:

```
{'endswith': [('_miR', 'microRNA'),
              ('_trans', 'mRNA')   ]}
```

static method_id_map(*seg_props: Dict[514][str[515], Union[516][float[517], int[518], str[519]]], params: Optional[520][dict[521]] = None*) → Optional[522][str[523]]

Return the type of the provided segment or None if it cannot be identified.

This method checks to see if the identifier of the segment is present in the params dict. params should be formatted as a dict with keys as sequence identifier names, and the corresponding type as the respective values.

Example

```
params = {'MIMAT0000076_MirBase_miR-21_microRNA': 'microRNA',
          'ENSG00000XXXXXX_NR003287-2_RN28S1_rRNA': 'rRNA'}
```

This dict can be generated with the associated *make_id_map_params()* (page 38) method.

Parameters

- **seg_props** (*dict*[524]) -- *HybRecord* (page 9) segment properties dict to evaluate.
- **params** (*dict*[525]) -- Dict of mapping of sequence identifiers to sequence types.

Returns
Identified sequence type, or None if it cannot be found.

Return type
str[526]

static make_id_map_params(*mapped_id_files: List[527][str[528]]*) → dict[529]

Read file(s) into a mapping of sequence identifiers.

This method reads one or more files into a dict for use with the *method_id_map()* (page 38) method. The method requires passing a file path (or list/tuple of file paths) of mapped_id_files. Files listed in the mapped_id_files argument should have the format:

```
#comment line
#seg_id,seg_type
segA_unique_id,segA_type
segB_unique_id,segB_type
```

Parameters
mapped_id_files (str[530], list[531], or tuple[532]) -- Iterable object containing strings of paths to files containing id/type mapping information.

[457] https://docs.python.org/3/library/stdtypes.html#dict
[458] https://docs.python.org/3/library/constants.html#False
[459] https://docs.python.org/3/library/constants.html#True
[460] https://docs.python.org/3/library/constants.html#True
[461] https://docs.python.org/3/library/stdtypes.html#str
[462] https://docs.python.org/3/library/typing.html#typing.Optional
[463] https://docs.python.org/3/library/typing.html#typing.Dict
[464] https://docs.python.org/3/library/stdtypes.html#str
[465] https://docs.python.org/3/library/typing.html#typing.Any
[466] https://docs.python.org/3/library/constants.html#None
[467] https://docs.python.org/3/library/stdtypes.html#str
[468] https://docs.python.org/3/library/stdtypes.html#dict
[469] https://docs.python.org/3/library/functions.html#bool
[470] https://docs.python.org/3/library/functions.html#bool
[471] https://docs.python.org/3/library/constants.html#None
[472] https://docs.python.org/3/library/typing.html#typing.Dict
[473] https://docs.python.org/3/library/stdtypes.html#str
[474] https://docs.python.org/3/library/typing.html#typing.Union
[475] https://docs.python.org/3/library/functions.html#float
[476] https://docs.python.org/3/library/functions.html#int
[477] https://docs.python.org/3/library/stdtypes.html#str
[478] https://docs.python.org/3/library/typing.html#typing.Optional
[479] https://docs.python.org/3/library/stdtypes.html#str
[480] https://docs.python.org/3/library/stdtypes.html#dict
[481] https://docs.python.org/3/library/stdtypes.html#str
[482] https://docs.python.org/3/library/typing.html#typing.Callable
[483] https://docs.python.org/3/library/typing.html#typing.Optional
[484] https://docs.python.org/3/library/stdtypes.html#dict
[485] https://docs.python.org/3/library/constants.html#None
[486] https://docs.python.org/3/library/stdtypes.html#dict
[487] https://docs.python.org/3/library/typing.html#typing.Dict
[488] https://docs.python.org/3/library/stdtypes.html#str
[489] https://docs.python.org/3/library/typing.html#typing.Union
[490] https://docs.python.org/3/library/functions.html#float
[491] https://docs.python.org/3/library/functions.html#int
[492] https://docs.python.org/3/library/stdtypes.html#str
[493] https://docs.python.org/3/library/typing.html#typing.Optional
[494] https://docs.python.org/3/library/stdtypes.html#dict
[495] https://docs.python.org/3/library/typing.html#typing.Optional
[496] https://docs.python.org/3/library/stdtypes.html#str
[497] https://docs.python.org/3/library/constants.html#None
[498] https://docs.python.org/3/library/stdtypes.html#dict
[499] https://docs.python.org/3/library/stdtypes.html#dict
[500] https://docs.python.org/3/library/typing.html#typing.Dict
[501] https://docs.python.org/3/library/stdtypes.html#str
[502] https://docs.python.org/3/library/typing.html#typing.Union
[503] https://docs.python.org/3/library/functions.html#float
[504] https://docs.python.org/3/library/functions.html#int
[505] https://docs.python.org/3/library/stdtypes.html#str
[506] https://docs.python.org/3/library/typing.html#typing.Optional
[507] https://docs.python.org/3/library/stdtypes.html#dict
[508] https://docs.python.org/3/library/typing.html#typing.Optional
[509] https://docs.python.org/3/library/stdtypes.html#str
[510] https://docs.python.org/3/library/stdtypes.html#dict
[511] https://docs.python.org/3/library/stdtypes.html#dict
[512] https://docs.python.org/3/library/stdtypes.html#str
[513] https://docs.python.org/3/library/stdtypes.html#dict
[514] https://docs.python.org/3/library/typing.html#typing.Dict
[515] https://docs.python.org/3/library/stdtypes.html#str
[516] https://docs.python.org/3/library/typing.html#typing.Union
[517] https://docs.python.org/3/library/functions.html#float
[518] https://docs.python.org/3/library/functions.html#int
[519] https://docs.python.org/3/library/stdtypes.html#str
[520] https://docs.python.org/3/library/typing.html#typing.Optional
[521] https://docs.python.org/3/library/stdtypes.html#dict
[522] https://docs.python.org/3/library/typing.html#typing.Optional
[523] https://docs.python.org/3/library/stdtypes.html#str
[524] https://docs.python.org/3/library/stdtypes.html#dict
[525] https://docs.python.org/3/library/stdtypes.html#dict
[526] https://docs.python.org/3/library/stdtypes.html#str
[527] https://docs.python.org/3/library/typing.html#typing.List
[528] https://docs.python.org/3/library/stdtypes.html#str
[529] https://docs.python.org/3/library/stdtypes.html#dict
[530] https://docs.python.org/3/library/stdtypes.html#str

2.3 hybkit.analysis

Functions for analysis of HybRecord and FoldRecord objects.

2.3.1 Analysis

class hybkit.analysis.**Analysis**(*analysis_types: Union[533][Literal[534]['energy', 'type', 'mirna', 'target'], List[535][Literal[536]['energy', 'type', 'mirna', 'target']]], name: Optional[537][str[538]] = None, quant_mode: Optional[539][Literal[540]['single', 'reads', 'records']] = None*)

Class for analysis of hybkit HybRecord and FoldRecord objects.

This class contains multiple conceptual analyses for HybRecord/FoldRecord Data:

Energy (page 40): Analysis of values of predicted intra-hybrid folding energy

Type (page 40): Analysis of segment types

miRNA (page 41): Analysis of miRNA segments distributions

Target (page 41): Analysis of mirna target segment names and types

Fold (page 42): Analysis of folding data included in the analyzed hyb_records.

This class used by selecting the desired analysis types on object initialization. Analyses are performed either by using either the `add_record()` or the `add_all_records()` methods. The results of the analysis are then available through the `get_all_results()` (page 43), `get_analysis_results()` (page 43), `get_specific_result()` (page 43), and `plot_analysis_results()` (page 44) methods, which can return (or plot) the results of all analyses or of a specific subset of analyses.

Details for each respective analysis are provided here:

Energy Analysis:

This analysis evaluates the energy of each `HybRecord` (page 9) object and provides a binned-histogram of all energy values represented.

Output Results:

`energy_analysis_count` (`int`[541]): Count of energy values evaluated

`has_energy_val` (`int`[542]): Count of hyb_records with an energy value

`no_energy_val` (`int`[543]): Count of hyb_records without an energy value

`energy_min` (`float`[544]): Minimum energy value

`energy_max` (`float`[545]): Maximum energy value

`energy_mean` (`float`[546]): Mean energy value

`energy_std` (`float`[547]): Standard deviation of energy values

`binned_energy_vals` (`Counter`[548]): Counter with integer keys of energy values from `energy_min` to `energy_max` storing the count of any hyb_records with energy values that fall within that range (rounded to the next highest integer (e.g. -12.5 -> -12).

Type Analysis:

This analysis evaluates the counts of each type of segment included in the `HybRecord` (page 9) objects. The types of segments are determined by the *seg1_type* (page 6) and *seg2_type* (page 6) flags, which are set by the `hybkit.HybRecord.eval_types()` (page 14) method.

Requirements:

seg1_type (page 6) and *seg2_type* (page 6) flags must be set for each HybRecord, (can be done by `hybkit.HybRecord.eval_types()` (page 14)).

Output Results:

`types_analysis_count` (`int`[549]): Count of hybrid types analyzed

`hybrid_types` (`Counter`[550]): Counter containing annotated types of seg1 and seg (in original 5P (5-Prime) / 3P (3-Prime) order)

`reordered_hybrid_types` (`Counter`[551]): Counter containing annotated types of seg1 and seg2. This is provided in "sorted" order, where types are sorted alphabetically (independent of 5P / 3P position).

`mirna_hybrid_types` (`Counter`[552]): Counter containing annotated types of seg1 and seg2. This is provided in "miRNA-prime" order, where a miRNA type is always listed before other types, and then remaining types are sorted alphabetically (independent of 5P / 3P position).

`seg1_types` (`Counter`[553]): Counter containing annotated type of segment in position seg1

`seg2_types` (`Counter`[554]): Counter containing annotated type of segment in position seg2

`all_seg_types` (`Counter`[555]): Counter containing position-independent annotated types

miRNA Analysis:

Analysis of miRNA segments in hybrids.

The mirna_analysis provides an analysis of what miRNA types are present in the hyb records. If a miRNA dimer is present in a hybrid, this is counted in `mirna_dimers`. If a single miRNA is present in a hybrid, this is counted in `mirnas_5p` or `mirnas_3p` depending on the miRNA location.

Requirements:

mirna_seg (page 6) flag must be set for each HybRecord (can be done by `hybkit.HybRecord.eval_mirna()` (page 15)).

Output Results:

`mirna_analysis_count` (`int`[556]): Count of miRNA hybrids analyzed

`mirnas_5p` (`int`[557]): Count of 5P miRNAs detected

`mirnas_3p` (`int`[558]): Count of 3P miRNAs detected

`mirna_dimers` (`int`[559]): Count of miRNA dimers (5P + 3P) detected

`non_mirna` (`int`[560]): Count of non-miRNA hybrids detected

`has_mirna` (`int`[561]): Hybrids with 5P, 3P, or both as miRNA

Target Analysis:

Analysis of targets in miRNA-containing hybrids.

The target analysis provides an analysis of what annotated sequences and sequence types are targeted by any miRNA within the hyb records. If a miRNA is not present in a hybrid, the hybrid is not included in the analysis. If a miRNA dimer is present in a hybrid, the 5P miRNA is used for the analysis, and the 3P miRNA is considered the "target."

Requirements:

mirna_seg (page 6) flag must be set for each HybRecord (can be done by `hybkit.HybRecord.eval_mirna()` (page 15)).

Output Results:

`target_analysis_count` (`int`[562]): Count of hybrids analyzed
`target_evals` (`int`[563]): Count of target evaluations performed
`target_names` (`Counter`[564]): Counter containing names of miRNA targets detected.
`target_types` (`Counter`[565]): Counter containing types of miRNA targets detected.

Fold Analysis:

This analysis evaluates the predicted binding of miRNA within hyb records that contain a miRNA and have an associated *`FoldRecord`* (page 23) object as the attribute `fold_record`. This includes an analysis and plotting of the predicted binding by position among the provided miRNA.

Requirements:

The *mirna_seg* (page 6) flag must be set for each HybRecord (can be done by *`hybkit.HybRecord.eval_mirna()`* (page 15)).
The *fold_record* (page 10) attribute must be set for each HybRecord with a corresponding *`FoldRecord`* (page 23) object. This can be done using the *`hybkit.HybRecord.set_fold_record()`* (page 14) method.

Output Results:

`fold_analysis_count` (`int`[566]): Count of miRNA fold predictions analyzed
`folds_recorded` (`int`[567]): Count of fold predictions with a mirna fold
`mirna_nt_fold_counts` (`Counter`[568]) : Counter with keys of miRNA position index and values of number of miRNAs with a predicted bound state at that index.
`mirna_nt_fold_props` (`Counter`[569]) : Counter with keys of miRNA position index and values of proportion (0.0 - 1.0) of miRNAs with a predicted bound state at that index.
`fold_match_counts` (`Counter`[570]) : Counter with keys of count of predicted matches between miRNA and target with values of count of miRNAs with that number of predicted matches.

Parameters

- **analysis_types** (`str`[571] or `list`[572] of `str`[573]) -- Analysis types to perform
- **name** (`str`[574], optional) -- Name of the analysis
- **quant_mode** (`str`[575], optional) -- Mode to use for record quantification. Options are "single": One count per record; "reads": If "read_count" flag is set, count all reads in record (else count 1); "records": if the "record_count" flag is set, count all individual records within combined record (else count 1). If not provided, defaults to the value in `Analysis.settings['quant_mode']`.

Variables

- **name** (`str`[576]) -- Name of the analysis
- **analysis_types** (`list`[577] of `str`[578]) -- List of analysis types to perform
- **quant_mode** (`str`[579]) -- Mode to use for record quantification.

settings = {'out_delim': ',', 'quant_mode': 'single'}

Class-level settings. See *`hybkit.settings.Analysis_settings`* (page 56) for descriptions.

analysis_options = ['energy', 'type', 'mirna', 'target', 'fold']

add_hyb_record(*hyb_record:* HybRecord *(page 9)*) → None[580]

Add a HybRecord object to the analysis.

Parameters

hyb_record (*HybRecord* (page 9)) -- HybRecord object to be added to the analysis.

add_hyb_records(*hyb_records: List*[581]*[*HybRecord *(page 9)], eval_types: bool*[582] *= False, eval_mirna: bool*[583] *= False*) → None[584]

Add a list of HybRecord objects to the analysis.

Parameters

- **hyb_records** (*HybFile* (page 21) or list[585] of *HybRecord* (page 9)) -- HybFile to iterate over, or iterable of HybRecord objects to be added to the analysis.
- **eval_types** (*bool*[586]) -- If `True`, evaluate the hybrid type of the HybRecord before adding it to the analysis using *hybkit.HybRecord.eval_types()* (page 14).
- **eval_mirna** (*bool*[587]) -- If `True`, evaluate the miRNA segment of the HybRecord before adding it to the analysis using *hybkit.HybRecord.eval_mirna()* (page 15).

get_all_results() → dict[588]

Return a dictionary with all results for all active analyses.

See *Analyses* (page 40) for details on the results for each analysis type.

Returns

Dictionary with keys of analysis type and values of
dictionaries with results for that analysis type.

Return type

dict[589]

get_analysis_results(*analysis: Literal*[590]*['energy', 'type', 'mirna', 'target']*) → Dict[591]

Return a dictionary with all results for a specific analysis.

See *Analyses* (page 40) for details on the results for each analysis type.

Parameters

analysis (*str*[592]) -- Analysis type to return results for.

Returns

Dictionary with results for the specified analysis type.
see :ref:Analyses for details.

Return type

dict[593]

get_specific_result(*result_key: str*[594]) → Any[595]

Get a specific result from the analysis.

See *Analyses* (page 40) for details on the results for each analysis type.

Parameters

result_key (*str*[596]) -- Result key to return from one of the enabled analyses.

Returns

Result value for the specified result key.

get_analysis_delim_str(*analysis: Optional[597][Literal[598]['energy', 'type', 'mirna', 'target']] = None, out_delim: Optional[599][str[600]] = None*) → str[601]

Return a delimited string containing the results of the analysis.

See *Analyses* (page 40) for details on the results for each analysis type.

Parameters

- **analysis** (`str`[602] or `list`[603] of `str`[604]) -- Analysis type for return results. If not provided, return the results for all active analyses.
- **out_delim** (*str*[605]) -- Delimiter to use for output. If not provided, defaults to the value in `settings['out_delim']` (page 42).

write_analysis_delim_str(*out_file_name: Optional[606][str[607]] = None, analysis: Optional[608][Union[609][Literal[610]['energy', 'type', 'mirna', 'target'], List[611][Literal[612]['energy', 'type', 'mirna', 'target']]]] = None, out_delim: Optional[613][str[614]] = None*) → None[615]

Write the results of the analysis to a delimited text file.

See *Analyses* (page 40) for details on the results for each analysis type.

Parameters

- **out_file_name** (*str*[616]) -- Path to output file. If not provided, defaults to: ./<analysis_name>_<analysis>.csv if analysis/analyses provided, or ./<analysis_name>_multi_analysis.csv if no analysis/analyses provided.
- **analysis** (`str`[617] or `list`[618] of `str`[619]) -- Analysis type for return results. If not provided, return the results for all active analyses.
- **out_delim** (*str*[620]) -- Delimiter to use for output. If not provided, defaults to the value in `settings['out_delim']` (page 42).

write_analysis_results_special(*out_basename: Optional[621][str[622]] = None, analysis: Optional[623][Union[624][Literal[625]['energy', 'type', 'mirna', 'target'], List[626][Literal[627]['energy', 'type', 'mirna', 'target']]]] = None, out_delim: Optional[628][str[629]] = None*) → List[630][str[631]]

Write the results of the analyses to specialized text files.

See *Analyses* (page 40) for details on the results for each analysis type.

Parameters

- **out_basename** (*str*[632]) -- Path for basename of output file. Files will be renamed using the provided path as the base name. If not provided, defaults to: ./<analysis_name>_<analysis> if `name` is set, or ./Analysis_multi_<analysis> if name not set.
- **analysis** (`str`[633] or `list`[634] of `str`[635]) -- Analysis type to write results files for. If not provided, write results files for all active analyses.
- **out_delim** (*str*[636]) -- Delimiter to use for output where applicable. If not provided, defaults to the value in `settings['out_delim']` (page 42).

plot_analysis_results(*out_basename: Optional[637][str[638]] = None, analysis: Optional[639][Union[640][Literal[641]['energy', 'type', 'mirna', 'target'], List[642][Literal[643]['energy', 'type', 'mirna', 'target']]]] = None*) → List[644][str[645]]

Plot the results of the analyses.

See *Analyses* (page 40) for details on the results for each analysis type.

Parameters

- **analysis** (str[646] or list[647] of str[648]) -- Analysis type to plot results for. If not provided, plot results for all active analyses.
- **out_basename** (*str*[649]) -- Path to output file. If not provided, defaults to: ./<analysis_name> if `name` provided or ./analysis if no name provided.

`key = 'fold'`

[533] https://docs.python.org/3/library/typing.html#typing.Union
[534] https://docs.python.org/3/library/typing.html#typing.Literal
[535] https://docs.python.org/3/library/typing.html#typing.List
[536] https://docs.python.org/3/library/typing.html#typing.Literal
[537] https://docs.python.org/3/library/typing.html#typing.Optional
[538] https://docs.python.org/3/library/stdtypes.html#str
[539] https://docs.python.org/3/library/typing.html#typing.Optional
[540] https://docs.python.org/3/library/typing.html#typing.Literal
[541] https://docs.python.org/3/library/functions.html#int
[542] https://docs.python.org/3/library/functions.html#int
[543] https://docs.python.org/3/library/functions.html#int
[544] https://docs.python.org/3/library/functions.html#float
[545] https://docs.python.org/3/library/functions.html#float
[546] https://docs.python.org/3/library/functions.html#float
[547] https://docs.python.org/3/library/functions.html#float
[548] https://docs.python.org/3/library/collections.html#collections.Counter
[549] https://docs.python.org/3/library/functions.html#int
[550] https://docs.python.org/3/library/collections.html#collections.Counter
[551] https://docs.python.org/3/library/collections.html#collections.Counter
[552] https://docs.python.org/3/library/collections.html#collections.Counter
[553] https://docs.python.org/3/library/collections.html#collections.Counter
[554] https://docs.python.org/3/library/collections.html#collections.Counter
[555] https://docs.python.org/3/library/collections.html#collections.Counter
[556] https://docs.python.org/3/library/functions.html#int
[557] https://docs.python.org/3/library/functions.html#int
[558] https://docs.python.org/3/library/functions.html#int
[559] https://docs.python.org/3/library/functions.html#int
[560] https://docs.python.org/3/library/functions.html#int
[561] https://docs.python.org/3/library/functions.html#int
[562] https://docs.python.org/3/library/functions.html#int
[563] https://docs.python.org/3/library/functions.html#int
[564] https://docs.python.org/3/library/collections.html#collections.Counter
[565] https://docs.python.org/3/library/collections.html#collections.Counter
[566] https://docs.python.org/3/library/functions.html#int
[567] https://docs.python.org/3/library/functions.html#int
[568] https://docs.python.org/3/library/collections.html#collections.Counter
[569] https://docs.python.org/3/library/collections.html#collections.Counter
[570] https://docs.python.org/3/library/collections.html#collections.Counter
[571] https://docs.python.org/3/library/stdtypes.html#str
[572] https://docs.python.org/3/library/stdtypes.html#list
[573] https://docs.python.org/3/library/stdtypes.html#str
[574] https://docs.python.org/3/library/stdtypes.html#str
[575] https://docs.python.org/3/library/stdtypes.html#str
[576] https://docs.python.org/3/library/stdtypes.html#str
[577] https://docs.python.org/3/library/stdtypes.html#list
[578] https://docs.python.org/3/library/stdtypes.html#str
[579] https://docs.python.org/3/library/stdtypes.html#str
[580] https://docs.python.org/3/library/constants.html#None
[581] https://docs.python.org/3/library/typing.html#typing.List
[582] https://docs.python.org/3/library/functions.html#bool
[583] https://docs.python.org/3/library/functions.html#bool
[584] https://docs.python.org/3/library/constants.html#None
[585] https://docs.python.org/3/library/stdtypes.html#list
[586] https://docs.python.org/3/library/functions.html#bool
[587] https://docs.python.org/3/library/functions.html#bool
[588] https://docs.python.org/3/library/stdtypes.html#dict
[589] https://docs.python.org/3/library/stdtypes.html#dict
[590] https://docs.python.org/3/library/typing.html#typing.Literal
[591] https://docs.python.org/3/library/typing.html#typing.Dict
[592] https://docs.python.org/3/library/stdtypes.html#str
[593] https://docs.python.org/3/library/stdtypes.html#dict
[594] https://docs.python.org/3/library/stdtypes.html#str
[595] https://docs.python.org/3/library/typing.html#typing.Any
[596] https://docs.python.org/3/library/stdtypes.html#str
[597] https://docs.python.org/3/library/typing.html#typing.Optional
[598] https://docs.python.org/3/library/typing.html#typing.Literal
[599] https://docs.python.org/3/library/typing.html#typing.Optional
[600] https://docs.python.org/3/library/stdtypes.html#str
[601] https://docs.python.org/3/library/stdtypes.html#str
[602] https://docs.python.org/3/library/stdtypes.html#str
[603] https://docs.python.org/3/library/stdtypes.html#list
[604] https://docs.python.org/3/library/stdtypes.html#str
[605] https://docs.python.org/3/library/stdtypes.html#str
[606] https://docs.python.org/3/library/typing.html#typing.Optional

2.4 hybkit.plot

Methods for plotting analyses of HybRecord and FoldRecord objects.

hybkit.plot.**COLOR_DICT = {'Blue': '#0072B2', 'Bluish Green': '#009E73', 'Orange': '#E69F00', 'Reddish Purple': '#CC79A7', 'Sky Blue': '#56B4E9', 'Vermilion': '#D55E00', 'Yellow': '#F0E442'}**

Default Colors for colored plots: Colors selected based on "Points of view: Color blindness" by Bang Wong, Nature Methods, 2011. Colors in RGB nomenclature (1-255): Black (0,0,0), Orange (230,159,0), Sky Blue (86,180,233), Bluish Green (0,158,115), Yellow (240,228,66), Blue (0,114,178), Vermilion (213,94,0), Reddish Purple (204,121,167)

hybkit.plot.**COLOR_LIST = ['#0072B2', '#D55E00', '#009E73', '#CC79A7', '#E69F00', '#56B4E9', '#F0E442']**

List of default colors for colored plots.

hybkit.plot.**ENERGY_HIST_RC_PARAMS = {'axes.labelweight': 'bold', 'axes.titlepad': 15, 'axes.titlesize': 'large', 'axes.titleweight': 'bold', 'figure.dpi': 1200, 'figure.figsize': [6.4, 4.8]}**

Default mpl rcParams for energy analysis histograms.

hybkit.plot.**TYPE_PIE_SINGLE_RC_PARAMS = {'axes.labelweight': 'bold', 'axes.titlepad': 15, 'axes.titleweight': 'bold', 'figure.dpi': 1200, 'figure.figsize': [6.4, 4.8]}**

Default mpl rcParams for type analysis pie charts.

hybkit.plot.**TYPE_PIE_DUAL_RC_PARAMS = {'axes.labelweight': 'bold', 'axes.titlepad': 15, 'axes.titleweight': 'bold', 'figure.dpi': 1200, 'figure.figsize': (8, 4.8)}**

Default mpl rcParams for type analysis pie charts.

hybkit.plot.**TARGET_PIE_RC_PARAMS = {'axes.labelweight': 'bold', 'axes.titlepad': 15, 'axes.titleweight': 'bold', 'figure.dpi': 1200, 'figure.figsize': (9.6, 4.8)}**

Default mpl rcParams for target analysis pie charts.

hybkit.plot.**FOLD_MATCH_HIST_RC_PARAMS = {'axes.labelweight': 'bold', 'axes.titlepad': 15, 'axes.titlesize': 'large', 'axes.titleweight': 'bold', 'figure.dpi': 1200, 'figure.figsize': [6.4, 4.8]}**

Default mpl rcParams for fold match analysis histograms.

hybkit.plot.**FOLD_NT_COUNTS_HIST_RC_PARAMS = {'axes.labelweight': 'bold', 'axes.titlepad': 15, 'axes.titlesize': 'large', 'axes.titleweight': 'bold', 'figure.dpi': 1200, 'figure.figsize': [6.4, 4.8]}**

Default mpl rcParams for fold nt counts analysis histograms.

hybkit.plot.**PIE_DEFAULTS = {'COLORS': ['#0072B2', '#D55E00', '#009E73', '#CC79A7', '#E69F00', '#56B4E9', '#F0E442'], 'MIN_WEDGE_SIZE': 0.04, 'OTHER_THRESHOLD': 0.05, 'SETTINGS': {'autopct': '%1.1f%%', 'counterclock': False, 'shadow': False, 'startangle': 90}}**

Default Pie Chart Plot Settings.

hybkit.plot.**BAR_DEFAULTS = {'BAR_ALIGN': 'edge', 'BAR_EDGE_COLOR': None, 'BAR_WIDTH': 0.9}**

Default Bar Chart Plot Settings.

hybkit.plot.**BAR_INT_DEFAULTS = {'BAR_ALIGN': 'center', 'BAR_EDGE_COLOR': None, 'BAR_WIDTH': 0.9}**

Default Bar Chart of Integer Plot Settings.

`hybkit.plot.ENERGY_DEFAULTS = {'MIN_COUNT': 0, 'MIN_DENSITY': 0.0, 'XLABEL': 'Hybrid Gibbs Free Energy (kcal/mol)', 'YLABEL': 'Hybrid Count'}`

Default Bar Chart Plot Settings for Energy Histograms.

`hybkit.plot.energy_histogram`(*results: Dict[650][str[651], Any[652]], plot_file_name: str[653], title: str[654], name: Optional[655][str[656]] = None, rc_params: Dict[657][str[658], Any[659]] = {'axes.labelweight': 'bold', 'axes.titlepad': 15, 'axes.titlesize': 'large', 'axes.titleweight': 'bold', 'figure.dpi': 1200, 'figure.figsize': [6.4, 4.8]}, bar_params: Dict[660][str[661], Any[662]] = {'BAR_ALIGN': 'edge', 'BAR_EDGE_COLOR': None, 'BAR_WIDTH': 0.9}*) → None[663]

Plot histogram of hybrid energies from an `Analysis` (page 40) fold analysis.

Parameters

- **results** (*dict*[664]) -- Dictionary of energy counts from an `Analysis` (page 40) fold analysis (Key: `binned_energy_vals`).
- **plot_file_name** (*str*[665]) -- Name of output file.
- **title** (*str*[666]) -- Title of plot.
- **name** (str[667], optional) -- Name of analysis to be included in plot title.
- **rc_params** (dict[668], optional) -- Dictionary of mpl rcParams. Defaults to *ENERGY_HIST_RC_PARAMS* (page 47).
- **bar_params** (dict[669], optional) -- Dictionary of bar plot parameters. Defaults to *BAR_DEFAULTS* (page 47).

`hybkit.plot.type_count`(*results: Counter[670], plot_file_name: str[671], title: str[672], name: Optional[673][str[674]] = None, join_entries: bool[675] = False, rc_params: Dict[676][str[677], Any[678]] = {'axes.labelweight': 'bold', 'axes.titlepad': 15, 'axes.titleweight': 'bold', 'figure.dpi': 1200, 'figure.figsize': [6.4, 4.8]}*) → None[679]

Plot pie chart of hybrid type counts from an `Analysis` (page 40) type analysis.

Parameters

- **results** (*Counter*[680]) -- Counter Object of type counts from an `Analysis` (page 40) type analysis.
- **plot_file_name** (*str*[681]) -- Name of output file.
- **title** (*str*[682]) -- Title of plot.

[650] https://docs.python.org/3/library/typing.html#typing.Dict
[651] https://docs.python.org/3/library/stdtypes.html#str
[652] https://docs.python.org/3/library/typing.html#typing.Any
[653] https://docs.python.org/3/library/stdtypes.html#str
[654] https://docs.python.org/3/library/stdtypes.html#str
[655] https://docs.python.org/3/library/typing.html#typing.Optional
[656] https://docs.python.org/3/library/stdtypes.html#str
[657] https://docs.python.org/3/library/typing.html#typing.Dict
[658] https://docs.python.org/3/library/stdtypes.html#str
[659] https://docs.python.org/3/library/typing.html#typing.Any
[660] https://docs.python.org/3/library/typing.html#typing.Dict
[661] https://docs.python.org/3/library/stdtypes.html#str
[662] https://docs.python.org/3/library/typing.html#typing.Any
[663] https://docs.python.org/3/library/constants.html#None
[664] https://docs.python.org/3/library/stdtypes.html#dict
[665] https://docs.python.org/3/library/stdtypes.html#str
[666] https://docs.python.org/3/library/stdtypes.html#str
[667] https://docs.python.org/3/library/stdtypes.html#str
[668] https://docs.python.org/3/library/stdtypes.html#dict
[669] https://docs.python.org/3/library/stdtypes.html#dict

- **name** (str[683], optional) -- Name of analysis to be included in plot title.
- **join_entries** (bool[684], optional) -- If True, join two-tuple pairs into a single string for plot labels.
- **rc_params** (dict[685], optional) -- Dictionary of mpl rcParams. Defaults to TYPE_PIE_RC_PARAMS.

hybkit.plot.**type_count_dual**(*results: Counter[686], plot_file_name: str[687], title: str[688], name: Optional[689][str[690]] = None, join_entries: bool[691] = False, rc_params: Dict[692][str[693], Any[694]] = {'axes.labelweight': 'bold', 'axes.titlepad': 15, 'axes.titleweight': 'bold', 'figure.dpi': 1200, 'figure.figsize': (8, 4.8)}*) → None[695]

Plot pie chart of hybrid type counts from an *Analysis* (page 40) type analysis.

Parameters

- **results** (*Counter*[696]) -- Counter Object of type counts from an *Analysis* (page 40) type analysis.
- **plot_file_name** (*str*[697]) -- Name of output file.
- **title** (*str*[698]) -- Title of plot.
- **name** (str[699], optional) -- Name of analysis to be included in plot title.
- **join_entries** (bool[700], optional) -- If True, join two-tuple pairs into a single string for plot labels.
- **rc_params** (dict[701], optional) -- Dictionary of mpl rcParams. Defaults to TYPE_PIE_RC_PARAMS.

[670] https://docs.python.org/3/library/collections.html#collections.Counter
[671] https://docs.python.org/3/library/stdtypes.html#str
[672] https://docs.python.org/3/library/stdtypes.html#str
[673] https://docs.python.org/3/library/typing.html#typing.Optional
[674] https://docs.python.org/3/library/stdtypes.html#str
[675] https://docs.python.org/3/library/functions.html#bool
[676] https://docs.python.org/3/library/typing.html#typing.Dict
[677] https://docs.python.org/3/library/stdtypes.html#str
[678] https://docs.python.org/3/library/typing.html#typing.Any
[679] https://docs.python.org/3/library/constants.html#None
[680] https://docs.python.org/3/library/collections.html#collections.Counter
[681] https://docs.python.org/3/library/stdtypes.html#str
[682] https://docs.python.org/3/library/stdtypes.html#str
[683] https://docs.python.org/3/library/stdtypes.html#str
[684] https://docs.python.org/3/library/functions.html#bool
[685] https://docs.python.org/3/library/stdtypes.html#dict
[686] https://docs.python.org/3/library/collections.html#collections.Counter
[687] https://docs.python.org/3/library/stdtypes.html#str
[688] https://docs.python.org/3/library/stdtypes.html#str
[689] https://docs.python.org/3/library/typing.html#typing.Optional
[690] https://docs.python.org/3/library/stdtypes.html#str
[691] https://docs.python.org/3/library/functions.html#bool
[692] https://docs.python.org/3/library/typing.html#typing.Dict
[693] https://docs.python.org/3/library/stdtypes.html#str
[694] https://docs.python.org/3/library/typing.html#typing.Any
[695] https://docs.python.org/3/library/constants.html#None
[696] https://docs.python.org/3/library/collections.html#collections.Counter
[697] https://docs.python.org/3/library/stdtypes.html#str
[698] https://docs.python.org/3/library/stdtypes.html#str
[699] https://docs.python.org/3/library/stdtypes.html#str
[700] https://docs.python.org/3/library/functions.html#bool
[701] https://docs.python.org/3/library/stdtypes.html#dict

`hybkit.plot.target_count`(**args*, ***kwargs*) → None[702]

Plot pie chart of target counts from an `Analysis` (page 40) type analysis.

Parameters

- **results** (*Counter*[703]) -- Counter Object of type counts from an `Analysis` (page 40) type analysis.
- **plot_file_name** (*str*[704]) -- Name of output file.
- **title** (*str*[705]) -- Title of plot.
- **name** (str[706], optional) -- Name of analysis to be included in plot title.
- **join_entries** (bool[707], optional) -- If True, join two-tuple pairs into a single string for plot labels.
- **rc_params** (dict[708], optional) -- Dictionary of mpl rcParams. Defaults to *TARGET_PIE_RC_PARAMS* (page 47).

`hybkit.plot.fold_match_counts_histogram`(*results: Counter*[709], *plot_file_name: str*[710], *title: str*[711], *name: Optional*[712]*[str*[713]*] = None*, *is_prop: bool*[714] *= False*, *rc_params: Dict*[715]*[str*[716]*, Any*[717]*] = {'axes.labelweight': 'bold', 'axes.titlepad': 15, 'axes.titlesize': 'large', 'axes.titleweight': 'bold', 'figure.dpi': 1200, 'figure.figsize': [6.4, 4.8]}*, *bar_params: Dict*[718]*[str*[719]*, Any*[720]*] = {'BAR_ALIGN': 'center', 'BAR_EDGE_COLOR': None, 'BAR_WIDTH': 0.9}*) → None[721]

Plot histogram of predicted miRNA/target match count.

Parameters

- **results** (*Counter*[722]) -- Counter Object of match counts from an `Analysis` (page 40) type analysis.
- **plot_file_name** (*str*[723]) -- Name of output file.
- **title** (*str*[724]) -- Title of plot.
- **is_prop** (bool[725], optional) -- If True, y axis is proportion.
- **name** (str[726], optional) -- Name of analysis to be included in plot title.
- **rc_params** (dict[727], optional) -- Dictionary of mpl rcParams. Defaults to *FOLD_MATCH_HIST_RC_PARAMS* (page 47).
- **bar_params** (dict[728], optional) -- Dictionary of bar plot parameters. Defaults to *BAR_INT_DEFAULTS* (page 47).

[702] https://docs.python.org/3/library/constants.html#None
[703] https://docs.python.org/3/library/collections.html#collections.Counter
[704] https://docs.python.org/3/library/stdtypes.html#str
[705] https://docs.python.org/3/library/stdtypes.html#str
[706] https://docs.python.org/3/library/stdtypes.html#str
[707] https://docs.python.org/3/library/functions.html#bool
[708] https://docs.python.org/3/library/stdtypes.html#dict

hybkit.plot.**fold_mirna_nt_counts_histogram**(**args*, ***kwargs*) → None[729]

Plot histogram of predicted miRNA/target match count.

Parameters

- **results** (*Counter*[730]) -- Counter Object of match counts from an *Analysis* (page 40) type analysis.
- **plot_file_name** (*str*[731]) -- Name of output file.
- **title** (*str*[732]) -- Title of plot.
- **is_prop** (bool[733], optional) -- If True, y axis is proportion.
- **name** (str[734], optional) -- Name of analysis to be included in plot title.
- **rc_params** (dict[735], optional) -- Dictionary of mpl rcParams. Defaults to *FOLD_NT_COUNTS_HIST_RC_PARAMS* (page 47).
- **bar_params** (dict[736], optional) -- Dictionary of bar plot parameters. Defaults to *BAR_INT_DEFAULTS* (page 47).

2.5 hybkit.settings

This module contains settings information for hybkit classes and methods.

hybkit.settings.**HYB_SUFFIXES = ['.hyb', '.Hyb', '.HYB']**

Allowed suffixes for "Hyb" files.

hybkit.settings.**VIENNA_SUFFIXES = ['.vienna', '.Vienna', '.VIENNA']**

Allowed suffixes for "Vienna" files.

709 https://docs.python.org/3/library/collections.html#collections.Counter
710 https://docs.python.org/3/library/stdtypes.html#str
711 https://docs.python.org/3/library/stdtypes.html#str
712 https://docs.python.org/3/library/typing.html#typing.Optional
713 https://docs.python.org/3/library/stdtypes.html#str
714 https://docs.python.org/3/library/functions.html#bool
715 https://docs.python.org/3/library/typing.html#typing.Dict
716 https://docs.python.org/3/library/stdtypes.html#str
717 https://docs.python.org/3/library/typing.html#typing.Any
718 https://docs.python.org/3/library/typing.html#typing.Dict
719 https://docs.python.org/3/library/stdtypes.html#str
720 https://docs.python.org/3/library/typing.html#typing.Any
721 https://docs.python.org/3/library/constants.html#None
722 https://docs.python.org/3/library/collections.html#collections.Counter
723 https://docs.python.org/3/library/stdtypes.html#str
724 https://docs.python.org/3/library/stdtypes.html#str
725 https://docs.python.org/3/library/functions.html#bool
726 https://docs.python.org/3/library/stdtypes.html#str
727 https://docs.python.org/3/library/stdtypes.html#dict
728 https://docs.python.org/3/library/stdtypes.html#dict
729 https://docs.python.org/3/library/constants.html#None
730 https://docs.python.org/3/library/collections.html#collections.Counter
731 https://docs.python.org/3/library/stdtypes.html#str
732 https://docs.python.org/3/library/stdtypes.html#str
733 https://docs.python.org/3/library/functions.html#bool
734 https://docs.python.org/3/library/stdtypes.html#str
735 https://docs.python.org/3/library/stdtypes.html#dict
736 https://docs.python.org/3/library/stdtypes.html#dict

`hybkit.settings.CT_SUFFIXES = ['.ct', '.Ct', '.CT']`

Allowed suffixes for "Connection-Table" files.

`hybkit.settings.FOLD_SUFFIXES = ['.vienna', '.Vienna', '.VIENNA', '.ct', '.Ct', '.CT']`

Allowed suffixes for "Vienna" and "Connection-Table" files.

`hybkit.settings.MIRNA_TYPES = ['miRNA', 'microRNA']`

Default miRNA types for use in `mirna_analysis()`.

`hybkit.settings.HybRecord_settings_info`

Information for settings of *HybRecord* (page 9) class. Copied into *HybRecord_settings* (page 55) for use at runtime.

```
hybkit.settings.HybRecord_settings_info = {
'allow_undefined_flags': {'Argp-Flag': None,
                           'Argp-Opts': {'const': True, 'nargs': '?'},
                           'Argp-Type': 'custom_bool_from_str',
                           'Def-Val': False,
                           'Desc.': 'Allow use of flags not defined in the '
                                    'hybkit-specification order when reading and '
                                    'writing hyb records. As the preferred '
                                    'alternative to using this setting, the '
                                    '--custom_flags argument can be be used to '
                                    'supply custom allowed flags.'},
 'allow_unknown_seg_types': {'Argp-Flag': None,
                             'Argp-Opts': {'const': True, 'nargs': '?'},
                             'Argp-Type': 'custom_bool_from_str',
                             'Def-Val': False,
                             'Desc.': 'Allow unknown segment types when assigning '
                                      'segment types.'},
 'custom_flags': {'Argp-Flag': None,
                  'Argp-Opts': {'nargs': '+'},
                  'Argp-Type': 'str',
                  'Def-Val': [],
                  'Desc.': 'Custom flags to allow in addition to those specified in '
                           'the hybkit specification.'},
 'hyb_placeholder': {'Argp-Flag': None,
                     'Argp-Opts': {},
                     'Argp-Type': 'str',
                     'Def-Val': '.',
                     'Desc.': 'placeholder character/string for missing data in hyb '
                              'files.'},
 'mirna_types': {'Argp-Flag': None,
                 'Argp-Opts': {'nargs': '+'},
                 'Argp-Type': 'str',
                 'Def-Val': ['miRNA', 'microRNA'],
                 'Desc.': '"seg_type" fields identifying a miRNA'},
 'reorder_flags': {'Argp-Flag': None,
                   'Argp-Opts': {},
                   'Argp-Type': 'custom_bool_from_str',
                   'Def-Val': True,
                   'Desc.': 'Re-order flags to the hybkit-specification order when '
                            'writing hyb records.'}
}
```

hybkit.settings.**HybFile_settings_info**

Information for settings of *HybFile* (page 21) class. Copied into *HybFile_settings* (page 55) for use at runtime.

```
hybkit.settings.HybFile_settings_info = {
'hybformat_id': {'Argp-Flag': None,
                  'Argp-Opts': {'const': True, 'nargs': '?'},
                  'Argp-Type': 'custom_bool_from_str',
                  'Def-Val': False,
                  'Desc.': 'The Hyb Software Package places further information in '
                           'the "id" field of the hybrid record that can be used to '
                           'infer the number of contained read counts. When set to '
                           'True, the identifiers will be parsed as: '
                           '"<read_id>_<read_count>"'},
 'hybformat_ref': {'Argp-Flag': None,
                   'Argp-Opts': {'const': True, 'nargs': '?'},
                   'Argp-Type': 'custom_bool_from_str',
                   'Def-Val': False,
                   'Desc.': 'The Hyb Software Package uses a reference database '
                            'with identifiers that contain sequence type and other '
                            'sequence information. When set to True, all hyb file '
                            'identifiers will be parsed as: '
                            '"<gene_id>_<transcript_id>_<gene_name>_<seg_type>"'}
}
```

hybkit.settings.**FoldRecord_settings_info**

Information for settings of *FoldRecord* (page 23) class. Copied into *FoldRecord_settings* (page 55) for use at runtime.

```
hybkit.settings.FoldRecord_settings_info = {
'allowed_mismatches': {'Argp-Flag': None,
                        'Argp-Opts': {},
                        'Argp-Type': 'int',
                        'Def-Val': 0,
                        'Desc.': 'For DynamicFoldRecords, allowed number of '
                                 'mismatches with a HybRecord.'},
 'error_mode': {'Argp-Flag': None,
                'Argp-Opts': {'choices': ['raise', 'warn_return', 'return']},
                'Argp-Type': 'str',
                'Def-Val': 'raise',
                'Desc.': 'Mode for handling errors during reading of HybFiles '
                         "(overridden by HybFoldIter.settings['iter_error_mode'] "
                         'when using HybFoldIter). Options: "raise": Raise an error '
                         'when encountered and exit program ; "warn_return": Print '
                         'a warning and return the error_value ; "return": Return '
                         'the error value with no program output. record is '
                         'encountered.'},
 'fold_placeholder': {'Argp-Flag': None,
                      'Argp-Opts': {},
                      'Argp-Type': 'str',
                      'Def-Val': '.',
                      'Desc.': 'Placeholder character/string for missing data for '
                               'reading/writing fold records.'},
```

(continues on next page)

(continued from previous page)

```
 'seq_type': {'Argp-Flag': '-y',
              'Argp-Opts': {'choices': ['static', 'dynamic']},
              'Argp-Type': 'str',
              'Def-Val': 'static',
              'Desc.': 'Type of fold record object to use. Options: "static": '
                       'FoldRecord, requires an exact sequence match to be paired '
                       'with a HybRecord; "dynamic": DynamicFoldRecord, requires a '
                       'sequence match to the "dynamic" annotated regions of a '
                       'HybRecord, and may be shorter/longer than the original '
                       'sequence.'}
}
```

hybkit.settings.**FoldFile_settings_info**

Information for settings of `FoldFile` class. Copied into *FoldFile_settings* (page 55) for use at runtime.

```
hybkit.settings.FoldFile_settings_info = {

}
```

hybkit.settings.**HybFoldIter_settings_info**

Information for settings of *HybFoldIter* (page 33) class. Copied into *HybFoldIter_settings* (page 56) for use at runtime.

```
hybkit.settings.HybFoldIter_settings_info = {
'error_checks': {'Argp-Flag': None,
                  'Argp-Opts': {'choices': ['hybrecord_indel',
                                            'foldrecord_nofold',
                                            'max_mismatch',
                                            'energy_mismatch']},
                  'Argp-Type': 'str',
                  'Def-Val': ['hybrecord_indel',
                              'foldrecord_nofold',
                              'max_mismatch',
                              'energy_mismatch'],
                  'Desc.': 'Error checks for simultaneous HybFile and FoldFile '
                           'parsing. Options: "hybrecord_indel": Error for '
                           'HybRecord objects where one/both sequences have '
                           'insertions/deletions in alignment, which prevents '
                           'matching of sequences; "foldrecord_nofold": Error when '
                           'failure in reading a fold_record object; '
                           '"max_mismatch": Error when mismatch between hybrecord '
                           'and foldrecord sequences is greater than FoldRecord '
                           '"allowed_mismatches" setting; "energy_mismatch": Error '
                           'when a mismatch exists between HybRecord and FoldRecord '
                           'energy values.'},
 'iter_error_mode': {'Argp-Flag': None,
                     'Argp-Opts': {'choices': ['raise',
                                               'warn_return',
                                               'warn_skip',
                                               'skip',
                                               'return']},
                     'Argp-Type': 'str',
```

(continues on next page)

(continued from previous page)

```
                        'Def-Val': 'warn_skip',
                        'Desc.': 'Mode for handling errors found during error checks. '
                                 'Overrides HybRecord "error_mode" setting when using '
                                 'HybFoldIter. Options: "raise": Raise an error when '
                                 'encountered; "warn_return": Print a warning and '
                                 'return the value; "warn_skip": Print a warning and '
                                 'continue to the next iteration; "skip": Continue to '
                                 'the next iteration without any output; "return": '
                                 'return the value without any error output;'},
 'max_sequential_skips': {'Argp-Flag': None,
                          'Argp-Opts': {},
                          'Argp-Type': 'int',
                          'Def-Val': 100,
                          'Desc.': 'Maximum number of record(-pairs) to skip in a '
                                   'row. Limited as several sequential skips '
                                   'usually indicates an issue with record '
                                   'formatting or a desynchronization between '
                                   'files.'}
}
```

hybkit.settings.**Analysis_settings_info**

Information for settings of `Analysis` class. Copied into *Analysis_settings* (page 56) for use at runtime.

```
hybkit.settings.Analysis_settings_info = {
'out_delim': {'Argp-Flag': None,
                'Argp-Opts': {},
                'Argp-Type': 'str',
                'Def-Val': ',',
                'Desc.': 'Delimiter-string to place between fields in analysis '
                         'output.'},
 'quant_mode': {'Argp-Flag': None,
                'Argp-Opts': {'choices': ['single', 'reads', 'records']},
                'Argp-Type': 'str',
                'Def-Val': 'single',
                'Desc.': 'Method for counting records. Options: "single": Count '
                         'each record as a single entry; "reads": Use the number of '
                         'reads per hyb record as the count (may contain PCR '
                         'duplicates); "records": Count the number of records '
                         'represented by each hyb record entry (1 for "unmerged" '
                         'records, >= 1 for "merged" records)'}
}
```

hybkit.settings.**HybRecord_settings = {'allow_undefined_flags': False, 'allow_unknown_seg_types': False, 'custom_flags': [], 'hyb_placeholder': '.', 'mirna_types': ['miRNA', 'microRNA'], 'reorder_flags': True}**

Settings for *HybRecord* (page 9), created from *HybRecord_settings_info* (page 52)

hybkit.settings.**HybFile_settings = {'hybformat_id': False, 'hybformat_ref': False}**

Settings for *HybFile* (page 21), created from *HybFile_settings_info* (page 52)

hybkit.settings.**FoldRecord_settings = {'allowed_mismatches': 0, 'error_mode': 'raise', 'fold_placeholder': '.', 'seq_type': 'static'}**

Settings for *FoldRecord* (page 23), created from *FoldRecord_settings_info* (page 53)

hybkit.settings.**FoldFile_settings = {}**

Settings for FoldFile, created from *FoldFile_settings_info* (page 54)

hybkit.settings.**HybFoldIter_settings = {'error_checks': ['hybrecord_indel', 'foldrecord_nofold', 'max_mismatch', 'energy_mismatch'], 'iter_error_mode': 'warn_skip', 'max_sequential_skips': 100}**

Settings for *HybFoldIter* (page 33), created from *HybFoldIter_settings_info* (page 54)

hybkit.settings.**Analysis_settings = {'out_delim': ',', 'quant_mode': 'single'}**

Settings for BaseAnalysis, created from *Analysis_settings_info* (page 55)

2.6 hybkit.util

This module contains helper functions for hybkit's command line scripts.

hybkit.util.**get_argparse_doc**(*docstring: str*[737]) → str[738]

Get the argparse description from a docstring.

Parameters
docstring (*str*[739]) -- A docstring.

Returns
A string containing the argparse description.

hybkit.util.**dir_exists**(*dir_name: str*[740]) → str[741]

Check if a directory exists at the provided path (else raise), and return a normalized path.

Parameters
dir_name (*str*[742]) -- Name of directory to check for existence.

Returns
A normalized version of the path passed to dir_name.

hybkit.util.**file_exists**(*file_name: str*[743], *required_suffixes: Optional*[744]*[List*[745]*[str*[746]*]] = None*) → str[747]

Check if a file exists at the provided path, and return a normalized path.

Parameters

- **file_name** (*str*[748]) -- Name of file to check for existence.
- **required_suffixes** (*list*[749]*, optional*) -- List of strings containing file-name suffixes. If provided, a file passed to file-exists must end with one of the strings provided. Otherwise an error will be raised.

Returns
A normalized version of the path passed to file_name.

[737] https://docs.python.org/3/library/stdtypes.html#str
[738] https://docs.python.org/3/library/stdtypes.html#str
[739] https://docs.python.org/3/library/stdtypes.html#str
[740] https://docs.python.org/3/library/stdtypes.html#str
[741] https://docs.python.org/3/library/stdtypes.html#str
[742] https://docs.python.org/3/library/stdtypes.html#str
[743] https://docs.python.org/3/library/stdtypes.html#str
[744] https://docs.python.org/3/library/typing.html#typing.Optional
[745] https://docs.python.org/3/library/typing.html#typing.List
[746] https://docs.python.org/3/library/stdtypes.html#str
[747] https://docs.python.org/3/library/stdtypes.html#str
[748] https://docs.python.org/3/library/stdtypes.html#str
[749] https://docs.python.org/3/library/stdtypes.html#list

`hybkit.util.`**`hyb_exists`**(*file_name: str*[750]) → str[751]

Check if a .hyb file exists at the provided path, and return a normalized path.

Wrapper for *file_exists()* (page 56) that includes the required suffixes in *hybkit.settings.HYB_SUFFIXES* (page 51).

Parameters
file_name (*str*[752]) -- Name of file to check for existence.

Returns
A normalized version of the path passed to file_name.

`hybkit.util.`**`vienna_exists`**(*file_name: str*[753]) → str[754]

Check if a .vienna file exists at the provided path, and return a normalized path.

Wrapper for *file_exists()* (page 56) that includes the required suffixes in *hybkit.settings.VIENNA_SUFFIXES* (page 51).

Parameters
file_name (*str*[755]) -- Name of file to check for existence.

Returns
A normalized version of the path passed to file_name.

`hybkit.util.`**`ct_exists`**(*file_name: str*[756]) → str[757]

Check if a .ct file exists at the provided path, and return a normalized path.

Wrapper for *file_exists()* (page 56) that includes the required suffixes in *hybkit.settings.CT_SUFFIXES* (page 51).

Parameters
file_name (*str*[758]) -- Name of file to check for existence.

Returns
A normalized version of the path passed to file_name.

`hybkit.util.`**`fold_exists`**(*file_name: str*[759]) → str[760]

Check if a fold-representing file exists at the provided path, and return a normalized path.

Wrapper for *file_exists()* (page 56) that includes the required suffixes in *hybkit.settings.FOLD_SUFFIXES* (page 52).

Parameters
file_name (*str*[761]) -- Name of file to check for existence.

Returns
A normalized version of the path passed to file_name.

`hybkit.util.`**`out_path_exists`**(*file_name: str*[762]) → str[763]

Check if the directory of the specified output path exists, and return a normalized path.

[750] https://docs.python.org/3/library/stdtypes.html#str
[751] https://docs.python.org/3/library/stdtypes.html#str
[752] https://docs.python.org/3/library/stdtypes.html#str
[753] https://docs.python.org/3/library/stdtypes.html#str
[754] https://docs.python.org/3/library/stdtypes.html#str
[755] https://docs.python.org/3/library/stdtypes.html#str
[756] https://docs.python.org/3/library/stdtypes.html#str
[757] https://docs.python.org/3/library/stdtypes.html#str
[758] https://docs.python.org/3/library/stdtypes.html#str
[759] https://docs.python.org/3/library/stdtypes.html#str
[760] https://docs.python.org/3/library/stdtypes.html#str
[761] https://docs.python.org/3/library/stdtypes.html#str

Parameters

file_name (*str*[764]) -- Name of path to an output file to check.

Returns

A normalized version of the path passed to file_name.

hybkit.util.**make_out_file_name**(*in_file_name: str*[765], *name_suffix: str*[766] *= 'out'*, *in_suffix: str*[767] *= ''*, *out_suffix: str*[768] *= ''*, *out_dir: str*[769] *= ''*, *seg_sep: str*[770] *= '_'*) → str[771]

Given an input file name, generate an output file name.

Parameters

- **in_file_name** (*str*[772]) -- Name of input file as template.
- **name_suffix** (*str*[773]) -- Suffix to add to name before file type.
- **in_suffix** (*str*[774]) -- File type suffix on in_file_name (to remove).
- **out_suffix** (*str*[775]) -- File type suffix to add to final output file.
- **out_dir** (*str*[776]) -- Directory path in which to place output file.
- **seg_sep** (*str*[777]) -- Separator string between file name segments.

Returns

An output file path based on the input file template.

hybkit.util.**validate_args**(*args: Namespace*[778], *parser: Optional*[779]*[ArgumentParser*[780]*] = None*) → bool[781]

Check supplied arguments to make sure there are no hidden contradictions.

Current checks:

- If explicit output file names supplied, be sure that they match the number of input files provided.
- If fold files provided, make sure that they match the number of input hyb files provided.

Parameters

- **args** (*argparse.Namespace*[782]) -- The arguments produced by argparse.
- **parser** (*argparse.ArgumentParser*[783]*, optional*) -- Argparse parser object to use for verbose outputting of help message.

[762] https://docs.python.org/3/library/stdtypes.html#str
[763] https://docs.python.org/3/library/stdtypes.html#str
[764] https://docs.python.org/3/library/stdtypes.html#str
[765] https://docs.python.org/3/library/stdtypes.html#str
[766] https://docs.python.org/3/library/stdtypes.html#str
[767] https://docs.python.org/3/library/stdtypes.html#str
[768] https://docs.python.org/3/library/stdtypes.html#str
[769] https://docs.python.org/3/library/stdtypes.html#str
[770] https://docs.python.org/3/library/stdtypes.html#str
[771] https://docs.python.org/3/library/stdtypes.html#str
[772] https://docs.python.org/3/library/stdtypes.html#str
[773] https://docs.python.org/3/library/stdtypes.html#str
[774] https://docs.python.org/3/library/stdtypes.html#str
[775] https://docs.python.org/3/library/stdtypes.html#str
[776] https://docs.python.org/3/library/stdtypes.html#str
[777] https://docs.python.org/3/library/stdtypes.html#str
[778] https://docs.python.org/3/library/argparse.html#argparse.Namespace
[779] https://docs.python.org/3/library/typing.html#typing.Optional
[780] https://docs.python.org/3/library/argparse.html#argparse.ArgumentParser
[781] https://docs.python.org/3/library/functions.html#bool
[782] https://docs.python.org/3/library/argparse.html#argparse.Namespace
[783] https://docs.python.org/3/library/argparse.html#argparse.ArgumentParser

`hybkit.util.validate_args_exit`(*args: Namespace*[784], *parser: Optional*[785]*[ArgumentParser*[786]*] = None*) → None[787]

Check supplied arguments using `validate_args()` (page 58), and exit if a conflict exists.

Parameters

- **args** (*argparse.Namespace*[788]) -- The arguments produced by argparse.
- **parser** (*argparse.ArgumentParser*[789]*, optional*) -- Argparse parser object to use for verbose outputting of help message.

`hybkit.util.set_setting`(*setting: str*[790], *set_value: Any*[791], *verbose: bool*[792] *= False*) → str[793]

Take a namespace object as from an argparse parser and update settings.

Each setting in the following settings dictionaries are checked and set where applicable:

`HybRecord` (page 9) Settings	`hybkit.settings.HybRecord_settings` (page 55)
`HybFile` (page 21) Settings	`hybkit.settings.HybFile_settings` (page 55)
`FoldRecord` (page 23) Settings	`hybkit.settings.FoldRecord_settings` (page 55)
`FoldFile` Settings	`hybkit.settings.FoldFile_settings` (page 55)
`HybFoldIter` (page 33) Settings	`hybkit.settings.HybFoldIter_settings` (page 56)
`Analysis` Settings	`hybkit.settings.Analysis_settings` (page 56)

Parameters

- **setting** (*str*[794]) -- Name of setting to change
- **set_value** (*str*[795]) -- New value for setting
- **verbose** (bool[796], optional) -- If True, print when changing setting.

`hybkit.util.set_settings_from_namespace`(*nspace: Namespace*[797], *verbose: bool*[798] *= False*) → None[799]

Take a namespace object as from an argparse parser and update settings.

See `set_setting()` (page 59) for details

Parameters

- **nspace** (*argparse.Namespace*[800]) -- Namespace containing settings
- **verbose** (bool[801], optional) -- If True, print when changing setting.

[784] https://docs.python.org/3/library/argparse.html#argparse.Namespace
[785] https://docs.python.org/3/library/typing.html#typing.Optional
[786] https://docs.python.org/3/library/argparse.html#argparse.ArgumentParser
[787] https://docs.python.org/3/library/constants.html#None
[788] https://docs.python.org/3/library/argparse.html#argparse.Namespace
[789] https://docs.python.org/3/library/argparse.html#argparse.ArgumentParser
[790] https://docs.python.org/3/library/stdtypes.html#str
[791] https://docs.python.org/3/library/typing.html#typing.Any
[792] https://docs.python.org/3/library/functions.html#bool
[793] https://docs.python.org/3/library/stdtypes.html#str
[794] https://docs.python.org/3/library/stdtypes.html#str
[795] https://docs.python.org/3/library/stdtypes.html#str
[796] https://docs.python.org/3/library/functions.html#bool
[797] https://docs.python.org/3/library/argparse.html#argparse.Namespace
[798] https://docs.python.org/3/library/functions.html#bool
[799] https://docs.python.org/3/library/constants.html#None
[800] https://docs.python.org/3/library/argparse.html#argparse.Namespace
[801] https://docs.python.org/3/library/functions.html#bool

2.7 hybkit.errors

Module storing hybkit error classes.

exception `hybkit.errors.`**`HybkitError`**

Base class for Hybkit errors.

Variables

message (*str*[802]) -- Human-readable string describing the error.

exception `hybkit.errors.`**`HybkitArgError`**

Error raised when an invalid argument is provided to a Hybkit function.

Subclass of *HybkitError* (page 60).

Variables

message (*str*[803]) -- Human-readable string describing the error.

exception `hybkit.errors.`**`HybkitConstructorError`**

Error raised when a read error occurs.

Subclass of *HybkitError* (page 60).

Variables

message (*str*[804]) -- Human-readable string describing the error.

exception `hybkit.errors.`**`HybkitIterError`**

Error raised when an error is encountered during Hybkit iteration.

Subclass of *HybkitError* (page 60).

Variables

message (*str*[805]) -- Human-readable string describing the error.

exception `hybkit.errors.`**`HybkitMiscError`**

Error raised when an error is encountered during Hybkit usage.

Subclass of *HybkitError* (page 60).

Variables

message (*str*[806]) -- Human-readable string describing the error.

[802] https://docs.python.org/3/library/stdtypes.html#str
[803] https://docs.python.org/3/library/stdtypes.html#str
[804] https://docs.python.org/3/library/stdtypes.html#str
[805] https://docs.python.org/3/library/stdtypes.html#str
[806] https://docs.python.org/3/library/stdtypes.html#str

CHAPTER

THREE

HYBKIT TOOLKIT

The hybkit toolkit contains command-line scripts for analysis and manipulation of hyb and fold files. Scripts are implemented in Python3, and the hybkit module must be on the user's PYTHONPATH for script execution.

The command-line options and flags are generated with the Python3 argparse module. Relevant settings pertaining to specific hybkit classes are accessible via command-line flags, as demonstrated in the "shell_analysis" implementations in the *Example Analyses* (page 82).

This version of hybkit includes the following executables:

Utility	Description
hyb_check (page 61)	Parse a hyb (/fold) file and check for errors
hyb_eval (page 71)	Evaluate hyb (/fold) records to identify segment types and miRNAs
hyb_filter (page 65)	Filter a hyb (/fold) file to a specific subset of sequences
hyb_analyze (page 77)	Perform a type, miRNA, summary, or target analysis on a hyb (/fold) file

Detailed descriptions and usage information are available at each respective script page.

3.1 hyb_check

Read one or more hyb (and optional fold) format files and check for errors.

This utility reads in one or more files in hyb-format (see the *hybkit Hyb File Specification* (page 5)) and uses hybkit's internal file error checking to check for errors. This can be useful as an initial preparation step for further analyses.

Example system calls:

```
hyb_check -i my_file_1.hyb -f my_file_1.vienna
hyb_check -i my_file_1.hyb my_file_2.hyb -f my_file_1.vienna \\
    my_file_2.vienna -v --custom_flags myflag
```

```
usage: hyb_check [-h] -i PATH_TO/MY_FILE.HYB [PATH_TO/MY_FILE.HYB ...]
                 [-f [PATH_TO/MY_FILE.VIENNA [PATH_TO/MY_FILE.VIENNA ...]]]
                 [--version] [-v | -s]
                 [--mirna_types MIRNA_TYPES [MIRNA_TYPES ...]]
                 [--custom_flags CUSTOM_FLAGS [CUSTOM_FLAGS ...]]
                 [--hyb_placeholder HYB_PLACEHOLDER]
                 [--reorder_flags {True,False}]
```

(continues on next page)

(continued from previous page)

```
                 [--allow_undefined_flags [{True,False}]]
                 [--allow_unknown_seg_types [{True,False}]]
                 [--hybformat_id [{True,False}]]
                 [--hybformat_ref [{True,False}]]
                 [--allowed_mismatches ALLOWED_MISMATCHES]
                 [--fold_placeholder FOLD_PLACEHOLDER] [-y {static,dynamic}]
                 [--error_mode {raise,warn_return,return}]
                 [--error_checks {hybrecord_indel,foldrecord_nofold,max_mismatch,energy_
↪mismatch}]
                 [--iter_error_mode {raise,warn_return,warn_skip,skip,return}]
                 [--max_sequential_skips MAX_SEQUENTIAL_SKIPS]
```

3.1.1 Named Arguments

-i, --in_hyb REQUIRED path to one or more hyb-format files with a ".hyb" suffix for use in the evaluation.

-f, --in_fold REQUIRED path to one or more RNA secondary-structure files with a ".vienna" or ".ct" suffix for use in the evaluation.

--version Print version and exit.

-v, --verbose Print verbose output during run.

Default: False

-s, --silent Print no output during run.

Default: False

3.1.2 Hyb Record Settings

--mirna_types "seg_type" fields identifying a miRNA

Default: ['miRNA', 'microRNA']

--custom_flags Custom flags to allow in addition to those specified in the hybkit specification.

Default: []

--hyb_placeholder placeholder character/string for missing data in hyb files.

Default: "."

--reorder_flags Possible choices: True, False

Re-order flags to the hybkit-specification order when writing hyb records.

Default: True

--allow_undefined_flags Possible choices: True, False

Allow use of flags not defined in the hybkit-specification order when reading and writing hyb records. As the preferred alternative to using this setting, the --custom_flags argument can be be used to supply custom allowed flags.

Default: False

--allow_unknown_seg_types Possible choices: True, False

Allow unknown segment types when assigning segment types.

Default: False

3.1.3 Hyb File Settings

--hybformat_id Possible choices: True, False

The Hyb Software Package places further information in the "id" field of the hybrid record that can be used to infer the number of contained read counts. When set to True, the identifiers will be parsed as: "<read_id>_<read_count>"

Default: False

--hybformat_ref Possible choices: True, False

The Hyb Software Package uses a reference database with identifiers that contain sequence type and other sequence information. When set to True, all hyb file identifiers will be parsed as: "<gene_id>_<transcript_id>_<gene_name>_<seg_type>"

Default: False

3.1.4 Fold Record Settings

--allowed_mismatches For DynamicFoldRecords, allowed number of mismatches with a HybRecord.

Default: 0

--fold_placeholder Placeholder character/string for missing data for reading/writing fold records.

Default: "."

-y, --seq_type Possible choices: static, dynamic

Type of fold record object to use. Options: "static": FoldRecord, requires an exact sequence match to be paired with a HybRecord; "dynamic": DynamicFoldRecord, requires a sequence match to the "dynamic" annotated regions of a HybRecord, and may be shorter/longer than the original sequence.

Default: "static"

--error_mode Possible choices: raise, warn_return, return

Mode for handling errors during reading of HybFiles (overridden by HybFoldIter.settings['iter_error_mode'] when using HybFoldIter). Options: "raise": Raise an error when encountered and exit program ; "warn_return": Print a warning and return the error_value ; "return": Return the error value with no program output. record is encountered.

Default: "raise"

3.1.5 Hyb-Fold Iterator Settings

--error_checks Possible choices: hybrecord_indel, foldrecord_nofold, max_mismatch, energy_mismatch

Error checks for simultaneous HybFile and FoldFile parsing. Options: "hybrecord_indel": Error for HybRecord objects where one/both sequences have insertions/deletions in alignment, which prevents matching of sequences; "foldrecord_nofold": Error when failure in reading a fold_record object; "max_mismatch": Error when mismatch between hybrecord and foldrecord sequences is greater than FoldRecord "allowed_mismatches" setting; "energy_mismatch": Error when a mismatch exists between HybRecord and FoldRecord energy values.

Default: ['hybrecord_indel', 'foldrecord_nofold', 'max_mismatch', 'energy_mismatch']

--iter_error_mode Possible choices: raise, warn_return, warn_skip, skip, return

Mode for handling errors found during error checks. Overrides HybRecord "error_mode" setting when using HybFoldIter. Options: "raise": Raise an error when encountered; "warn_return": Print a warning and return the value; "warn_skip": Print a warning and continue to the next iteration; "skip": Continue to the next iteration without any output; "return": return the value without any error output;

Default: "warn_skip"

--max_sequential_skips Maximum number of record(-pairs) to skip in a row. Limited as several sequential skips usually indicates an issue with record formatting or a desynchronization between files.

Default: 100

Output File Naming:

Output files can be named in two fashions: via automatic name generation, or by providing specific out file names.

Automatic Name Generation:

For output name generation, the default respective naming scheme is used:

```
hyb_script -i PATH_TO/MY_FILE_1.HYB [...]
    -->  OUT_DIR/MY_FILE_1_ADDSUFFIX.HYB
```

This output file path can be modified with the arguments {--out_dir, --out_suffix} described below.

The output directory defaults to the current working directory (`$PWD`), and can be modified with the `--out_dir <dir>` argument. Note: The provided directory must exist, or an error will be raised. For Example:

```
hyb_script -i PATH_TO/MY_FILE_1.HYB [...] --out_dir MY_OUT_DIR
    -->  MY_OUT_DIR/MY_FILE_1_ADDSUFFIX.HYB
```

The suffix used for output files is based on the primary actions of the script. It can be specified using `--out_suffix <suffix>`. This can optionally include the ".hyb" final suffix. for Example:

```
hyb_script -i PATH_TO/MY_FILE_1.HYB [...] --out_suffix MY_SUFFIX
    -->  OUT_DIR/MY_FILE_1_MY_SUFFIX.HYB
#OR
hyb_script -i PATH_TO/MY_FILE_1.HYB [...] --out_suffix MY_SUFFIX.HYB
    -->  OUT_DIR/MY_FILE_1_MY_SUFFIX.HYB
```

Specific Output Names:
Alternatively, specific file names can be provided via the -o/--out_hyb argument, ensuring that the same number of input and output files are provided. This argument takes precedence over all automatic output file naming options (--out_dir, --out_suffix), which are ignored if -o/--out_hyb is provided. For Example:

```
hyb_script [...] --out_hyb MY_OUT_DIR/OUT_FILE_1.HYB MY_OUT_DIR/OUT_FILE_2.HYB
    -->  MY_OUT_DIR/OUT_FILE_1.hyb
    -->  MY_OUT_DIR/OUT_FILE_2.hyb
```

Note: The directory provided with output file paths (MY_OUT_DIR above) must exist, otherwise an error will be raised.

3.2 hyb_filter

Filter hyb (and corresponding fold) files to meet (or exclude) specific criteria.

This script takes one or more filter and/or exclusion criteria and outputs only those records matching (/excluding) those criteria.

The filter criteria and options are based on the options provided by the *hybkit.HybRecord.prop()* (page 16) method of the Hybkit API. For more information see the full documentation for the *HybRecord* (page 9) class.

Example System Calls:

```
hyb_filter -i my_file_1.hyb --filter has_seg_types
# Outputs records that have completed a segtype analysis

hyb_filter -i my_file_1.hyb -f my_file_1.vienna \\
    --include seg_type mRNA
# Outputs hyb and fold records where hyb record has either segtype of mRNA

hyb_filter -i my_file_1.hyb --exclude seg_type mRNA
# Outputs records without either segtype of mRNA

hyb_filter -i my_file_1.hyb --include seg1_type mRNA
# Outputs records with only the first / 5p segtype of mRNA

hyb_filter -i my_file_1.hyb my_file_2.hyb -f my_file_1.vienna my_file_2.vienna \\
    --include seg_type_contains RNA
# Outputs all records with a segtype that includes
#   the string "RNA" (case-sensitive)

hyb_filter -i my_file_1.hyb --filter seg_contains kshv
# Outputs records where either segment identifier contains the
#   the string: "kshv" (case-sensitive)
```

Multiple filtering options can be used together. The `-m` / `--filter_mode` argument determines whether "any" (DEFAULT) or "all" filters are required to be true for inclusion. Note: Matching any exclusion criteria results in exclusion

of the record.

Example System Calls (match ALL criteria):

```
hyb_filter -i my_file_1.hyb -f my_file_1.vienna \\
           --filter seg_contains kshv \\
           --filter_2 seg_type miRNA
# Outputs records with either reference sequence identifier containing "kshv"
#   and with either segment having an assigned segtype of miRNA
```

Example System Calls (match ANY criteria):

```
hyb_filter -i my_file_1.hyb --filter_mode any \\
           --filter seg_type miRNA \\
           --filter_2 seg_type lncRNA
# Outputs records containing either segment type matching
#   either "miRNA" or "lncRNA" (case-sensitive)
```

```
usage: hyb_filter [-h] -i PATH_TO/MY_FILE.HYB [PATH_TO/MY_FILE.HYB ...]
                  [-f [PATH_TO/MY_FILE.VIENNA [PATH_TO/MY_FILE.VIENNA ...]]]
                  [-o PATH_TO/OUT_FILE.HYB [PATH_TO/OUT_FILE.HYB ...]]
                  [-l PATH_TO/OUT_FILE.VIENNA [PATH_TO/OUT_FILE.VIENNA ...]]
                  [-d OUT_DIR] [-u OUT_SUFFIX] [-m {any,all}]
                  [--skip_dup_id_before] [--skip_dup_id_after]
                  [--filter FILTER [FILTER ...]]
                  [--filter_2 FILTER_2 [FILTER_2 ...]]
                  [--filter_3 FILTER_3 [FILTER_3 ...]]
                  [--exclude EXCLUDE [EXCLUDE ...]]
                  [--exclude_2 EXCLUDE_2 [EXCLUDE_2 ...]]
                  [--exclude_3 EXCLUDE_3 [EXCLUDE_3 ...]] [--set_dataset]
                  [--version] [-v | -s]
                  [--mirna_types MIRNA_TYPES [MIRNA_TYPES ...]]
                  [--custom_flags CUSTOM_FLAGS [CUSTOM_FLAGS ...]]
                  [--hyb_placeholder HYB_PLACEHOLDER]
                  [--reorder_flags {True,False}]
                  [--allow_undefined_flags [{True,False}]]
                  [--allow_unknown_seg_types [{True,False}]]
                  [--hybformat_id [{True,False}]]
                  [--hybformat_ref [{True,False}]]
                  [--allowed_mismatches ALLOWED_MISMATCHES]
                  [--fold_placeholder FOLD_PLACEHOLDER] [-y {static,dynamic}]
                  [--error_mode {raise,warn_return,return}]
                  [--error_checks {hybrecord_indel,foldrecord_nofold,max_mismatch,energy_
↪mismatch}]
                  [--iter_error_mode {raise,warn_return,warn_skip,skip,return}]
                  [--max_sequential_skips MAX_SEQUENTIAL_SKIPS]
```

3.2.1 Named Arguments

-i, --in_hyb
REQUIRED path to one or more hyb-format files with a ".hyb" suffix for use in the evaluation.

-f, --in_fold
REQUIRED path to one or more RNA secondary-structure files with a ".vienna" or ".ct" suffix for use in the evaluation.

-o, --out_hyb
Optional path to one or more hyb-format file for output (should include a ".hyb" suffix). If not provided, the output for input file "PATH_TO/MY_FILE.HYB" will be used as a template for the output "OUT_DIR/MY_FILE_OUT.HYB".

-l, --out_fold
Optional path to one or more ".vienna" or ".ct"-format files for output (should include appropriate ".vienna"/".ct" suffix). If not provided, the output for input file "PATH_TO/MY_FILE.VIENNA" will be used as a template for the output "OUT_DIR/MY_FILE_OUT.VIENNA".

-d, --out_dir
Path to directory for output of files. Defaults to the current working directory.

Default: $PWD

-u, --out_suffix
Suffix to add to the name of output files, before any file- or analysis-specific suffixes. The file-type appropriate suffix will be added automatically.

Default: "_filtered"

-m, --filter_mode
Possible choices: any, all

Modes for evaluating multiple filters. The "all" mode requires all provided filters to be true for inclusion. The "any" mode requires only one provided filter to be true for inclusion. (Note: matching any exclusion filter is grounds for exclusion of record.)

Default: "all"

--skip_dup_id_before
Skip sequential duplicate read IDs before filtering.

Default: False

--skip_dup_id_after
Skip sequential duplicate read IDs after filtering.

Default: False

--filter
Filter criteria #1. Records matching the criteria will be included in output. Includes a filter type, Ex: "seg_name_contains", and an argument, Ex: "ENST00000340384". (Note: not all filter types require a second argument, for Example: "has_mirna_seg")

--filter_2
Filter criteria #2. Records matching the criteria will be included in output. Includes a filter type, Ex: "seg_name_contains", and an argument, Ex: "ENST00000340384". (Note: not all filter types require a second argument, for Example: "has_mirna_seg")

--filter_3
Filter criteria #3. Records matching the criteria will be included in output. Includes a filter type, Ex: "seg_name_contains", and an argument, Ex: "ENST00000340384". (Note: not all filter types require a second argument, for Example: "has_mirna_seg")

--exclude
Exclusion filter criteria #1. Records matching the criteria will be excluded from output. Includes a filter type, Ex: "seg_name_contains",

and an argument, Ex: "ENST00000340384". (Note: not all filter types require a second argument, for Example: "has_mirna_seg")

--exclude_2 Exclusion filter criteria #2. Records matching the criteria will be excluded from output. Includes a filter type, Ex: "seg_name_contains", and an argument, Ex: "ENST00000340384". (Note: not all filter types require a second argument, for Example: "has_mirna_seg")

--exclude_3 Exclusion filter criteria #3. Records matching the criteria will be excluded from output. Includes a filter type, Ex: "seg_name_contains", and an argument, Ex: "ENST00000340384". (Note: not all filter types require a second argument, for Example: "has_mirna_seg")

--set_dataset Set "dataset" flag to value of the input file name.

Default: False

--version Print version and exit.

-v, --verbose Print verbose output during run.

Default: False

-s, --silent Print no output during run.

Default: False

3.2.2 Hyb Record Settings

--mirna_types "seg_type" fields identifying a miRNA

Default: ['miRNA', 'microRNA']

--custom_flags Custom flags to allow in addition to those specified in the hybkit specification.

Default: []

--hyb_placeholder placeholder character/string for missing data in hyb files.

Default: "."

--reorder_flags Possible choices: True, False

Re-order flags to the hybkit-specification order when writing hyb records.

Default: True

--allow_undefined_flags Possible choices: True, False

Allow use of flags not defined in the hybkit-specification order when reading and writing hyb records. As the preferred alternative to using this setting, the --custom_flags argument can be be used to supply custom allowed flags.

Default: False

--allow_unknown_seg_types Possible choices: True, False

Allow unknown segment types when assigning segment types.

Default: False

3.2.3 Hyb File Settings

--hybformat_id Possible choices: True, False

The Hyb Software Package places further information in the "id" field of the hybrid record that can be used to infer the number of contained read counts. When set to True, the identifiers will be parsed as: "<read_id>_<read_count>"

Default: False

--hybformat_ref Possible choices: True, False

The Hyb Software Package uses a reference database with identifiers that contain sequence type and other sequence information. When set to True, all hyb file identifiers will be parsed as: "<gene_id>_<transcript_id>_<gene_name>_<seg_type>"

Default: False

3.2.4 Fold Record Settings

--allowed_mismatches For DynamicFoldRecords, allowed number of mismatches with a HybRecord.

Default: 0

--fold_placeholder Placeholder character/string for missing data for reading/writing fold records.

Default: "."

-y, --seq_type Possible choices: static, dynamic

Type of fold record object to use. Options: "static": FoldRecord, requires an exact sequence match to be paired with a HybRecord; "dynamic": DynamicFoldRecord, requires a sequence match to the "dynamic" annotated regions of a HybRecord, and may be shorter/longer than the original sequence.

Default: "static"

--error_mode Possible choices: raise, warn_return, return

Mode for handling errors during reading of HybFiles (overridden by HybFoldIter.settings['iter_error_mode'] when using HybFoldIter). Options: "raise": Raise an error when encountered and exit program ; "warn_return": Print a warning and return the error_value ; "return": Return the error value with no program output. record is encountered.

Default: "raise"

3.2.5 Hyb-Fold Iterator Settings

--error_checks Possible choices: hybrecord_indel, foldrecord_nofold, max_mismatch, energy_mismatch

Error checks for simultaneous HybFile and FoldFile parsing. Options: "hybrecord_indel": Error for HybRecord objects where one/both sequences have insertions/deletions in alignment, which prevents matching of sequences; "foldrecord_nofold": Error when failure in reading a fold_record object; "max_mismatch": Error when mismatch between hybrecord and foldrecord sequences is greater than FoldRecord "allowed_mismatches" setting; "energy_mismatch": Error when a mismatch exists between HybRecord and FoldRecord energy values.

Default: ['hybrecord_indel', 'foldrecord_nofold', 'max_mismatch', 'energy_mismatch']

--iter_error_mode Possible choices: raise, warn_return, warn_skip, skip, return

Mode for handling errors found during error checks. Overrides HybRecord "error_mode" setting when using HybFoldIter. Options: "raise": Raise an error when encountered; "warn_return": Print a warning and return the value; "warn_skip": Print a warning and continue to the next iteration; "skip": Continue to the next iteration without any output; "return": return the value without any error output;

Default: "warn_skip"

--max_sequential_skips Maximum number of record(-pairs) to skip in a row. Limited as several sequential skips usually indicates an issue with record formatting or a desynchronization between files.

Default: 100

Output File Naming:

Output files can be named in two fashions: via automatic name generation, or by providing specific out file names.

Automatic Name Generation:

For output name generation, the default respective naming scheme is used:

```
hyb_script -i PATH_TO/MY_FILE_1.HYB [...]
    -->  OUT_DIR/MY_FILE_1_ADDSUFFIX.HYB
```

This output file path can be modified with the arguments {--out_dir, --out_suffix} described below.

The output directory defaults to the current working directory (`$PWD`), and can be modified with the `--out_dir` <dir> argument. Note: The provided directory must exist, or an error will be raised. For Example:

```
hyb_script -i PATH_TO/MY_FILE_1.HYB [...] --out_dir MY_OUT_DIR
    -->  MY_OUT_DIR/MY_FILE_1_ADDSUFFIX.HYB
```

The suffix used for output files is based on the primary actions of the script. It can be specified using `--out_suffix <suffix>`. This can optionally include the ".hyb" final suffix. for Example:

```
hyb_script -i PATH_TO/MY_FILE_1.HYB [...] --out_suffix MY_SUFFIX
    -->  OUT_DIR/MY_FILE_1_MY_SUFFIX.HYB
#OR
hyb_script -i PATH_TO/MY_FILE_1.HYB [...] --out_suffix MY_SUFFIX.HYB
    -->  OUT_DIR/MY_FILE_1_MY_SUFFIX.HYB
```

Specific Output Names:

Alternatively, specific file names can be provided via the -o/--out_hyb argument, ensuring that the same number of input and output files are provided. This argument takes precedence over all automatic output file naming options (--out_dir, --out_suffix), which are ignored if -o/--out_hyb is provided. For Example:

```
hyb_script [...] --out_hyb MY_OUT_DIR/OUT_FILE_1.HYB MY_OUT_DIR/OUT_FILE_2.HYB
    -->  MY_OUT_DIR/OUT_FILE_1.hyb
    -->  MY_OUT_DIR/OUT_FILE_2.hyb
```

Note: The directory provided with output file paths (MY_OUT_DIR above) must exist, otherwise an error will be raised.

3.3 hyb_eval

Read hyb files (and optional matched fold files) and evaluate the contained hybrids.

This utility reads in one or more files in hyb-format (see the *hybkit Hyb File Specification* (page 5)) and corresponding fold files (.vienna or .ct) and evaluates hybrid record properties.

Evaluation Types:

`type`	Assigns types to each segment within hyb records
`mirna`	Assigns which segments are a miRNA based on segment types.

`type` Evaluation:

The 'type' evaluation utilizes the `hybkit.HybRecord.eval_types()` (page 14) method to assign the record flags: *seg1_type* (page 6) and *seg2_type* (page 6)

Example system calls:

```
$ hyb_eval -t type -i my_file_1.hyb

$ hyb_eval -t type -i my_file_1.hyb -f my_file_1.vienna

$ hyb_eval -t type \\
            -i my_file_1.hyb my_file_2.hyb \\
            -f my_file_1.vienna my_file_2.vienna \\
            --type_method string_match \\
            --type_parameters my_parameters_file.csv \\
            --allow_unknown_seg_types
```

`mirna` Evaluation:

The 'mirna' evaluation uses the `hybkit.HybRecord.eval_mirna()` (page 15) method to identify properties relating to mirna within the hybrids, including mirna presence and positions. This evaluation requires the seg_type flags to be filled, either by a type evaluation, or by parsing the read using the `--hybformat_ref`

True option with a hyb-format reference. The *mirna_seg* (page 6) flag is then set for each record, indicating the presence and position of any miRNA within the hybrid.

Example system calls:

```
$ hyb_eval -t mirna -i my_file_1.hyb

$ hyb_eval -t mirna -i my_file_1.hyb -f my_file_1.vienna

$ hyb_eval -t mirna -i my_file_1.hyb my_vile_2.hyb \\
            -f my_file_1.vienna my_file_2.vienna \\
            --mirna_types miRNA kshv-miRNA
```

This can also be combined with the type evaluation, as such:

```
$ hyb_eval -t type mirna -i my_file_1.hyb \\
            --type_method string_match \\
            --type_parameters my_parameters_file.csv \\
            --allow_unknown_seg_types \\
            --mirna_types miRNA kshv-miRNA
```

```
usage: hyb_analysis [-h] -i PATH_TO/MY_FILE.HYB [PATH_TO/MY_FILE.HYB ...]
                    [-f [PATH_TO/MY_FILE.VIENNA [PATH_TO/MY_FILE.VIENNA ...]]]
                    [-o PATH_TO/OUT_FILE.HYB [PATH_TO/OUT_FILE.HYB ...]]
                    [-l PATH_TO/OUT_FILE.VIENNA [PATH_TO/OUT_FILE.VIENNA ...]]
                    [-d OUT_DIR] [-u OUT_SUFFIX]
                    [-t {type,mirna} [{type,mirna} ...]]
                    [--type_method {hybformat,string_match,id_map}]
                    [--type_params_file PATH_TO/PARAMETERS_FILE]
                    [--set_dataset] [--version] [-v | -s]
                    [--mirna_types MIRNA_TYPES [MIRNA_TYPES ...]]
                    [--custom_flags CUSTOM_FLAGS [CUSTOM_FLAGS ...]]
                    [--hyb_placeholder HYB_PLACEHOLDER]
                    [--reorder_flags {True,False}]
                    [--allow_undefined_flags [{True,False}]]
                    [--allow_unknown_seg_types [{True,False}]]
                    [--hybformat_id [{True,False}]]
                    [--hybformat_ref [{True,False}]]
                    [--allowed_mismatches ALLOWED_MISMATCHES]
                    [--fold_placeholder FOLD_PLACEHOLDER]
                    [-y {static,dynamic}]
                    [--error_mode {raise,warn_return,return}]
                    [--error_checks {hybrecord_indel,foldrecord_nofold,max_mismatch,
↪energy_mismatch}]
                    [--iter_error_mode {raise,warn_return,warn_skip,skip,return}]
                    [--max_sequential_skips MAX_SEQUENTIAL_SKIPS]
```

3.3.1 Named Arguments

-i, --in_hyb REQUIRED path to one or more hyb-format files with a ".hyb" suffix for use in the evaluation.

-f, --in_fold REQUIRED path to one or more RNA secondary-structure files with a ".vienna" or ".ct" suffix for use in the evaluation.

-o, --out_hyb Optional path to one or more hyb-format file for output (should include a ".hyb" suffix). If not provided, the output for input file "PATH_TO/MY_FILE.HYB" will be used as a template for the output "OUT_DIR/MY_FILE_OUT.HYB".

-l, --out_fold Optional path to one or more ".vienna" or ".ct"-format files for output (should include appropriate ".vienna"/".ct" suffix). If not provided, the output for input file "PATH_TO/MY_FILE.VIENNA" will be used as a template for the output "OUT_DIR/MY_FILE_OUT.VIENNA".

-d, --out_dir Path to directory for output of files. Defaults to the current working directory.

Default: $PWD

-u, --out_suffix Suffix to add to the name of output files, before any file- or analysis-specific suffixes. The file-type appropriate suffix will be added automatically.

Default: "_evaluated"

-t, --eval_types Possible choices: type, mirna

Types of evaluations to perform on input hyb file. (Note: evaluations can be combined, such as "--eval_types type mirna")

Default: ['type']

--set_dataset Set "dataset" flag to value of the input file name.

Default: False

--version Print version and exit.

-v, --verbose Print verbose output during run.

Default: False

-s, --silent Print no output during run.

Default: False

3.3.2 type Analysis Options

--type_method Possible choices: hybformat, string_match, id_map

Segment-type finding method to use for type evaluation. For a description of the different methods, see the HybRecord documentation for the eval_types method.

Default: "hybformat"

--type_params_file Segment-type finding parameters file to use for type evaluation with some type finding methods: {string_match, id_map}. For a description of the different methods, see the HybRecord documentation for the find_seg_types method.

3.3.3 Hyb Record Settings

--mirna_types "seg_type" fields identifying a miRNA

Default: ['miRNA', 'microRNA']

--custom_flags Custom flags to allow in addition to those specified in the hybkit specification.

Default: []

--hyb_placeholder placeholder character/string for missing data in hyb files.

Default: "."

--reorder_flags Possible choices: True, False

Re-order flags to the hybkit-specification order when writing hyb records.

Default: True

--allow_undefined_flags Possible choices: True, False

Allow use of flags not defined in the hybkit-specification order when reading and writing hyb records. As the preferred alternative to using this setting, the --custom_flags argument can be be used to supply custom allowed flags.

Default: False

--allow_unknown_seg_types Possible choices: True, False

Allow unknown segment types when assigning segment types.

Default: False

3.3.4 Hyb File Settings

--hybformat_id Possible choices: True, False

The Hyb Software Package places further information in the "id" field of the hybrid record that can be used to infer the number of contained read counts. When set to True, the identifiers will be parsed as: "<read_id>_<read_count>"

Default: False

--hybformat_ref Possible choices: True, False

The Hyb Software Package uses a reference database with identifiers that contain sequence type and other sequence information. When set to True, all hyb file identifiers will be parsed as: "<gene_id>_<transcript_id>_<gene_name>_<seg_type>"

Default: False

3.3.5 Fold Record Settings

--allowed_mismatches For DynamicFoldRecords, allowed number of mismatches with a HybRecord.

Default: 0

--fold_placeholder Placeholder character/string for missing data for reading/writing fold records.

Default: "."

-y, --seq_type Possible choices: static, dynamic

Type of fold record object to use. Options: "static": FoldRecord, requires an exact sequence match to be paired with a HybRecord; "dynamic": DynamicFoldRecord, requires a sequence match to the "dynamic" annotated regions of a HybRecord, and may be shorter/longer than the original sequence.

Default: "static"

--error_mode Possible choices: raise, warn_return, return

Mode for handling errors during reading of HybFiles (overridden by HybFoldIter.settings['iter_error_mode'] when using HybFoldIter). Options: "raise": Raise an error when encountered and exit program ; "warn_return": Print a warning and return the error_value ; "return": Return the error value with no program output. record is encountered.

Default: "raise"

3.3.6 Hyb-Fold Iterator Settings

--error_checks Possible choices: hybrecord_indel, foldrecord_nofold, max_mismatch, energy_mismatch

Error checks for simultaneous HybFile and FoldFile parsing. Options: "hybrecord_indel": Error for HybRecord objects where one/both sequences have insertions/deletions in alignment, which prevents matching of sequences; "foldrecord_nofold": Error when failure in reading a fold_record object; "max_mismatch": Error when mismatch between hybrecord and foldrecord sequences is greater than FoldRecord "allowed_mismatches" setting; "energy_mismatch": Error when a mismatch exists between HybRecord and FoldRecord energy values.

Default: ['hybrecord_indel', 'foldrecord_nofold', 'max_mismatch', 'energy_mismatch']

--iter_error_mode Possible choices: raise, warn_return, warn_skip, skip, return

Mode for handling errors found during error checks. Overrides HybRecord "error_mode" setting when using HybFoldIter. Options: "raise": Raise an error when encountered; "warn_return": Print a warning and return the value; "warn_skip": Print a warning and continue to the next iteration; "skip": Continue to the next iteration without any output; "return": return the value without any error output;

Default: "warn_skip"

--max_sequential_skips Maximum number of record(-pairs) to skip in a row. Limited as several sequential skips usually indicates an issue with record formatting or a desynchronization between files.

Default: 100

Output File Naming:

Output files can be named in two fashions: via automatic name generation, or by providing specific out file names.

Automatic Name Generation:

For output name generation, the default respective naming scheme is used:

```
hyb_script -i PATH_TO/MY_FILE_1.HYB [...]
    -->  OUT_DIR/MY_FILE_1_ADDSUFFIX.HYB
```

This output file path can be modified with the arguments {--out_dir, --out_suffix} described below.

The output directory defaults to the current working directory (`$PWD`), and can be modified with the `--out_dir <dir>` argument. Note: The provided directory must exist, or an error will be raised. For Example:

```
hyb_script -i PATH_TO/MY_FILE_1.HYB [...] --out_dir MY_OUT_DIR
    -->  MY_OUT_DIR/MY_FILE_1_ADDSUFFIX.HYB
```

The suffix used for output files is based on the primary actions of the script. It can be specified using `--out_suffix <suffix>`. This can optionally include the ".hyb" final suffix. for Example:

```
hyb_script -i PATH_TO/MY_FILE_1.HYB [...] --out_suffix MY_SUFFIX
    -->  OUT_DIR/MY_FILE_1_MY_SUFFIX.HYB
#OR
hyb_script -i PATH_TO/MY_FILE_1.HYB [...] --out_suffix MY_SUFFIX.HYB
    -->  OUT_DIR/MY_FILE_1_MY_SUFFIX.HYB
```

Specific Output Names:

Alternatively, specific file names can be provided via the -o/--out_hyb argument, ensuring that the same number of input and output files are provided. This argument takes precedence over all automatic output file naming options (--out_dir, --out_suffix), which are ignored if -o/--out_hyb is provided. For Example:

```
hyb_script [...] --out_hyb MY_OUT_DIR/OUT_FILE_1.HYB MY_OUT_DIR/OUT_FILE_2.HYB
    -->  MY_OUT_DIR/OUT_FILE_1.hyb
    -->  MY_OUT_DIR/OUT_FILE_2.hyb
```

Note: The directory provided with output file paths (MY_OUT_DIR above) must exist, otherwise an error will be raised.

3.4 hyb_analyze

Read hyb / vienna files and analyze the fold information in the contained hybrid sequences.

Analysis Types:

Energy (page 40)	Analysis of values of predicted intra-hybrid folding energy
Type (page 40)	Analysis of segment types
miRNA (page 41)	Analysis of miRNA segments distributions
Target (page 41)	Analysis of mirna target segment names and types
Fold (page 42)	Analysis of folding data included in the analyzed hyb_records

This utility reads in one or more files in hyb-format (see the *hybkit Hyb File Specification* (page 5)) along with a corresponding predicted RNA secondary structure file in the "Vienna" (*Vienna Format* (page 23)) or "CT" (*CT_Format* (page 23)) formats, and analyzes hybrid secondary structure properties.

For full information on the different analysis types, see the *Analyses* (page 40) section of the hybkit documentation.

Example system calls:

```
$ hyb_analyze -a fold -i my_file_1.hyb -f my_file_1.vienna

$ hyb_analyze -a fold -i my_file_2.hyb -f my_file_2.ct \\
            --make_plots False
```

```
usage: hyb_analysis [-h] -i PATH_TO/MY_FILE.HYB [PATH_TO/MY_FILE.HYB ...]
                    [-f [PATH_TO/MY_FILE.VIENNA [PATH_TO/MY_FILE.VIENNA ...]]]
                    [-o PATH_TO/OUT_BASENAME [PATH_TO/OUT_BASENAME ...]]
                    [-d OUT_DIR] [-u OUT_SUFFIX]
                    [-a {energy,type,mirna,target,fold} [{energy,type,mirna,target,fold}␣
↪...]]
                    [-n ANALYSIS_NAME] [-p {True,False}] [--version] [-v | -s]
                    [--mirna_types MIRNA_TYPES [MIRNA_TYPES ...]]
                    [--custom_flags CUSTOM_FLAGS [CUSTOM_FLAGS ...]]
                    [--hyb_placeholder HYB_PLACEHOLDER]
                    [--reorder_flags {True,False}]
                    [--allow_undefined_flags [{True,False}]]
                    [--allow_unknown_seg_types [{True,False}]]
                    [--hybformat_id [{True,False}]]
                    [--hybformat_ref [{True,False}]]
                    [--allowed_mismatches ALLOWED_MISMATCHES]
                    [--fold_placeholder FOLD_PLACEHOLDER]
                    [-y {static,dynamic}]
                    [--error_mode {raise,warn_return,return}]
                    [--error_checks {hybrecord_indel,foldrecord_nofold,max_mismatch,
↪energy_mismatch}]
                    [--iter_error_mode {raise,warn_return,warn_skip,skip,return}]
                    [--max_sequential_skips MAX_SEQUENTIAL_SKIPS]
                    [--quant_mode {single,reads,records}]
                    [--out_delim OUT_DELIM]
```

3.4.1 Named Arguments

-i, --in_hyb REQUIRED path to one or more hyb-format files with a ".hyb" suffix for use in the evaluation.

-f, --in_fold REQUIRED path to one or more RNA secondary-structure files with a ".vienna" or ".ct" suffix for use in the evaluation.

-o, --out_basename Optional path to one or more basename prefixes to use for output. The appropriate suffix will be added based on the specific name. If not provided, the output for input file "PATH_TO/MY_FILE.HYB" will be used as a template for the basename "OUT_DIR/MY_FILE".

-d, --out_dir Path to directory for output of files. Defaults to the current working directory.

Default: $PWD

-u, --out_suffix Suffix to add to the name of output files, before any file- or analysis-specific suffixes. The file-type appropriate suffix will be added automatically.

-a, --analysis_types Possible choices: energy, type, mirna, target, fold

Analysis to perform on input hyb and fold files.

Default: "fold"

-n, --analysis_name Name / title of analysis data.

-p, --make_plots Possible choices: True, False

Create plots of analysis output.

Default: True

--version Print version and exit.

-v, --verbose Print verbose output during run.

Default: False

-s, --silent Print no output during run.

Default: False

3.4.2 Hyb Record Settings

--mirna_types "seg_type" fields identifying a miRNA

Default: ['miRNA', 'microRNA']

--custom_flags Custom flags to allow in addition to those specified in the hybkit specification.

Default: []

--hyb_placeholder placeholder character/string for missing data in hyb files.

Default: "."

--reorder_flags Possible choices: True, False

Re-order flags to the hybkit-specification order when writing hyb records.

Default: True

--allow_undefined_flags Possible choices: True, False

Allow use of flags not defined in the hybkit-specification order when reading and writing hyb records. As the preferred alternative to using this setting, the --custom_flags argument can be be used to supply custom allowed flags.

Default: False

--allow_unknown_seg_types Possible choices: True, False

Allow unknown segment types when assigning segment types.

Default: False

3.4.3 Hyb File Settings

--hybformat_id Possible choices: True, False

The Hyb Software Package places further information in the "id" field of the hybrid record that can be used to infer the number of contained read counts. When set to True, the identifiers will be parsed as: "<read_id>_<read_count>"

Default: False

--hybformat_ref Possible choices: True, False

The Hyb Software Package uses a reference database with identifiers that contain sequence type and other sequence information. When set to True, all hyb file identifiers will be parsed as: "<gene_id>_<transcript_id>_<gene_name>_<seg_type>"

Default: False

3.4.4 Fold Record Settings

--allowed_mismatches For DynamicFoldRecords, allowed number of mismatches with a HybRecord.

Default: 0

--fold_placeholder Placeholder character/string for missing data for reading/writing fold records.

Default: "."

-y, --seq_type Possible choices: static, dynamic

Type of fold record object to use. Options: "static": FoldRecord, requires an exact sequence match to be paired with a HybRecord; "dynamic": DynamicFoldRecord, requires a sequence match to the "dynamic" annotated regions of a HybRecord, and may be shorter/longer than the original sequence.

Default: "static"

--error_mode Possible choices: raise, warn_return, return

Mode for handling errors during reading of HybFiles (overridden by HybFoldIter.settings['iter_error_mode'] when using HybFoldIter). Options: "raise": Raise an error when encountered and exit program ; "warn_return": Print a warning and return the error_value ; "return": Return the error value with no program output. record is encountered.

Default: "raise"

3.4.5 Hyb-Fold Iterator Settings

--error_checks Possible choices: hybrecord_indel, foldrecord_nofold, max_mismatch, energy_mismatch

Error checks for simultaneous HybFile and FoldFile parsing. Options: "hybrecord_indel": Error for HybRecord objects where one/both sequences have insertions/deletions in alignment, which prevents matching of sequences; "foldrecord_nofold": Error when failure in reading a fold_record object; "max_mismatch": Error when mismatch between hybrecord and foldrecord sequences is greater than FoldRecord "allowed_mismatches" setting; "energy_mismatch": Error when a mismatch exists between HybRecord and FoldRecord energy values.

Default: ['hybrecord_indel', 'foldrecord_nofold', 'max_mismatch', 'energy_mismatch']

--iter_error_mode Possible choices: raise, warn_return, warn_skip, skip, return

Mode for handling errors found during error checks. Overrides HybRecord "error_mode" setting when using HybFoldIter. Options: "raise": Raise an error when encountered; "warn_return": Print a warning and return the value; "warn_skip": Print a warning and continue to the next iteration; "skip": Continue to the next iteration without any output; "return": return the value without any error output;

Default: "warn_skip"

--max_sequential_skips Maximum number of record(-pairs) to skip in a row. Limited as several sequential skips usually indicates an issue with record formatting or a desynchronization between files.

Default: 100

3.4.6 Analysis Settings

--quant_mode Possible choices: single, reads, records

Method for counting records. Options: "single": Count each record as a single entry; "reads": Use the number of reads per hyb record as the count (may contain PCR duplicates); "records": Count the number of records represented by each hyb record entry (1 for "unmerged" records, >= 1 for "merged" records)

Default: "single"

--out_delim Delimiter-string to place between fields in analysis output.

Default: ","

Output File Naming:

Output files can be named in two fashions: via automatic name generation, or by providing specific out file names.

Automatic Name Generation:

For output name generation, the default respective naming scheme is used:

```
hyb_script -i PATH_TO/MY_FILE_1.HYB [...]
    -->  OUT_DIR/MY_FILE_1_ADDSUFFIX.HYB
```

This output file path can be modified with the arguments {--out_dir, --out_suffix} described below.

The output directory defaults to the current working directory (`$PWD`), and can be modified with the `--out_dir <dir>` argument. Note: The provided directory must exist, or an error will be raised. For Example:

```
hyb_script -i PATH_TO/MY_FILE_1.HYB [...] --out_dir MY_OUT_DIR
    -->  MY_OUT_DIR/MY_FILE_1_ADDSUFFIX.HYB
```

The suffix used for output files is based on the primary actions of the script. It can be specified using `--out_suffix <suffix>`. This can optionally include the ".hyb" final suffix. for Example:

```
hyb_script -i PATH_TO/MY_FILE_1.HYB [...] --out_suffix MY_SUFFIX
    -->  OUT_DIR/MY_FILE_1_MY_SUFFIX.HYB
#OR
hyb_script -i PATH_TO/MY_FILE_1.HYB [...] --out_suffix MY_SUFFIX.HYB
    -->  OUT_DIR/MY_FILE_1_MY_SUFFIX.HYB
```

Specific Output Names:

Alternatively, specific file names can be provided via the -o/--out_hyb argument, ensuring that the same number of input and output files are provided. This argument takes precedence over all automatic output file naming options (--out_dir, --out_suffix), which are ignored if -o/--out_hyb is provided. For Example:

```
hyb_script [...] --out_hyb MY_OUT_DIR/OUT_FILE_1.HYB MY_OUT_DIR/OUT_FILE_2.HYB
    -->  MY_OUT_DIR/OUT_FILE_1.hyb
    -->  MY_OUT_DIR/OUT_FILE_2.hyb
```

Note: The directory provided with output file paths (MY_OUT_DIR above) must exist, otherwise an error will be raised.

CHAPTER FOUR

EXAMPLE ANALYSES

This section includes multiple example stepwise analyses of data from a qCLASH experiment described in [Gay2018], with data acquired from the NCBI Gene Expression Omnibus (GEO) accession GSE101978[807].

Each analysis is implemented both using the Python3 API, and as a sequence of shell executable commands in a bash script. The Python API implementations are generally significantly more efficient as more steps can be performed on a single iteration over the input data.

Each analysis performs quality control steps on the data by checking data integrity (*hyb_check* (page 61)) and removing artifactual ribosomal- and mitochondrial-RNA hybrids (*hyb_filter* (page 65)). Further filtration may be performed, and then each described analysis is carried out.

Pipeline	Description
Example Type-miRNA Analysis (page 82)	Quantify the sequence and miRNA types in a hyb file
Example Target Analysis (page 85)	Analyze targets of a set of miRNAs from a single experiment
Example Grouped Target Analysis (page 86)	Analyze and plot targets of a set of miRNAs from pooled experimental replicates
Example Fold Analysis (page 87)	Analyze and plot predicted miRNA folding patterns in miRNA-containing hybrids

Further details on each respective example analysis can be found in each section.

4.1 Example Type-miRNA Analysis

This directory contains a example analysis of Hyb-format data, published in the quick Crosslinking and Sequencing of Hybrids (qCLASH) experiment described in: Gay, Lauren A., et al. "Modified cross-linking, ligation, and sequencing of hybrids (qCLASH) identifies Kaposi's Sarcoma-associated herpesvirus microRNA targets in endothelial cells." Journal of virology 92.8 (2018): e02138-17.

The analysis is carried out in multiple example implementations which produce identical output:

- via the Command-Line[808]
- via the Python3 API[809]

[807] https://www.ncbi.nlm.nih.gov/geo/query/acc.cgi?acc=GSE101978
[808] https://github.com/RenneLab/hybkit/blob/master/example_01_type_mirna_analysis/analysis_shell.sh/
[809] https://github.com/RenneLab/hybkit/blob/master/example_01_type_mirna_analysis/analysis_python.py/

This analysis first performs quality control on the data. It then summarizes and analyzes the hybrid, segment, and miRNA characteristics of each input file. Analyses for each individual file, and a combined summary analysis are all produced.

The sequencing information is available at NCBI Gene Expression Omnibus (GEO) GSE101978, at:

https://www.ncbi.nlm.nih.gov/geo/query/acc.cgi?acc=GSE101978

The data files can be downloaded and uncompressed by using the command:

```
$ sh ./download_data.sh
```

The unpacked hyb data-files require ~2 GB of space. The completed output of the analysis requires ~1.5 GB of space.

4.1.1 Type-miRNA Analysis Example Output

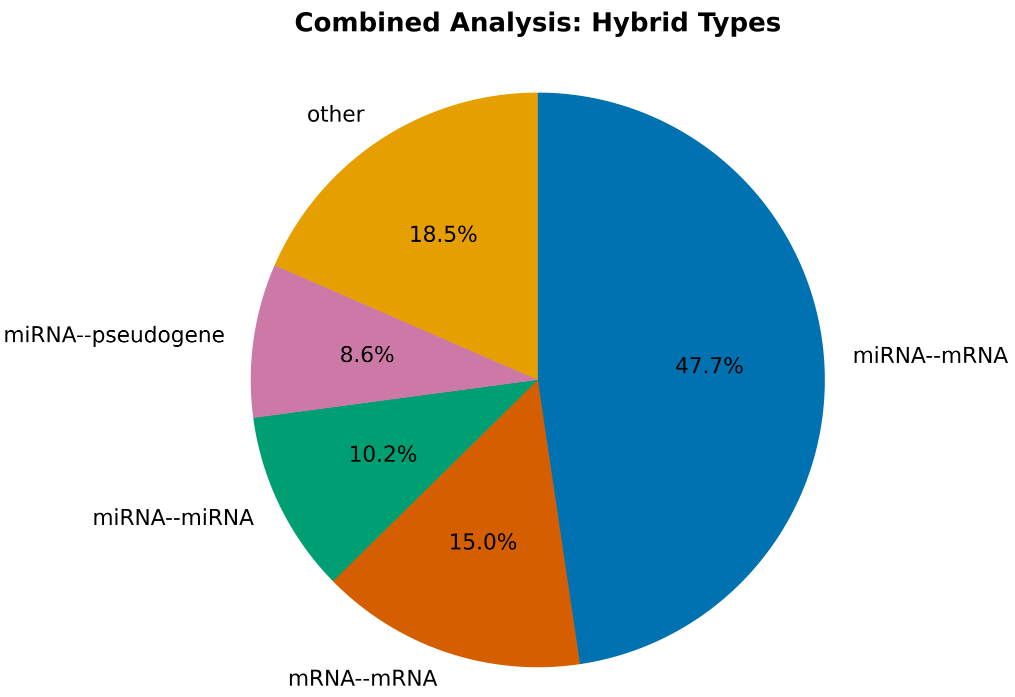

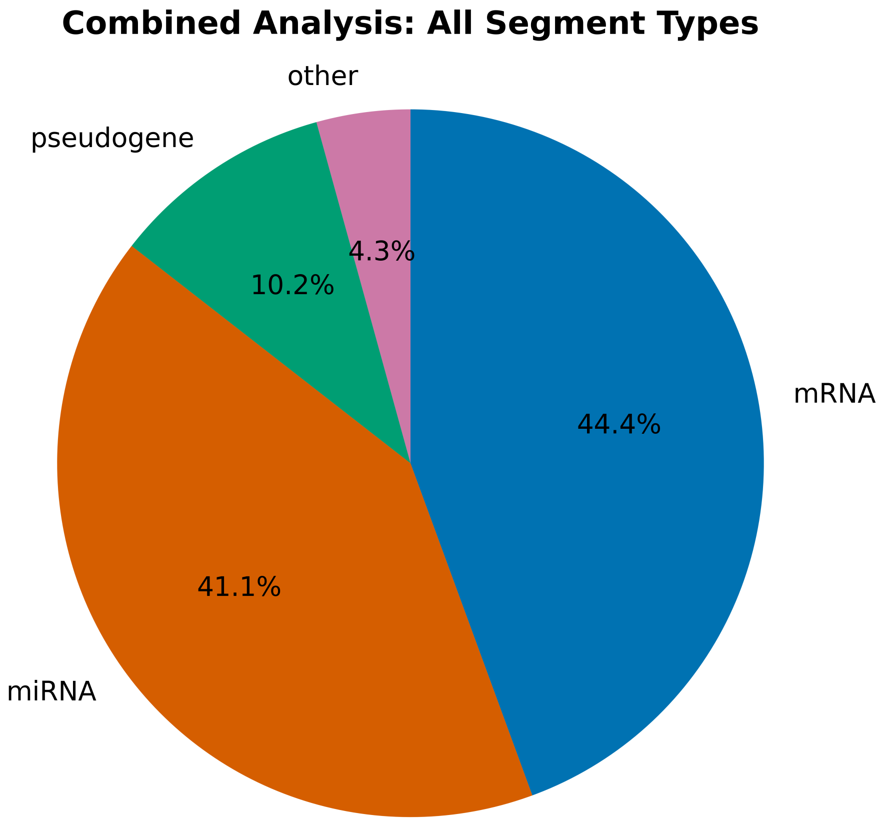
Combined Analysis: All Segment Types
other
pseudogene
4.3%
10.2%
mRNA
44.4%
41.1%
miRNA

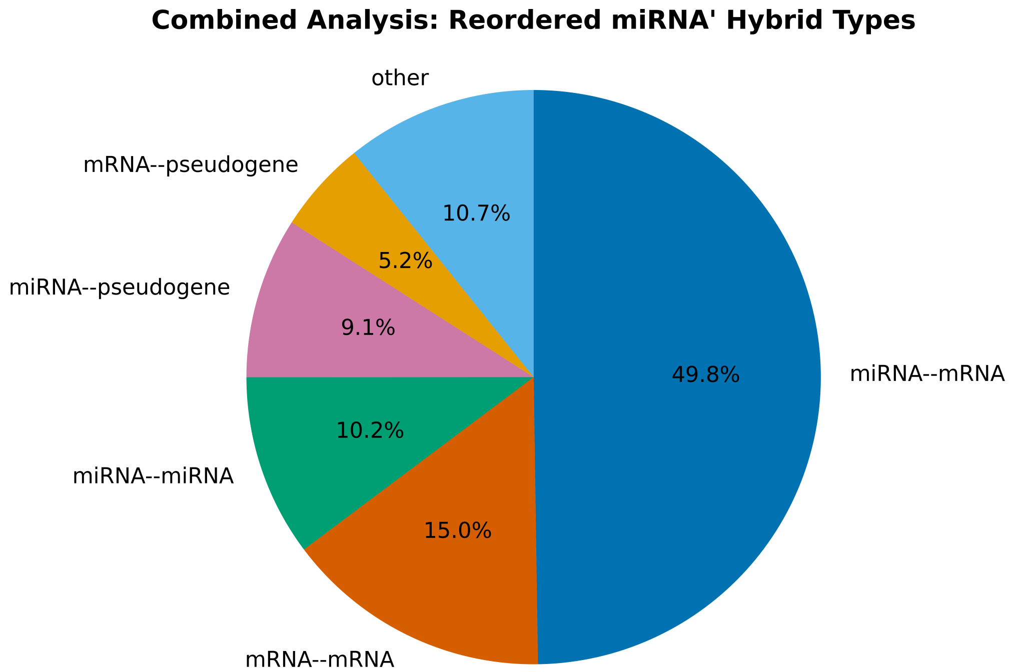
Combined Analysis: Reordered miRNA' Hybrid Types
other
mRNA--pseudogene
10.7%
5.2%
miRNA--pseudogene
9.1%
49.8%
miRNA--mRNA
10.2%
miRNA--miRNA
15.0%
mRNA--mRNA

4.2 Example Target Analysis

This directory contains a example analysis of Hyb-format data, published in the quick Crosslinking and Sequencing of Hybrids (qCLASH) experiment described in: Gay, Lauren A., et al. "Modified cross-linking, ligation, and sequencing of hybrids (qCLASH) identifies Kaposi's Sarcoma-associated herpesvirus microRNA targets in endothelial cells." Journal of virology 92.8 (2018): e02138-17.

The analysis is carried out in multiple example implementations which produce identical output:

- via the Command-Line[810]
- via the Python3 API[811]

This analysis specifically filters and analyzes the kshv-miR-K12-5 miRNA arising from Kaposi's Sarcoma-Associated Herpesvirus (KSHV), which has the assigned type "KSHV-miRNA". Both individual and summary output files are produced.

Hybrid sequences generated by the Hyb program are available at NCBI Gene Expression Omnibus (GEO) GSE101978, at:

https://www.ncbi.nlm.nih.gov/geo/query/acc.cgi?acc=GSE101978

The data files can be downloaded and uncompressed by using the command:

```
$ sh ./download_data.sh
```

The unpacked hyb data-file require ~130 MB of space. The completed output of the analysis requires ~20 MB of space.

4.2.1 Target Analysis Example Output

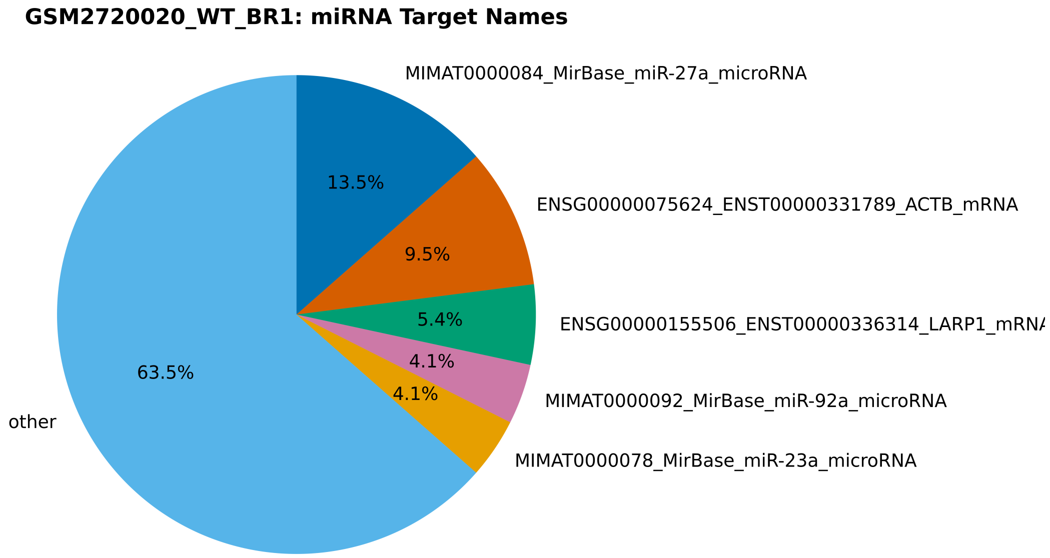

810 https://github.com/RenneLab/hybkit/blob/master/example_02_target_analysis/analysis_shell.sh/
811 https://github.com/RenneLab/hybkit/blob/master/example_02_target_analysis/analysis_python.py/

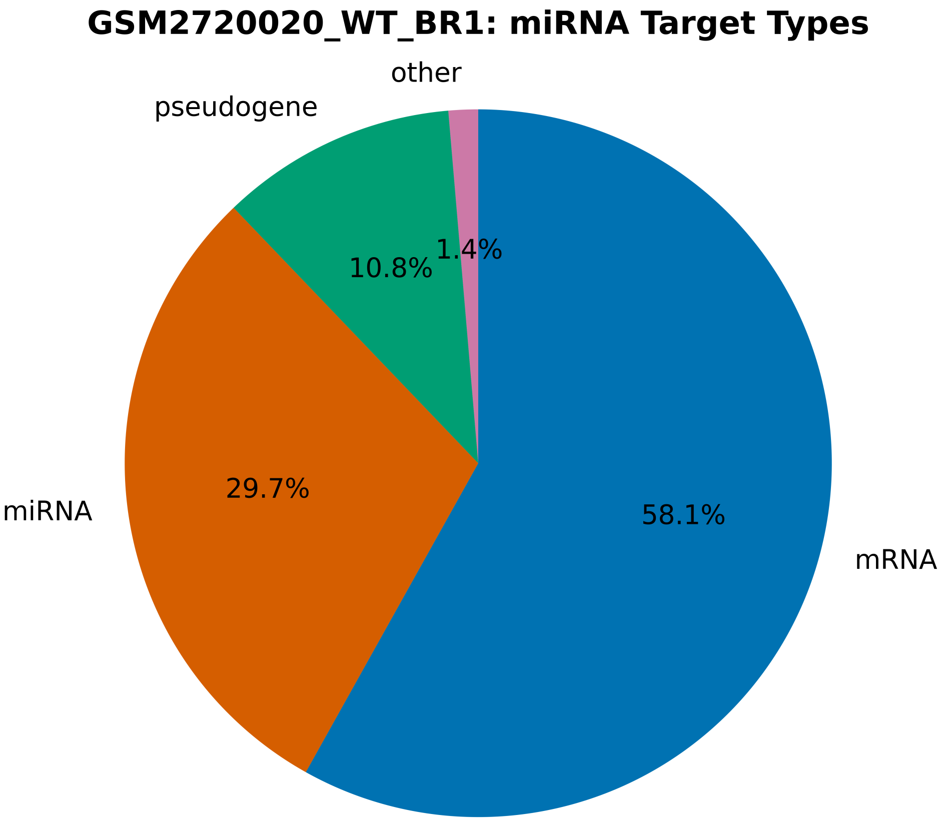

4.3 Example Grouped Target Analysis

This directory contains a example analysis of Hyb-format data, published in the quick Crosslinking and Sequencing of Hybrids (qCLASH) experiment described in: Gay, Lauren A., et al. "Modified cross-linking, ligation, and sequencing of hybrids (qCLASH) identifies Kaposi's Sarcoma-associated herpesvirus microRNA targets in endothelial cells." Journal of virology 92.8 (2018): e02138-17.

The analysis is carried out in multiple example implementations which produce identical output:

- via the Command-Line[812]
- via the Python3 API[813]

This analysis specifically investigates and characterizes miRNA arising from six experimental replicates from two conditions with cells infected with Kaposi's Sarcoma Herpesvirus, which are given the type name "KSHV_miRNA". The hybrid reads from KSHV miRNA are grouped and analyzed toghether. Both individual and summary output files are produced.

Hybrid sequence information created by the Hyb program information is available at NCBI Gene Expression Ombnibus (GEO) GSE101978, at:

https://www.ncbi.nlm.nih.gov/geo/query/acc.cgi?acc=GSE101978

[812] https://github.com/RenneLab/hybkit/blob/master/example_03_grouped_target_analysis/analysis_shell.sh/
[813] https://github.com/RenneLab/hybkit/blob/master/example_03_grouped_target_analysis/analysis_python.py/

The data files can be downloaded and uncompressed by using the command:

```
$ sh ./download_data.sh"
```

The unpacked hyb data-file require ~1.3 GB of space. The completed output of the analysis requires ~40 MB of space.

4.3.1 Grouped Target Analysis Example Output

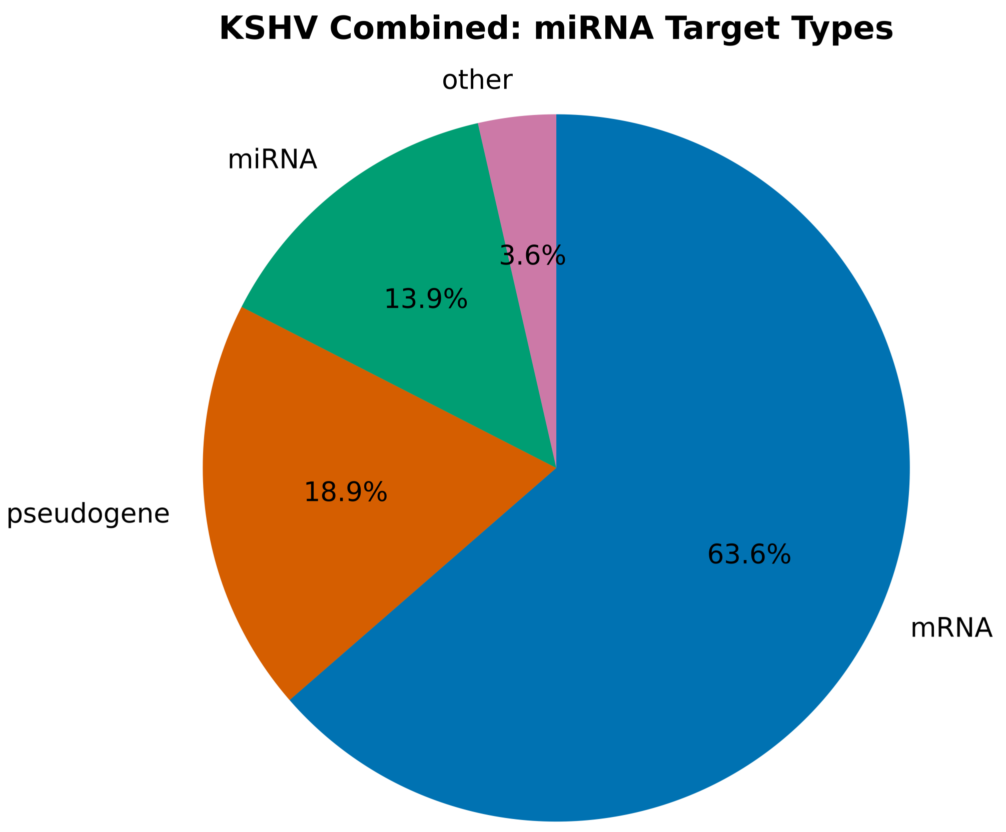

4.4 Example Fold Analysis

This directory contains a example analysis of Hyb-format and Vienna-format data, published in the quick Crosslinking and Sequencing of Hybrids (qCLASH) experiment described in: Gay, Lauren A., et al. "Modified cross-linking, ligation, and sequencing of hybrids (qCLASH) identifies Kaposi's Sarcoma-associated herpesvirus microRNA targets in endothelial cells." Journal of virology 92.8 (2018): e02138-17.

The analysis is carried out in multiple example implementations which produce identical output:

- via the Command-Line[814]
- via the Python3 API[815]

[814] https://github.com/RenneLab/hybkit/blob/master/example_04_fold_analysis/analysis_shell.sh/
[815] https://github.com/RenneLab/hybkit/blob/master/example_04_fold_analysis/analysis_python.py/

This analysis investigates the predicted folding of miRNA from an experimental replicate infected with Kaposi's Sarcoma-Associated Herpesvirus (KSHV), which are given the type name "KSHV-miRNA". Data from the predicted folding fold for each hybrid record produced by the "Hyb" program are analyzed, and the folds of each KSHV miRNA with a non-miRNA target are characterized to determine the per-base folding folds.

Hybrid sequence information created by the Hyb program and the fold output are provided with the hybkit package in the databases directory, created by downstream analysis of files available at NCBI Gene Expression Omnibus (GEO) GSE101978, at:

https://www.ncbi.nlm.nih.gov/geo/query/acc.cgi?acc=GSE101978

The data files can be copied and uncompressed by using the command:

```
$ sh ./prepare_data.sh
```

The unpacked data-files require ~150 MB of space. The completed output of the analysis requires ~30 MB of space.

4.4.1 Fold Analysis Example Output

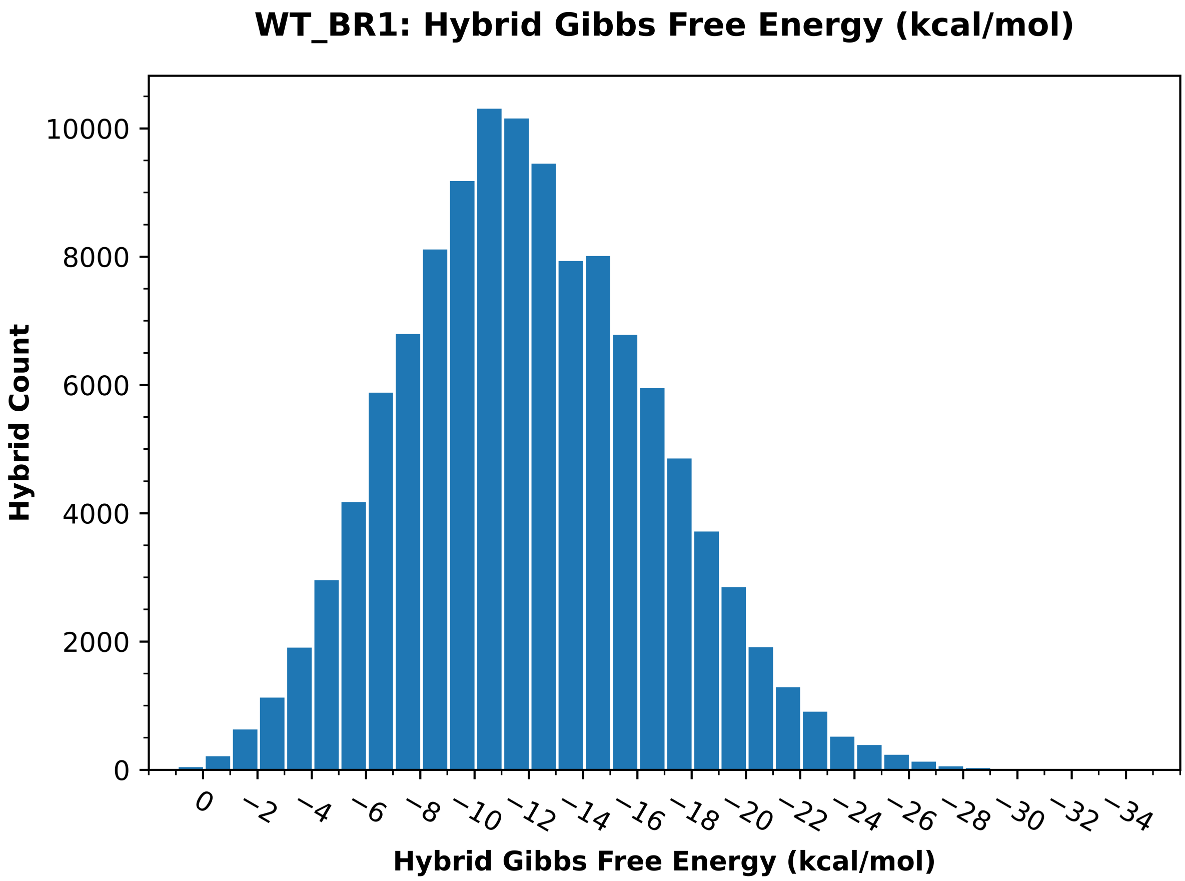

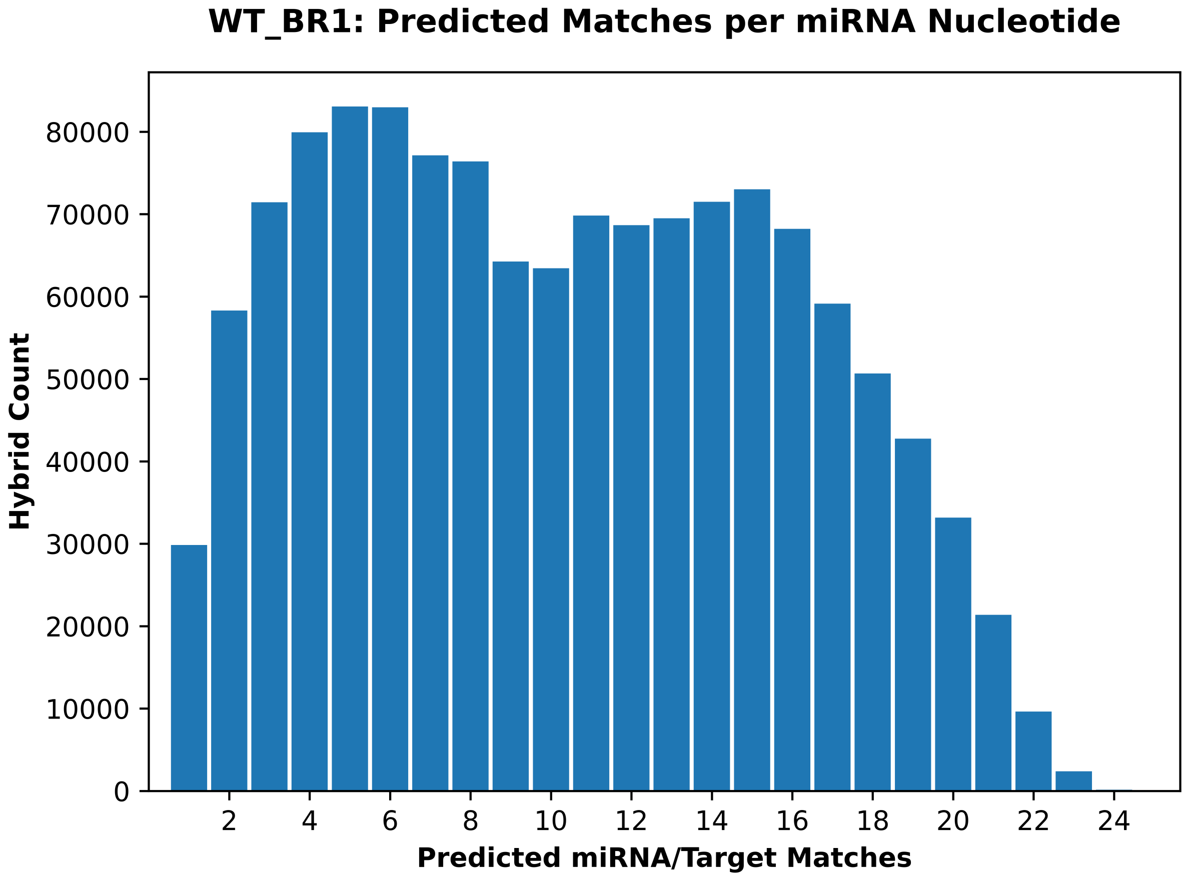

WT_BR1: Predicted Matches per miRNA Nucleotide
Hybrid Count
80000
70000
60000
50000
40000
30000
20000
10000
0
2
4
6
8
10
12
14
16
18
20
22
24
Predicted miRNA/Target Matches

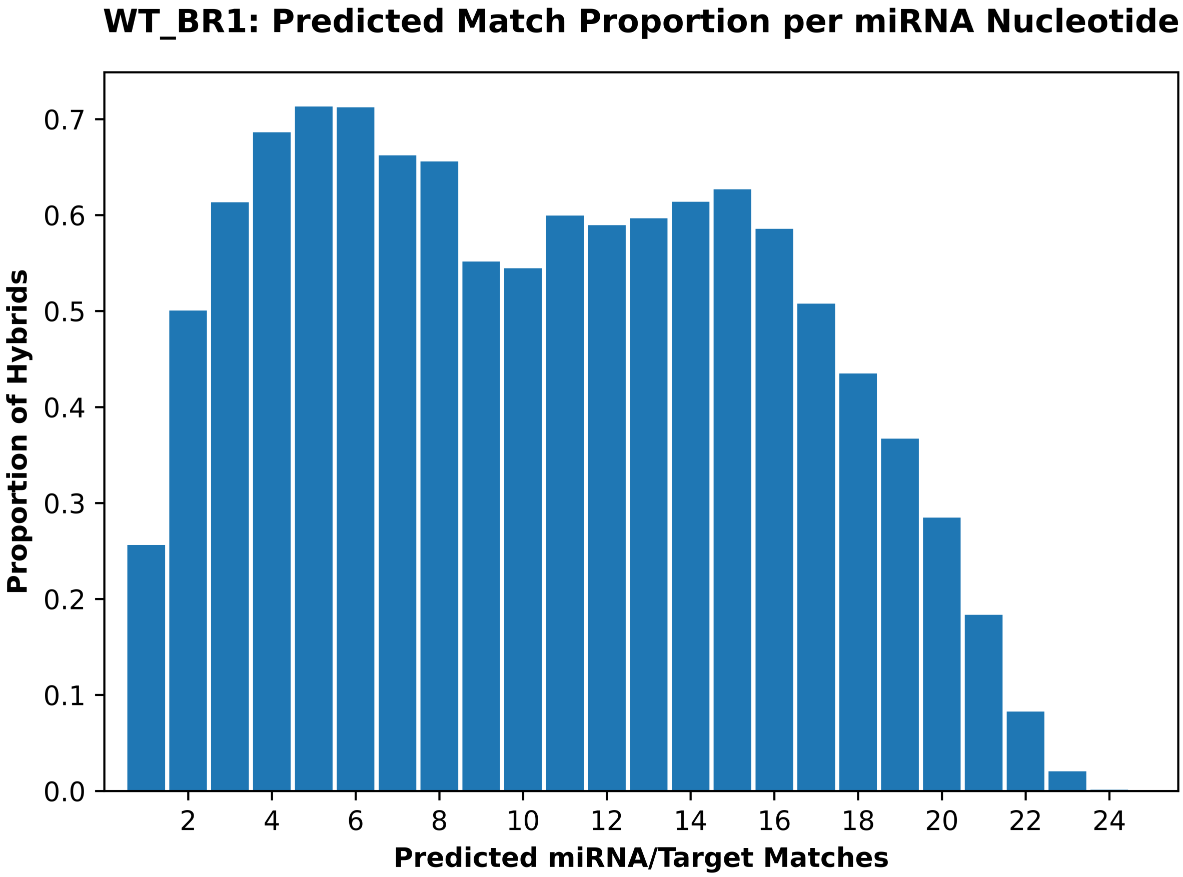
WT_BR1: Predicted Match Proportion per miRNA Nucleotide
Proportion of Hybrids
0.0
0.1
0.2
0.3
0.4
0.5
0.6
0.7
2
4
6
8
10
12
14
16
18
20
22
24
Predicted miRNA/Target Matches

CHAPTER

FIVE

ABOUT

5.1 Renne Lab

Principal Investigator: Rolf Renne
Henry E. Innes Professor of Cancer Research
University of Florida
UF Health Cancer Center
UF Department of Molecular Genetics and Microbiology
UF Genetics Institute
http://www.rennelab.com

5.2 Lead Developer

- Daniel Stribling <ds@ufl.edu>
  https://www.danthescienceman.com
  https://orcid.org/0000-0002-0649-9506
  University of Florida, Renne Lab

5.3 Changelog

- 0.3.4 (2023-11) Changes include:
  - Misc Bugfixes and Refinements
  - Switch code linting to Ruff
  - Add hybkit.errors module and HybkitError classes
  - Moved printing of warnings to python logging module
  - Add option for direct passage of file-like object for construction of HybFile and ViennaFile
  - Add HybRecord.to_csv_header(), HybRecord.to_fields(), and HybRecord.to_fields_header() methods
  - Refine description of HybFile.open() constructor method
  - Add typing_extensions dependency
  - Add Python3.8-compatible type hints to API
  - Documentation Updates

- 0.3.3 (2023-09) Changes include:
  - Misc Bugfixes and Refinements
  - Update integer bar-plot functions
- 0.3.2 (2023-08) Changes include:
  - Misc Bugfixes and Refinements
  - Add duplicate hybrid filtration (by HybRecord.id) options to hyb_filter
  - Add duplicate hybrid filtration to example analyses
- 0.3.1 (2023-08) Changes include:
  - Misc Bugfixes and Refinements
  - Add --version flag to scripts
  - Change move scripts output file description to argparse epilog
  - Refine plot functions
  - Change default plot colors to the Bang Wong scheme [Wong2011] for colorblind accessibility
  - Documentation corrections
  - Spellcheck
- 0.3.0 (2023-04) Major Codebase And API Overhaul. Changes include:
  - Simplifying HybRecord API
  - Simplifying FoldRecord API
  - Unifying settings information for argparse and modules
  - Removing Support for ViennaD format
  - Moving identifier-parsing code to module type_finder
  - Moving target region analysis code to module region_finder
  - Moving code for settings into a "settings" module
  - Renamed HybRecord type_analysis and mirna_analysis to eval_types and eval_mirna, respectively to differentiate from analysis module functions
  - Reimplemented analyses methods within a single Analysis class
  - Added error checking / catching to HybFoldIter
  - Removed Target-Region Analysis and associated files due to lack of archival database information, pending future development
  - Added "dynamic" seq_type to FoldRecord for non-identical fold/hybrid sequence handling
  - Added shell implementation to all example analyses
  - Remove support for Python3.6, Python3.7
  - Migrate to CircleCI for CI/CD
  - Added Pytest unit testing integrated with CircleCI
  - Other Misc. Improvements / Bugfixes
- 0.2.0 (2020-03) Added Command-line Toolkit. Code Refinements.
- 0.1.9 (2020-03) Fix for Module Path Finding for Python > 3.6

- 0.1.8 (2020-03) Streamlining, PyPI / PIP Initial Release
- 0.1.0 (2020-01) Initial Implementation

CHAPTER

SIX

REFERENCES

- [ViennaFormat]
- [CTFormat]
- [Zuker2003]
- [Hunter2007]
- [Cock2009]
- [Lorenz2011]
- [Wong2011]
- [Helwak2013]
- [Travis2014]
- [Gay2018]

BIBLIOGRAPHY

[ViennaFormat] ViennaRNA Vienna File Format Description[816]
UNAFold Vienna File Format Description[817]

[CTFormat] UNAFold CT Format Description[818]
RNAStructure CT Format Description[819]

[Zuker2003] Zuker M. Mfold web server for nucleic acid folding and hybridization prediction. Nucleic Acids Res. 2003 Jul 1;31(13):3406-15. doi: 10.1093/nar/gkg595[820]. PMID: 12824337; PMCID: PMC169194.

[Hunter2007] J. Hunter, "Matplotlib: A 2D Graphics Environment" in Computing in Science & Engineering, vol. 9, no. 03, pp. 90-95, 2007. doi: 10.1109/MCSE.2007.55[821]

[Cock2009] Cock PJ, Antao T, Chang JT, Chapman BA, Cox CJ, Dalke A, Friedberg I, Hamelryck T, Kauff F, Wilczynski B, de Hoon MJ. Biopython: freely available Python tools for computational molecular biology and bioinformatics. Bioinformatics. 2009 Jun 1;25(11):1422-3. doi: 10.1093/bioinformatics/btp163[822]. Epub 2009 Mar 20. PMID: 19304878; PMCID: PMC2682512.

[Lorenz2011] Lorenz, R., Bernhart, S.H., Höner zu Siederdissen, C. et al. ViennaRNA Package 2.0. Algorithms Mol Biol 6, 26 (2011). doi: 10.1186/1748-7188-6-26[823]

[Wong2011] Wong, B. Points of view: Color blindness. Nat Methods 8, 441 (2011). doi: 10.1038/nmeth.1618[824]

[Helwak2013] Helwak A, Kudla G, Dudnakova T, Tollervey D. Mapping the human miRNA interactome by CLASH reveals frequent noncanonical binding. Cell. 2013 Apr 25;153(3):654-65. doi: 10.1016/j.cell.2013.03.043[825]. PMID: 23622248; PMCID: PMC3650559.

[Travis2014] Travis AJ, et al. Hyb: a bioinformatics pipeline for the analysis of CLASH (crosslinking, ligation and sequencing of hybrids) data. Methods. 2014 Feb;65(3):263-73. doi: 10.1016/j.ymeth.2013.10.015[826].

[Gay2018] Gay LA, Sethuraman S, Thomas M, Turner PC, Renne R. Modified Cross-Linking, Ligation, and Sequencing of Hybrids (qCLASH) Identifies Kaposi's Sarcoma-Associated Herpesvirus MicroRNA Targets in Endothelial Cells. J Virol. 2018 Mar 28;92(8):e02138-17. doi: 10.1128/JVI.02138-17[827]. PMID: 29386283; PMCID: PMC5874430.

816 https://www.tbi.univie.ac.at/RNA/tutorial/#sec2_7
817 http://www.unafold.org/doc/formats.php#VIENNA
818 http://www.unafold.org/doc/formats.php#CT
819 https://rna.urmc.rochester.edu/Text/File_Formats.html#CT
820 https://doi.org/10.1093/nar/gkg595
821 https://doi.org/10.1109/MCSE.2007.55
822 https://doi.org/10.1093/bioinformatics/btp163
823 https://doi.org/10.1186/1748-7188-6-26
824 https://doi.org/10.1038/nmeth.1618
825 https://doi.org/10.1016/j.cell.2013.03.043
826 https://doi.org/10.1016/j.ymeth.2013.10.015
827 https://doi.org/10.1128/JVI.02138-17

PYTHON MODULE INDEX

h

hyb_analyze, 77
hyb_check, 61
hyb_eval, 71
hyb_filter, 65
hybkit, 8
hybkit.analysis, 40
hybkit.errors, 60
hybkit.plot, 47
hybkit.settings, 51
hybkit.type_finder, 34
hybkit.util, 56

INDEX

A

add_hyb_record() (*hybkit.analysis.Analysis method*), 43
add_hyb_records() (*hybkit.analysis.Analysis method*), 43
ALL_FLAGS (*hybkit.HybRecord attribute*), 11
Analysis (*class in hybkit.analysis*), 40
analysis_options (*hybkit.analysis.Analysis attribute*), 42
Analysis_settings (*in module hybkit.settings*), 56
Analysis_settings_info (*in module hybkit.settings*), 55

B

BAR_DEFAULTS (*in module hybkit.plot*), 47
BAR_INT_DEFAULTS (*in module hybkit.plot*), 47

C

check_set_method() (*hybkit.type_finder.TypeFinder class method*), 35
close() (*hybkit.CtFile method*), 32
close() (*hybkit.HybFile method*), 21
close() (*hybkit.ViennaFile method*), 29
COLOR_DICT (*in module hybkit.plot*), 47
COLOR_LIST (*in module hybkit.plot*), 47
count_hyb_record_mismatches() (*hybkit.FoldRecord method*), 25
ct_exists() (*in module hybkit.util*), 57
CT_SUFFIXES (*in module hybkit.settings*), 51
CtFile (*class in hybkit*), 31

D

default_method (*hybkit.type_finder.TypeFinder attribute*), 34
dir_exists() (*in module hybkit.util*), 56
dynamic_count_hyb_record_mismatches() (*hybkit.FoldRecord method*), 25

E

ENERGY_DEFAULTS (*in module hybkit.plot*), 48
ENERGY_HIST_RC_PARAMS (*in module hybkit.plot*), 47
energy_histogram() (*in module hybkit.plot*), 48
ensure_matches_hyb_record() (*hybkit.FoldRecord method*), 26
eval_mirna() (*hybkit.HybRecord method*), 15
eval_types() (*hybkit.HybRecord method*), 14

F

file_exists() (*in module hybkit.util*), 56
find() (*hybkit.type_finder.TypeFinder class method*), 35
find_with_params (*hybkit.type_finder.TypeFinder attribute*), 34
fold_exists() (*in module hybkit.util*), 57
fold_match_counts_histogram() (*in module hybkit.plot*), 50
FOLD_MATCH_HIST_RC_PARAMS (*in module hybkit.plot*), 47
fold_mirna_nt_counts_histogram() (*in module hybkit.plot*), 51
FOLD_NT_COUNTS_HIST_RC_PARAMS (*in module hybkit.plot*), 47
FOLD_SUFFIXES (*in module hybkit.settings*), 52
FoldFile_settings (*in module hybkit.settings*), 55
FoldFile_settings_info (*in module hybkit.settings*), 54
FoldRecord (*class in hybkit*), 23
FoldRecord_settings (*in module hybkit.settings*), 55
FoldRecord_settings_info (*in module hybkit.settings*), 53
from_ct_lines() (*hybkit.FoldRecord class method*), 26
from_ct_string() (*hybkit.FoldRecord class method*), 27
from_fasta_records() (*hybkit.HybRecord class method*), 18
from_line() (*hybkit.HybRecord class method*), 17
from_vienna_lines() (*hybkit.FoldRecord class method*), 26
from_vienna_string() (*hybkit.FoldRecord class method*), 26

G

GEN_PROPS (*hybkit.HybRecord attribute*), 12

get_all_results() (*hybkit.analysis.Analysis method*), 43
get_analysis_delim_str() (*hybkit.analysis.Analysis method*), 43
get_analysis_results() (*hybkit.analysis.Analysis method*), 43
get_argparse_doc() (*in module hybkit.util*), 56
get_mirna_props() (*hybkit.HybRecord method*), 14
get_read_count() (*hybkit.HybRecord method*), 14
get_record_count() (*hybkit.HybRecord method*), 14
get_seg1_type() (*hybkit.HybRecord method*), 13
get_seg2_type() (*hybkit.HybRecord method*), 13
get_seg_types() (*hybkit.HybRecord method*), 13
get_specific_result() (*hybkit.analysis.Analysis method*), 43
get_target_props() (*hybkit.HybRecord method*), 14

H

has_prop() (*hybkit.HybRecord method*), 16
HAS_PROPS (*hybkit.HybRecord attribute*), 13
hyb_analyze
    module, 77
hyb_check
    module, 61
hyb_eval
    module, 71
hyb_exists() (*in module hybkit.util*), 56
hyb_filter
    module, 65
HYB_SUFFIXES (*in module hybkit.settings*), 51
HybFile (*class in hybkit*), 21
HybFile_settings (*in module hybkit.settings*), 55
HybFile_settings_info (*in module hybkit.settings*), 52
HybFoldIter (*class in hybkit*), 33
HybFoldIter_settings (*in module hybkit.settings*), 56
HybFoldIter_settings_info (*in module hybkit.settings*), 54
hybkit
    module, 8
hybkit.analysis
    module, 40
hybkit.errors
    module, 60
hybkit.plot
    module, 47
hybkit.settings
    module, 51
hybkit.type_finder
    module, 34
hybkit.util
    module, 56
HybkitArgError, 60
HybkitConstructorError, 60
HybkitError, 60
HybkitIterError, 60
HybkitMiscError, 60
HybRecord (*class in hybkit*), 9
HybRecord_settings (*in module hybkit.settings*), 55
HybRecord_settings_info (*in module hybkit.settings*), 52
HYBRID_COLUMNS (*hybkit.HybRecord attribute*), 11

I

is_set() (*hybkit.HybRecord method*), 16

K

key (*hybkit.analysis.Analysis attribute*), 45

M

make_id_map_params() (*hybkit.type_finder.TypeFinder static method*), 38
make_out_file_name() (*in module hybkit.util*), 58
make_string_match_params() (*hybkit.type_finder.TypeFinder static method*), 37
matches_hyb_record() (*hybkit.FoldRecord method*), 26
method_hybformat() (*hybkit.type_finder.TypeFinder static method*), 36
method_id_map() (*hybkit.type_finder.TypeFinder static method*), 38
method_is_set() (*hybkit.type_finder.TypeFinder class method*), 35
method_string_match() (*hybkit.type_finder.TypeFinder static method*), 37
methods (*hybkit.type_finder.TypeFinder attribute*), 35
mirna_detail() (*hybkit.HybRecord method*), 15
mirna_details() (*hybkit.HybRecord method*), 15
MIRNA_PROPS (*hybkit.HybRecord attribute*), 12
MIRNA_STR_PROPS (*hybkit.HybRecord attribute*), 12
MIRNA_TYPES (*in module hybkit.settings*), 52
module
    hyb_analyze, 77
    hyb_check, 61
    hyb_eval, 71
    hyb_filter, 65
    hybkit, 8
    hybkit.analysis, 40
    hybkit.errors, 60
    hybkit.plot, 47
    hybkit.settings, 51
    hybkit.type_finder, 34
    hybkit.util, 56

N

not_set() (*hybkit.HybRecord method*), 16

O

open() (*hybkit.CtFile class method*), 32
open() (*hybkit.HybFile class method*), 22
open() (*hybkit.ViennaFile class method*), 29
out_path_exists() (*in module hybkit.util*), 57

P

param_methods (*hybkit.type_finder.TypeFinder attribute*), 35
param_methods_needs_file (*hybkit.type_finder.TypeFinder attribute*), 35
params (*hybkit.type_finder.TypeFinder attribute*), 34
PIE_DEFAULTS (*in module hybkit.plot*), 47
plot_analysis_results() (*hybkit.analysis.Analysis method*), 44
print_report() (*hybkit.HybFoldIter method*), 34
prop() (*hybkit.HybRecord method*), 16

R

read_record() (*hybkit.CtFile method*), 32
read_record() (*hybkit.HybFile method*), 21
read_record() (*hybkit.ViennaFile method*), 29
read_records() (*hybkit.CtFile method*), 33
read_records() (*hybkit.HybFile method*), 21
read_records() (*hybkit.ViennaFile method*), 30
report() (*hybkit.HybFoldIter method*), 34

S

SEGMENT_COLUMNS (*hybkit.HybRecord attribute*), 11
set_custom_method() (*hybkit.type_finder.TypeFinder class method*), 36
set_flag() (*hybkit.HybRecord method*), 13
set_fold_record() (*hybkit.HybRecord method*), 14
set_method() (*hybkit.type_finder.TypeFinder class method*), 35
SET_PROPS (*hybkit.HybRecord attribute*), 11
set_setting() (*in module hybkit.util*), 59
set_settings_from_namespace() (*in module hybkit.util*), 59
settings (*hybkit.analysis.Analysis attribute*), 42
settings (*hybkit.CtFile attribute*), 33
settings (*hybkit.FoldRecord attribute*), 25
settings (*hybkit.HybFile attribute*), 21
settings (*hybkit.HybFoldIter attribute*), 34
settings (*hybkit.HybRecord attribute*), 11
settings (*hybkit.ViennaFile attribute*), 30
static_count_hyb_record_mismatches() (*hybkit.FoldRecord method*), 25
STR_PROPS (*hybkit.HybRecord attribute*), 12

T

target_count() (*in module hybkit.plot*), 49
TARGET_PIE_RC_PARAMS (*in module hybkit.plot*), 47
to_csv() (*hybkit.HybRecord method*), 16
to_csv_header() (*hybkit.HybRecord class method*), 18
to_fasta_record() (*hybkit.HybRecord method*), 17
to_fasta_str() (*hybkit.HybRecord method*), 17
to_fields() (*hybkit.HybRecord method*), 16
to_fields_header() (*hybkit.HybRecord class method*), 18
to_line() (*hybkit.HybRecord method*), 16
to_vienna_lines() (*hybkit.FoldRecord method*), 25
to_vienna_string() (*hybkit.FoldRecord method*), 25
type_count() (*in module hybkit.plot*), 48
type_count_dual() (*in module hybkit.plot*), 49
TYPE_PIE_DUAL_RC_PARAMS (*in module hybkit.plot*), 47
TYPE_PIE_SINGLE_RC_PARAMS (*in module hybkit.plot*), 47
TypeFinder (*class in hybkit.type_finder*), 34
TypeFinder (*hybkit.HybRecord attribute*), 11

V

validate_args() (*in module hybkit.util*), 58
validate_args_exit() (*in module hybkit.util*), 58
vienna_exists() (*in module hybkit.util*), 57
VIENNA_SUFFIXES (*in module hybkit.settings*), 51
ViennaFile (*class in hybkit*), 29

W

write() (*hybkit.HybFile method*), 22
write_analysis_delim_str() (*hybkit.analysis.Analysis method*), 44
write_analysis_results_special() (*hybkit.analysis.Analysis method*), 44
write_fh() (*hybkit.CtFile method*), 33
write_fh() (*hybkit.HybFile method*), 22
write_fh() (*hybkit.ViennaFile method*), 30
write_record (*hybkit.CtFile attribute*), 32
write_record() (*hybkit.HybFile method*), 21
write_record() (*hybkit.ViennaFile method*), 30
write_records (*hybkit.CtFile attribute*), 32
write_records() (*hybkit.HybFile method*), 21
write_records() (*hybkit.ViennaFile method*), 30
